# Red-Light-Driven C(sp^2^)–H Sulfonylation of Anilines Using a Recyclable Benzothiadiazole-Based Covalent Organic Framework

**DOI:** 10.1021/jacs.5c12697

**Published:** 2025-10-13

**Authors:** Saúl Alberca, Akshay M. Nair, Paula Escamilla, Pedro Ferreira, Manuel Souto, Martín Fañanás-Mastral

**Affiliations:** † Centro Singular de Investigación en Química Biolóxica e Materiais Moleculares (CiQUS), 16780 Universidade de Santiago de Compostela, 15782 Santiago de Compostela, Spain; ‡ Department of Chemistry, CICECO-Aveiro Institute of Materials, University of Aveiro, 3810-393 Aveiro, Portugal; § Oportunius, Galician Innovation Agency (GAIN), 15702 Santiago de Compostela, Spain

## Abstract

The limitations of traditional high-energy (NUV or blue) photocatalysis, such as limited penetration in reaction media, off-target reactivity, and health hazards, have spurred the development of seminal red-light-mediated transformations. Despite recent advances, most homogeneous red-light photocatalysts suffer from poor recyclability, and recyclable heterogeneous systems remain underexplored. Herein, we report a red-light-driven C­(sp^2^)–H sulfonylation of anilines using a highly stable benzothiadiazole-based covalent organic framework (Tp-BT COF) as an efficient, recyclable photocatalyst. The reaction proceeds under exceptionally mild conditions, affording sulfonylated products in good to excellent yields with minimal catalyst loading. Notably, the Tp-BT COF retains its catalytic activity over six consecutive cycles. Comparative studies with structurally related COFs highlight the critical role of the BT core in red-light absorption and the superior performance of the AA stacking mode. This work underscores the potential of rationally designed photoactive COFs for sustainable red-light photocatalysis.

## Graphical abstract

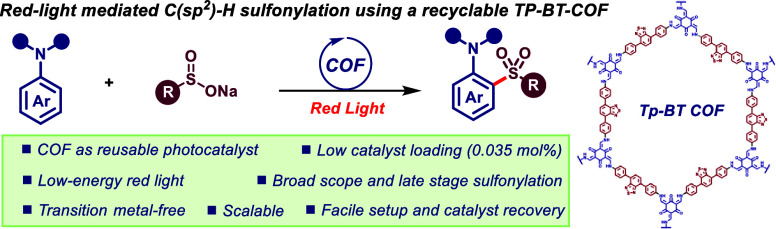

## Introduction

Visible-light photocatalysis has emerged as one of the main pillars of modern organic synthesis.^1,2^ Despite significant advances, these reactions still present some limitations. The high-energy near-ultraviolet (NUV) or blue light typically used in these transformations limits scalability due to light attenuation through the reaction medium, as described by the Beer–Lambert–Bouguer law.^3^ Moreover, the reliance on high-energy (NUV and blue) light introduces health hazards, functional group incompatibilities, and off-target reactivity due to the competitive absorption of such light by substrates and reaction intermediates. In contrast, low-energy red-light-mediated photocatalysis offers a promising alternative, providing higher permeability and milder reaction conditions (Figure 1a).^4^ Consequently, recent years have seen significant interest in red-light photocatalysis, leading to substantial advances in both synthetic and biomedical applications.^5^ In this line, elegant examples of photoredox catalysis using red light have been developed.^6^ However, most red-light photocatalysts are homogeneous in nature, and therefore exhibit limited or no recyclability (Figure 1b). This limitation could be addressed by developing efficient heterogeneous systems. However, heterogeneous red-light photocatalysis remains a largely underexplored area.^7^ To date, these transformations have been limited to the oxidative dimerization of amines^7a–7c^ and the oxidation of sulfides and C­(sp^3^)–H bonds.^7d^ This is likely due to the scarcity of heterogeneous catalysts that combine red-light absorption with redox activity and long-term stability.

Covalent Organic Frameworks (COFs) are crystalline porous polymers that represent an emerging class of highly tunable and recyclable heterogeneous photocatalysts.^8^ Their ordered porosity, high thermal and chemical stability, and tunable electronic properties make them excellent candidates for photocatalysis. In general, their broad absorption range, arising from extensive π-conjugation and low band gaps, especially in 2D COFs, make them promising platforms for red-light photocatalysts. However, to the best of our knowledge, only two examples have been reported that document red-light driven COF photocatalysis for the oxidative dimerization of amines.^7a,7b^ In this context, expanding the chemical space of red-light COF photocatalysis, in terms of both catalyst design and development of value-added transformations, is of significant interest.

Sulfones are valuable organosulfur compounds having broad industrial and biological significance.^9^ Traditional synthesis of sulfones via sulfide oxidation or electrophilic aromatic substitution rely on the use either foul smelling sulfides or exotic reaction conditions.^10^ In recent years, elegant alternative protocols for sulfonylations have emerged relying on sulfur dioxide insertions^11^ and directing group-assisted C–H sulfonylations.^12^ In a seminal work, Willis and co-workers reported a homogeneous iridium-catalyzed oxidative C­(sp^2^)–H sulfonylation of anilines under blue-light irradiation.^13^ Inspired by this work, we envisioned that a heterogeneous photocatalyst capable of mediating a C–H sulfonylation under red-light irradiation would be highly valuable. To this end, we sought to develop a COF photocatalyst capable of promoting this transformation under red light. In particular, photoactive benzothiadiazole (BT)-based COFs have emerged as promising photocatalysts, especially for hydrogen production^14^ and certain oxidative coupling reactions.^15^ The red-light absorption and favorable oxidation potentials of BT-based COF (**Tp-BT**) upon photoexcitation in the presence of an oxidant motivated us to investigate its potential as a catalyst for this reaction. Although the photocatalytic activity of BT-based COFs has been studied for the oxidative dimerization of amines,^15^ their application as heterogeneous red-light photocatalysts has not yet been reported.^16^

Herein, we report an efficient red-light mediated C­(sp^2^)–H sulfonylation of anilines using a recyclable Tp-BT COF photocatalyst (Figure 1c). The reaction affords the corresponding sulfones in good to excellent yields under mild and energy-efficient conditions, employing a remarkably low catalyst loading (as low as 0.035 mol %). The high functional group tolerance (including compatibility with late stage functionalizations) along with the facile scalability and recyclability of the COF photocatalyst are important features of this transformation. Furthermore, a structure–reactivity comparison study with related COF photocatalysts highlights the significance of the BT core for red-light photocatalysis and the importance of the AA-type stacking in the Tp-BT COF.

**Figure 1:**
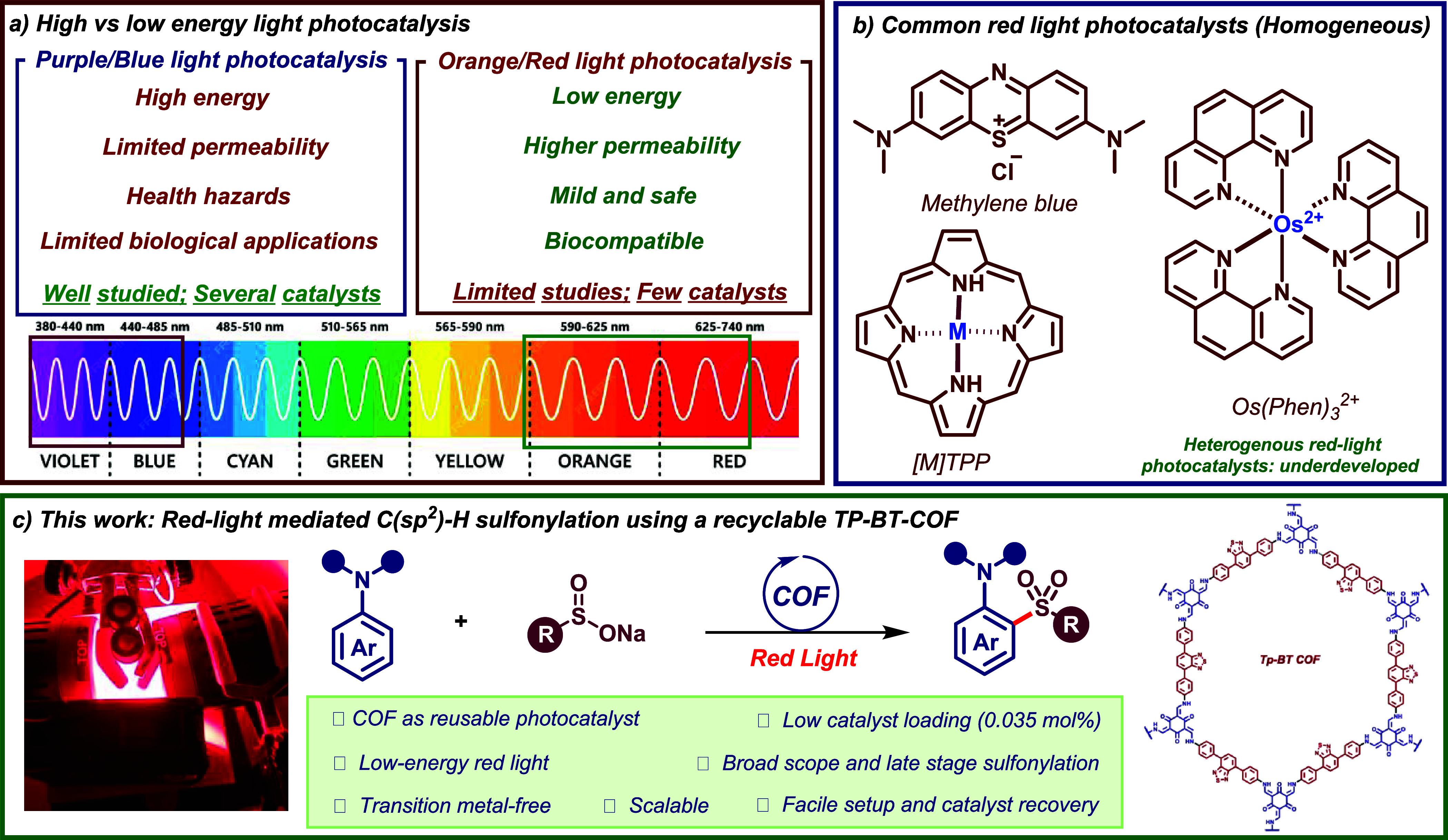
Overview of the work.

## Results and Discussion

At the outset of our study, a series of isoreticular β-ketoenamine-linked COFs with AA stacking mode were synthesized. Accordingly, 4,7-bis­(4-aminophenyl)-2,1,3-benzothiadiazole (BT) and 4,4″-diamino-*p*-terphenyl (TD) were employed as linkers and condensed with 2,4,6-triformylphloroglucinol (Tp). **Tp-BT-AA** and **Tp-TD-AA** COFs (Figure 2a) were obtained by solvothermal reaction at 120 °C using pyrrolidine as the catalyst, in place of aqueous acetic acid, as previously reported,^15b^ a method known to promote the formation of highly crystalline β-ketoenamine COFs.^17^ Powder X-ray diffraction (PXRD) was used to assess the crystallinity and phase purity of the COFs. Both materials exhibited nearly identical diffraction patterns (consistent with isoreticular structures), with the main intense peak at 2θ = 2.7°. Simulated PXRD patterns based on an eclipsed (AA) stacking model closely matched the experimental data (Figure 2b). Furthermore, Pawley refinement of the unit cell parameters yielded an excellent fit with minimal discrepancies (Figures S1 and S2). Fourier transform infrared (FT-IR) spectra were recorded for **Tp-BT-AA** and **Tp-TD-AA** COFs and compared with those of the corresponding building blocks (Figure S3). The disappearance of the characteristic −CHO (1645 cm^–1^) and −NH_2_ (3500–3300 cm^–1^) stretching frequencies, along with the appearance of a new band at 1620 cm^–1^, confirmed the successful polymerization of the precursors. The permanent porosity of both COFs was evaluated by nitrogen adsorption–desorption isotherms at 77 K (Figure 2c). The Brunauer–Emmett–Teller (BET) specific surface areas were calculated using the BETSI analysis,^18^ yielding values of 1351 and 1241 m^2^ g^–1^ for **Tp-BT-AA** and **Tp-TD-AA**, respectively, in agreement with previous reports (Figures S4 and S5).^17,19^ Prior to photocatalytic studies, the light-harvesting properties of the COFs were evaluated using diffuse reflectance spectroscopy (Figure 2d). Optical band gaps were estimated by linear fitting of the absorption onsets in Tauc plots derived from Kubelka–Munk-transformed data (Figure 2e). **Tp-TD-AA** exhibited relatively narrow optical absorbance with an onset at 520 nm and a calculated optical band gap of 2.29 eV. In contrast, **Tp-BT-AA** displayed a red shift of over 100 nm in its absorption onset, resulting in a narrower optical band gap of 2.04 eV. Thermogravimetric analysis (TGA) revealed that both COFs are stable up to 320 °C (Figure S6), well beyond the temperature range of the proposed photocatalytic transformation.

**Figure 2:**
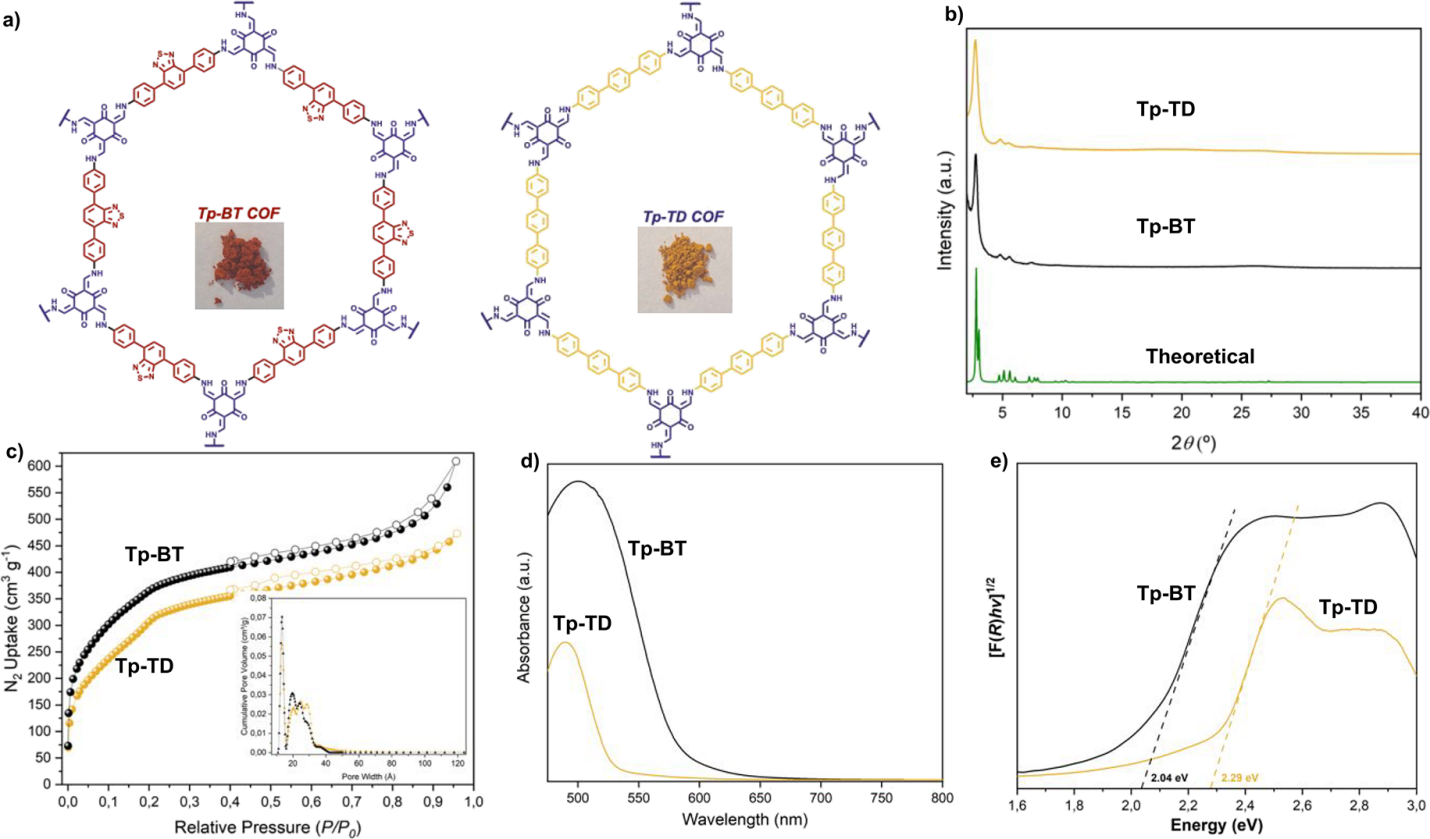
Physicochemical characterization of COF photocatalysts. (a) Molecular structures of **Tp-BT** and **Tp-TD** COFs. (b) Powder X-ray diffraction (PXRD) patterns of **Tp-BT-AA** (experimental and simulated) and **Tp-TD-AA** (experimental). (c) N_2_ adsorption (filled symbols) and desorption (empty symbols) isotherms of **Tp-BT-AA** and **Tp-TD-AA**. The inset shows the pore width distributions. (d) Absorbance spectra of **Tp-BT-AA** and **Tp-TD-AA**. (e) Normalized Tauc plot of the Kubelka–Munk-transformed data for **Tp-BT-AA** and **Tp-TD-AA**. Dashed lines indicate linear fits to the absorption onsets.

The optimal band gap and red-light absorption of **Tp-BT-AA** COF made it a promising candidate for our studies. We chose the reaction between *N,N*-dimethyl-*p*-toluidine **1a** and sodium toluyl sulfinate **2a** as the model reaction to evaluate the catalytic activity of this COF. Screening of the reaction parameters (Table 1 and Supporting Information) led to the optimized conditions involving **Tp-BT-AA** COF (0.07 mol %), K_2_S_2_O_8_ (3 equiv) and phase-transfer agent (TBA)­HSO_4_ (0.2 equiv) in MeCN:H_2_O (10:1) under irradiation by two red LED lamps (660 nm) for 12 h. Under these conditions, product **3a** was isolated in 85% yield (Table 1, entry 1). Irradiation using a single red LED lamp resulted in lower efficiency (entry 2). The catalytic activity of the **Tp-BT-AA** COF was efficient across a broad wavelength range (entries 3–5). Notably, **3a** was obtained in 95% yield within 2 h under green LED (525 nm) light irradiation (entry 4). Remarkably, even far-red LED irradiation (740 nm) provided the product, albeit in diminished yield (entry 6). It is important to note that the crystallinity of **Tp-BT-AA** COF, modulable depending on the modulator used in the synthesis (Figure S7), does not have a significant influence on catalytic performance (see Section 4c in the Supporting Information). **Tp-BT** COFs with AB and ABC stacking modes were also synthesized (see Supporting Information for details) to investigate the influence of stacking on the catalytic performance. The AA stacking mode for **Tp-BT** COF was found to be critical, as the same COF with AB and ABC stackings was catalytically inefficient under the same conditions (entries 7 and 8). Since the optical properties of the three COFs are similar (Figure S10), the lower catalytic performance could be attributed to the reduced porosity of the **Tp-BT** COFs with AB and ABC packing (Figure S11), which may limit the diffusion of reactants. The chemical structure of the COF photocatalyst also proved to be a key factor. The **Tp-TD-AA** COF, lacking the BT core, delivered **3a** in a 93% yield under green light (entry 9) but was ineffective under orange (595 nm) and red (660 nm) irradiation (entries 10 and 11). This highlights the superior red-light harvesting ability of the BT core. Finally, control experiments confirmed that both the COF photocatalyst and light irradiation are essential for the transformation, as no product was formed in their absence (entry 12).

**Table 1:**
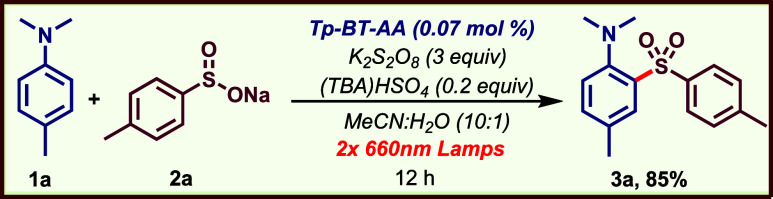

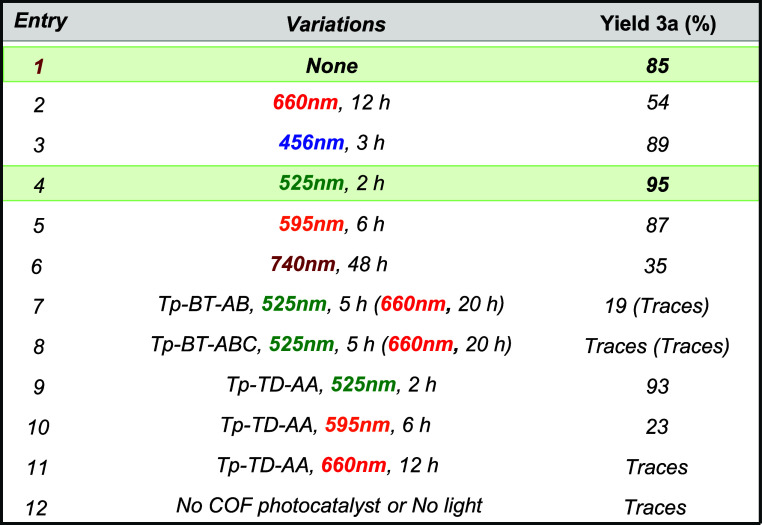
Reaction Optimization^a^,^b^

a Reaction conditions: **1a** (0.4 mmol), **2a** (1.2 mmol), **Tp-BT-AA** (0.07 mol %), K_2_S_2_O_8_ (1.2 mmol), (TBA)­HSO_4_ (0.08 mmol) in MeCN/H_2_O 10:1 (4 mL), LEDs, rt, air.

b Isolated yields.

Having identified the optimal COF photocatalyst and reaction conditions, we next explored the scope of this heterogeneous photocatalytic transformation (Figure 3). A variety of sulfinates (**2**) and aniline derivatives (**1**) were tested under both red and green light irradiations. Phenyl and aryl sulfinates bearing either electron-donating or electron-withdrawing substituents at the *para* position were well tolerated, delivering the corresponding sulfones **3b**–**d** in excellent yields. Both 1- and 2-naphthyl sulfinates (**3e** and **3f**) also proved to be efficient substrates. Interestingly, these substrates showed better performance under green or red light compared to blue light, further highlighting the advantage of using low-energy irradiation. Notably, sterically hindered mesityl sulfinate (**3g**) was also compatible, although diminished yield was observed, likely due to the more encumbered nature of this reagent. C–H sulfonylation of aniline **1a** with alkyl sulfinates (including both primary and secondary derivatives) afforded products **3h**–**3j** in good to excellent yields.

**Figure 3:**
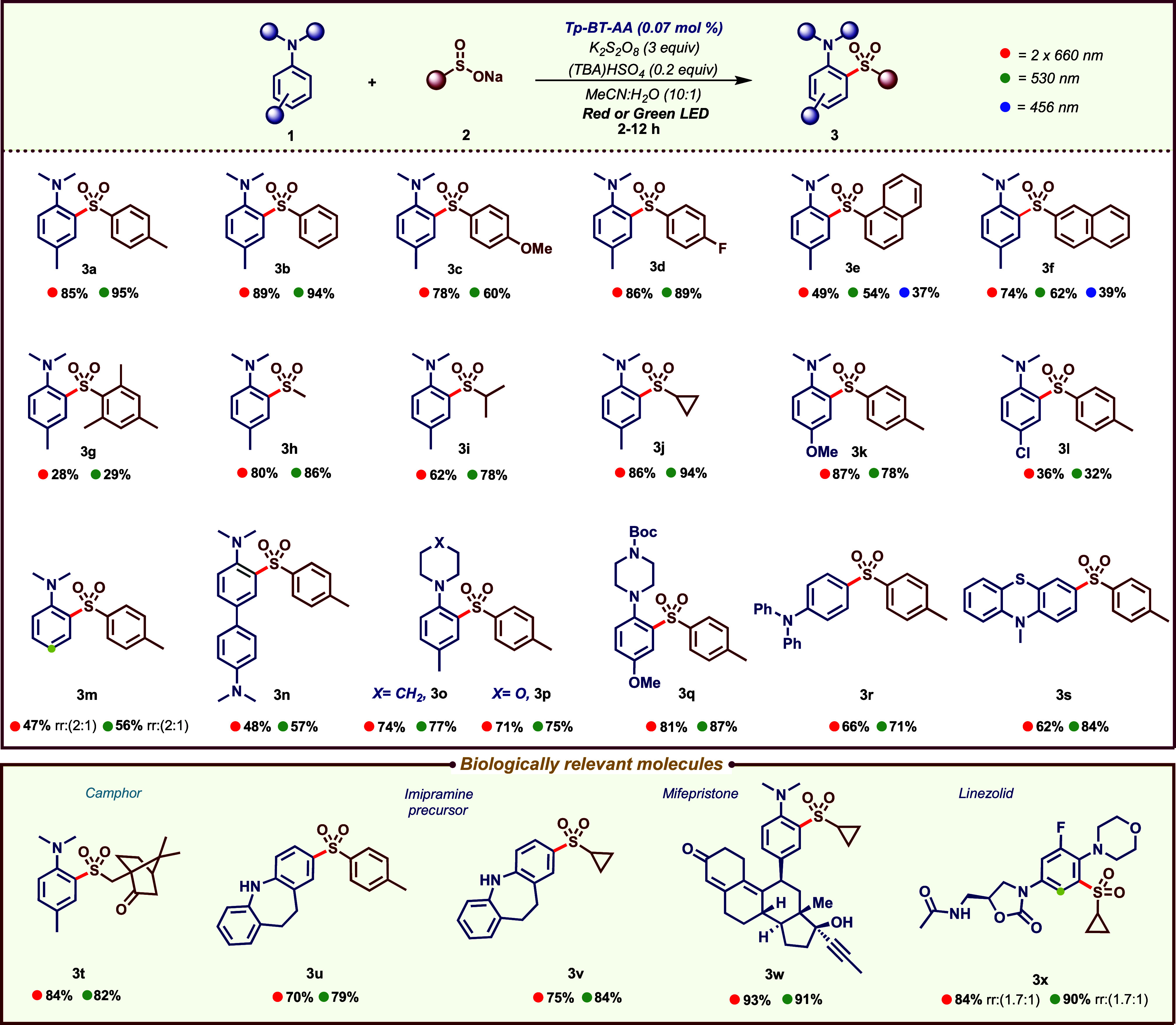
Substrate scope of COF photocatalyzed C­(sp^2^)–H sulfonylation of anilines. ^a^ Reaction conditions: **1** (0.4 mmol), **2** (1.2 mmol), **Tp-BT-AA** (0.07 mol %), K_2_S_2_O_8_ (1.2 mmol), (TBA)­HSO_4_ (0.08 mmol) in MeCN/H_2_O 10:1 (4 mL), stirred at room temperature, under air atmosphere and irradiated with light (525 nm, 44 W or 2x 660 nm, 44 W). ^b^ Isolated yields.

We then turned to the evaluation of various aniline derivatives. While the electron-donating OMe group (**3k**) was well tolerated at the *para*-position, the presence of a Cl group (**3l**) at the same position led to a diminished yield. Unsubstituted *N,N-*dimethylaniline led to product **3m** as a 2:1 (*ortho:para*) mixture of regioisomers. Biphenyl diamine underwent selective monosulfonylation to afford product **3n** in good yield. Notably, biologically relevant heterocycles such as *N*-toluyl morpholine (**3o**) and *N*-anisyl piperidine (**3p**) provided the corresponding sulfones in excellent yields. Triphenyl aniline underwent *mono*-sulfonylation at the *para* position to deliver **3r**. Interestingly, C–H sulfonylation of the organic photosensitizer methyl-phenothiazine was feasible, selectively providing product **3s**.

To demonstrate the synthetic versatility of our protocol, late-stage functionalization of naturally occurring and biologically relevant molecules was performed. Camphor sulfinate was found to be suitable, delivering **3t** in an excellent yield. Furthermore, C–H sulfonylation of drug molecules, such as Imipramine core (**3u** and **3v**), Mifepristone (**3w**) and Linezolid (**3x**), was successfully accomplished.

Notably, the present protocol affords consistently higher product yields compared to those reported by Willis,^13^ while operating with lower photocatalyst loading and reduced sulfinate equivalents.^20^ Moreover, the high calculated apparent quantum yield (AQY = 75.5%) of the reaction further underscores the high catalytic performance of the **Tp-BT-AA** COF (see Section 10 in the Supporting Information).

The reaction could be easily scaled up to a 5 mmol scale under slightly modified reaction conditions (Figure 4a). Remarkably, the COF photocatalyst loading could be further reduced to 0.035 mol %. This corresponds to only 5 mg of **Tp-BT-AA** COF required to produce 1.2 g of **3a**, highlighting the high catalytic efficiency of this COF. Also noteworthy is that the **Tp-BT-AA** COF could be easily recovered and reused with no loss in the yield of **3a** after six catalytic cycles (Figure 4b, top). Analysis of the recovered COF by PXRD, FT-IR and scanning electron microscopy (SEM) confirmed that its structure and morphology remained unchanged compared to the pristine material (Figures 4b, bottom and S14). Although the BET surface area of the recovered material decreased, the pore size distribution remained unchanged (Figure S15), ruling out pore collapse. This demonstrates the exceptional stability of **Tp-BT-AA** COF under our reaction conditions and emphasizes the potential for additional recycling and reuse.

**Figure 4:**
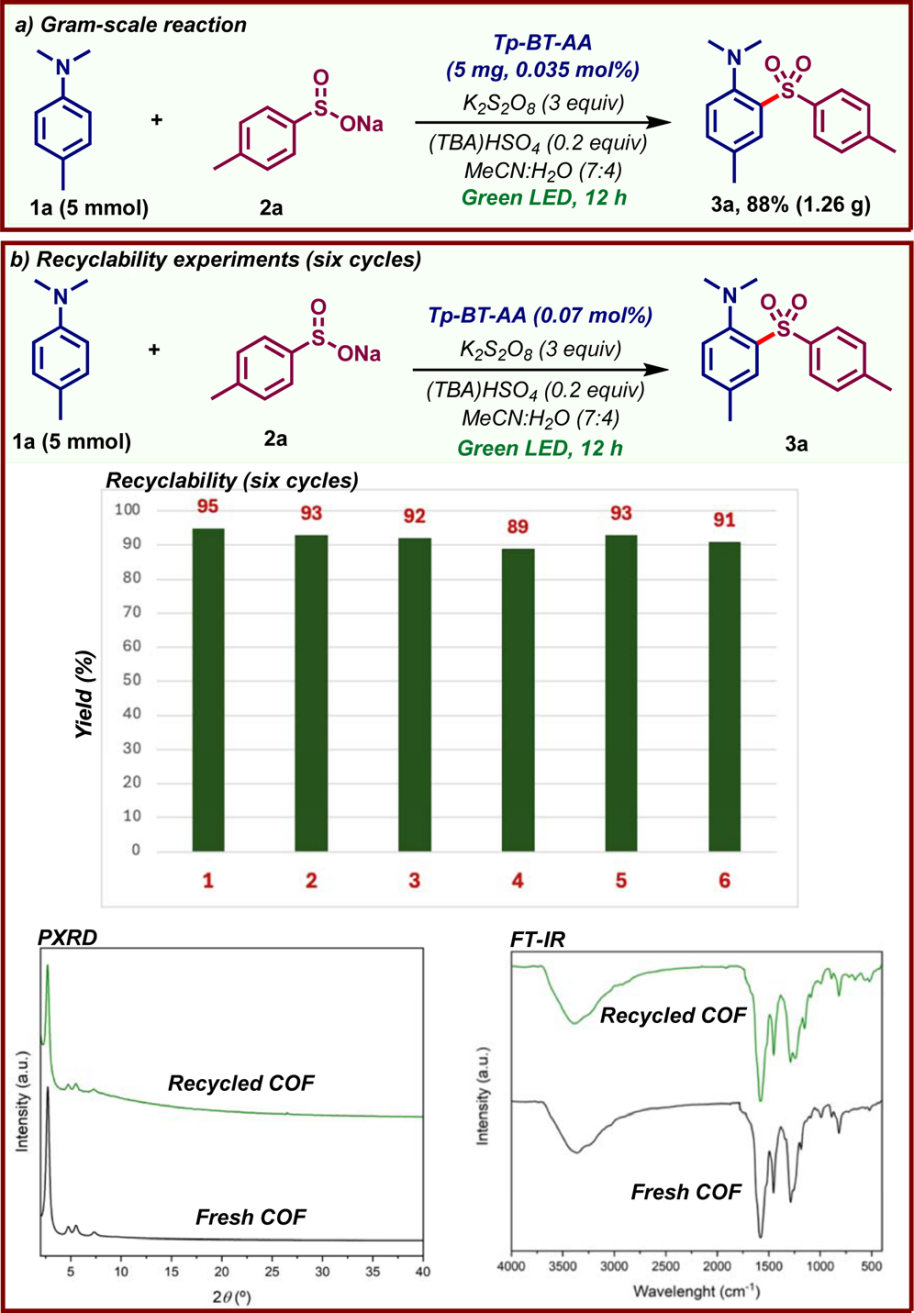
Scale-up of the reaction and photocatalyst recyclability studies.

Preliminary mechanistic studies provide important insight into the reaction pathway (Figure 5). No product formation was observed in the presence of the radical scavenger TEMPO, indicating that the reaction proceeds via stable radical intermediates. The reaction also failed to deliver **3a** in the presence of the single-electron quencher (AgNO_3_) and hole quencher (KI), indicating the formation of electron–hole pairs within the COF.^21^ A light on–off study revealed that the reaction shuts off in the dark and only proceeds under light irradiation, indicating the absence of chain propagation steps. Based on these studies and previous reports,^13,15^ we propose the mechanism depicted in Figure 5c. Photoexcitation of **Tp-BT-AA** COF promotes electron transfer from the valence band (VB) to the conduction band (CB), forming electron–hole pairs. Based on the Mott–Schottky plot^15^ and the optical band gap of **Tp-BT-AA**, the VB and CB were determined to be +1.26 and −0.79 eV vs NHE, respectively. Single-electron transfer from the CB to persulfate occurs, while both the sulfinate (*E*_ox_ **2a** =+0.30 eV vs SCE)^22^ and amine (*E*_ox_ **1d** = +0.70 eV vs SCE)^23^ undergo single electron oxidation by the holes at the VB (+1.26 eV vs SCE), generating radical cation **A** and sulfonyl radical **B**. The subsequent coupling of these radical species followed by deprotonation would furnish the product (**3a**).

**Figure 5:**
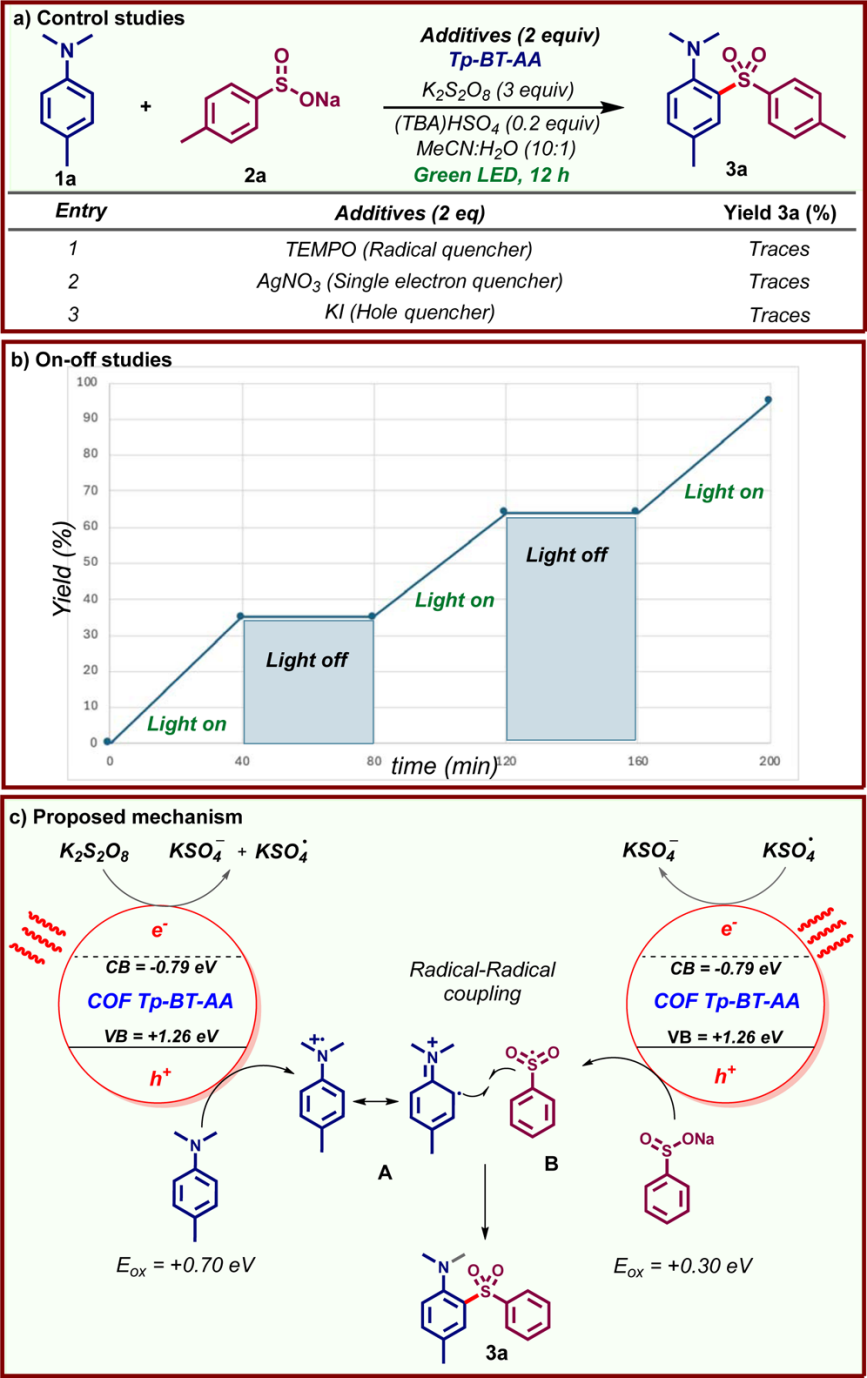
Preliminary mechanistic studies and plausible mechanism.

## Conclusions

In conclusion, we have developed an efficient protocol for the C­(sp^2^)–H sulfonylation of anilines under red-light irradiation using the **Tp-BT-AA** COF as a photocatalyst. The reaction proceeds under very mild conditions, delivering the sulfone products in good to excellent yields. Remarkably, the protocol requires extremely low photocatalyst loading. Furthermore, no significant loss in catalytic activity was observed even after six consecutive catalytic cycles. A comparative study of related COFs highlighted the superior red-light harvesting ability of the BT core, as well as the catalytic significance of the AA stacking mode in the **Tp-BT** COF.

## Supplementary Material



## Notes

List of starting materials, experimental procedures, powder X-ray diffraction, FT-IR, TGA, N_2_ adsorption isotherm, photocatalytic optimization studies, and compound characterization data (PDF)
