# Supplementary material for: Red-Light-Driven C(sp2)–H Sulfonylation of Anilines Using a Recyclable Benzothiadiazole-Based Covalent Organic Framework
Source: J Am Chem Soc. 2025 Oct 13;147(43):39582–9. doi: 10.1021/jacs.5c12697 (PMC12576818; doi:10.1021/jacs.5c12697)

Supporting Information

Red-light Driven C*(sp*^{2}*)*-H Sulfonylation of Anilines Using a Recyclable Benzothiadiazole-based Covalent Organic Framework

Saul Alberca,[a] Akshay M. Nair,[a] Paula Escamilla,[a] Pedro Ferreira,[b] Manuel Souto,*[a,b,c] and Martín Fañanás-Mastral*[a,c]

[a]*Centro Singular de Investigación en Química Biolóxica e Materiais Moleculares (CiQUS), Universidade de Santiago de Compostela, 15782 Santiago de Compostela, Spain*

[b]*Department of Chemistry, CICECO-Aveiro Institute of Materials, University of Aveiro, 3810-393 Aveiro, Portugal*

[c]*Oportunius, Galician Innovation Agency (GAIN), 15702 Santiago de Compostela, Spain*

Correspondence to:

manuel.souto.salom@usc.es

martin.fananas@usc.es

Table of contents

1. General information S3

2. Synthesis and characterization of Tp-BT-AA COF, Tp-BT-AB, Tp-BT-ABC and Tp-TD-AA COF S5

3. List of starting materials S14

4. Optimization studies S16

5. General procedure for the photocatalytic sulfonylation of anilines (GP-1) S18

6. Large scale reaction S19

7. Photocatalyst recyclability studies S21

8. Control experiments S22

9. Compound characterization S23

10. Apparent quantum yield (A.Q.Y.) calculations S35

11. Comparison of this work with the homogeneous Ir(III) photocatalyzed protocol S36

12. References S37

13. NMR spectra of compounds S39

1. General information

All reactions were performed under air atmosphere using borosilicate glass screw cap vials of 10 mL, unless otherwise noted. Solvents were dried using an MBraun SPS 800 system. All chemicals were purchased from Acros Organics Ltd., Active Scientific, Aldrich Chemical Co. Ltd., Alfa Aesar, Apollo, BLD pharma, Fluorochem Ltd. or TCI Europe N.V. chemical companies and used without further purification, unless otherwise noted

Light Source: The light lamps used are white CFL (55 W) and Kessil PR 160: 456 nm (50 W), 525 nm (44 W), 595 nm (44 W), 660 nm (44 W) and 740 nm (44 W).

Analytical thin layer chromatography (TLC) was performed on aluminum backed plates (1.5 × 5 cm) pre-coated (0.25 mm) with silica gel (Merck, Silica Gel 60 F254). Compounds were visualized by exposure to 254 nm UV light or/and by dipping the plates in solutions of $KMnO_4$ or phosphomolibdic acid (PMA) stains followed by heating.

Flash column chromatography was performed on silica gel (Merck Kieselgel 60, 230-400 mesh) without previous deactivation or using Buchi Pure Chromatography System with FlashPure EcoFlex Silica 12 or 40 g.

^{1}H, ^{13}C, and ^{19}F NMR experiments were carried out using a Bruker AVIII-500 MHz or a Varian Mercury 300MHz NMR spectrometer. Chemical shift values are reported in ppm with the solvent resonance as the internal standard ($CHCl_3$: δ 7.26 for ^{1}H, δ 77.2 for ^{13}C). Coupling constants (*J*) are given in Hertz (Hz). Multiplicities are reported as follows: s (singlet), brs (broad singlet), d (doublet), dd (double doublet), t (triplet), dt, (double triplet), hept (heptuplet) and m (multiplet).

Optical rotations were measured on a JASCO P-2000 polarimeter.

High Resolution Mass spectrometry was carried out on a Bruker microTOF spectrometer using APCI-FIA.

Melting points were recorded using a Buchi B-545 melting point apparatus and are uncorrected.

Scanning electron microscopy (SEM) images were recorded using a FESEM equipment (ultra plus, Zeiss, Germany).

Powder X-ray diffraction (XRD) patterns were recorded using a Rigaku MiniFlex 600-C X-ray diffractometer using Cu Kα radiation (λ = 1.54056 Å). The X-ray tube was

operated at a voltage of 40 kV and a current of 15 mA. Data were collected in the ca. 2º ≤ 2θ ≤ 40º range.

Gas sorption measurements were recorded ex-situ on a Micromeritics 3Flex apparatus. The samples were degassed overnight at 100 ºC and 10^{-6} Torr prior to analysis. BET surface values were calculated from the N_2 isotherms using BETSI[1] and pore size distributions were obtained using the non-local density functional theory (NLDFT) method.

Thermogravimetric analysis (TGA) of the powder samples was conducted employing a TA Instruments Q5000 IR thermobalance. The TGA measurements involved a general heating profile ranging from 25 to 800 °C, with a heating rate of 5 °C min^{-1} under an N_2 atmosphere using a gas flow rate of 25 mL min^{-1}.

FT-IR measurements were conducted employing a PerkinElmer Spectrum Two spectrometer equipped with Attenuated Total Reflection (ATR) capability. The dried powders were positioned on the ATR window for analysis within the wavenumber range of 400 to 4000 cm^{-1}.

The UV-vis-NIR absorption and diffuse reflectance spectra of the samples were measured using a Lambda 950 dual-beam spectrometer (PerkinElmer) and Reflectance FLEX Pack (Sarspec). The diffuse reflectance spectra are reported as the Kubelka-Munk transform, where $F(R) = (1-R)^2 /2R$. The direct optical band gaps of these materials were determined from respective Tauc plots.

2. Synthesis and characterization of Tp-BT-AA COF, Tp-BT-AB, Tp-BT-ABC and Tp-TD-AA COF

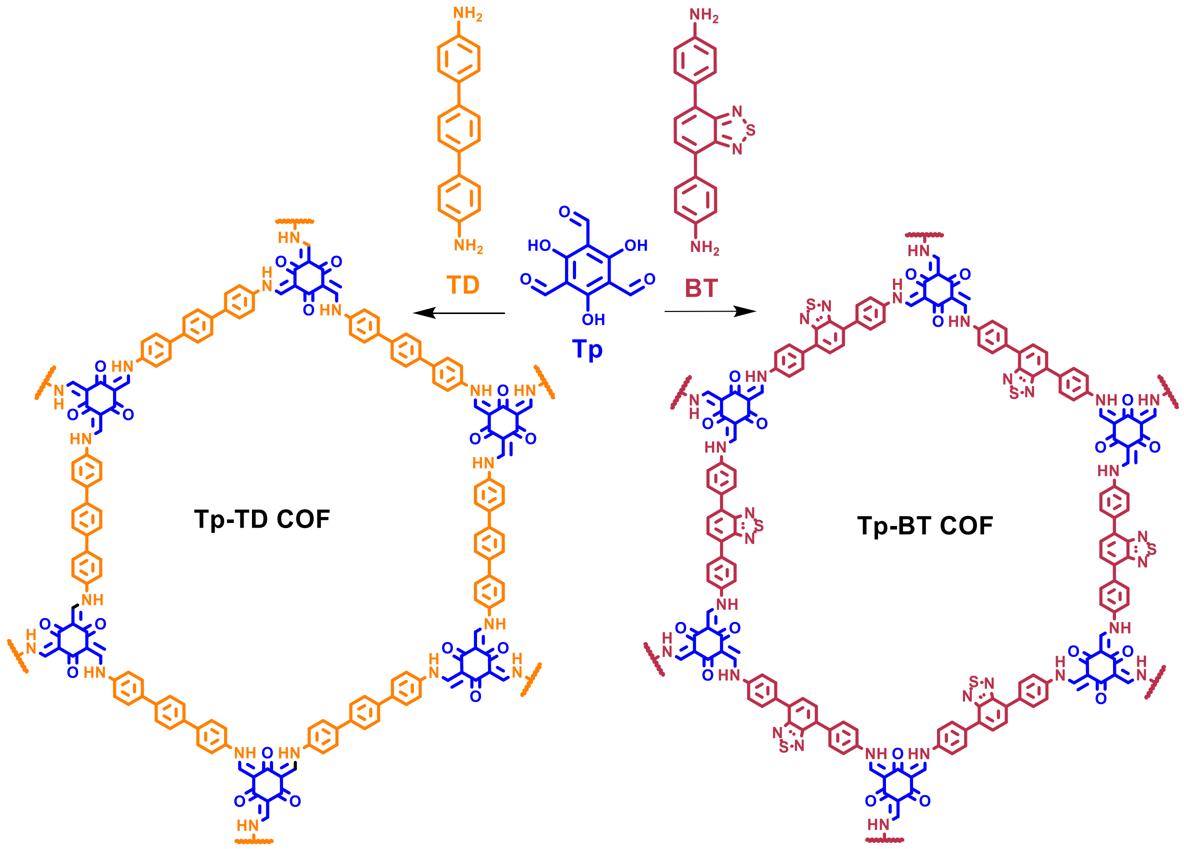

Scheme 1. Synthesis of **Tp-TD COF** and **Tp-BT COF**.

Tp-BT-AA,[2] **Tp-BT-AB**,[3] **Tp-BT-ABC**,[3] and **Tp-TD-AA**[2] COFs were synthesized adapting previously reported procedures.

Synthesis of Tp-BT-AA COF:

16.8 mg of 2,4,6-Triformylphloroglucinol (Tp) (0.080 mmol) and 38.1 mg of 4,7-bis(4-aminophenyl)-2,1,3-benzothiadiazole (BT) (0.12 mmol) were introduced into a 10-mL Schlenk tube. Then, *o*-dichlorobenzene (0.9 mL), *n*-BuOH (0.1 mL), and pyrrolidine (0.02 mL) were added to the mixture that was sonicated for 10 min to get a homogeneous dispersion. Then, the tube was flash-frozen at 77K (liquid N_2 bath) and degassed by three freeze–pump thaw cycles. The tube was placed in an oven at 120 °C for 3 days (5h ramping). The precipitate was filtered and washed with THF and acetone to obtain **Tp-BT-AA** as a red powder (42.2 mg, 56%).

Synthesis of Tp-BT-AB COF:

10.5 mg of Tp (0.050 mmol) and 24.0 mg of BT (0.075 mmol) were introduced into a 10-mL Schlenk tube. Then, *o*-dichlorobenzene (1.0 mL), *n*-BuOH (1.0 mL), and 6 M aqueous acetic acid

(0.2 mL) were added to the mixture that was sonicated for 5 min to get a homogeneous dispersion. Then, the tube was flash-frozen at 77K (liquid N_2 bath) and degassed by three freeze−pump thaw cycles. The tube was then placed in an oven at 120 °C for 3 days (5h ramping). The precipitate was filtered and washed with THF and acetone to obtain **Tp-BT-AB** as a red powder (32.1 mg, 49%). The PXRD pattern matches the one previously reported.[3]

Synthesis of Tp-BT-ABC COF:

10.5 mg of Tp (0.050 mmol) and 24.0 mg of BT (0.075 mmol) were introduced into a 5-mL vial equipped with a stirrer. Then, *o*-dichlorobenzene (1.0 mL), *n*-BuOH (1.0mL), and 6 M aqueous acetic acid (0.2 mL) were added to the mixture, followed by ultrasonic dispersion for 1 min and stirring at room temperature for 3 days. After that, the precipitate was filtered and washed with THF and acetone to obtain **Tp-BT-ABC** as a red powder (25.5 mg, 41%). The PXRD pattern matches the one previously reported.[3]

Synthesis of Tp-TD-AA COF:

16.8 mg of Tp (0.080 mmol) and 31.0 mg of 4,4''-diamino-p-terphenyl (TD) (0.12 mmol) were introduced into a 10-mL Schlenk tube. Then, *o*-dichlorobenzene (0.9 mL), *n*-BuOH (0.1 mL), and pyrrolidine (0.02 mL) were added to the mixture that was sonicated for 5 min to get a homogeneous dispersion. Then, the tube was flash-frozen at 77K (liquid N_2 bath) and degassed by three freeze−pump thaw cycles. The tube was then placed in an oven at 120 °C for 3 days (5h ramping). The precipitate was filtered and washed with THF and acetone to obtain **Tp-TD-AA** as a yellow powder (47.1 mg, 85%).

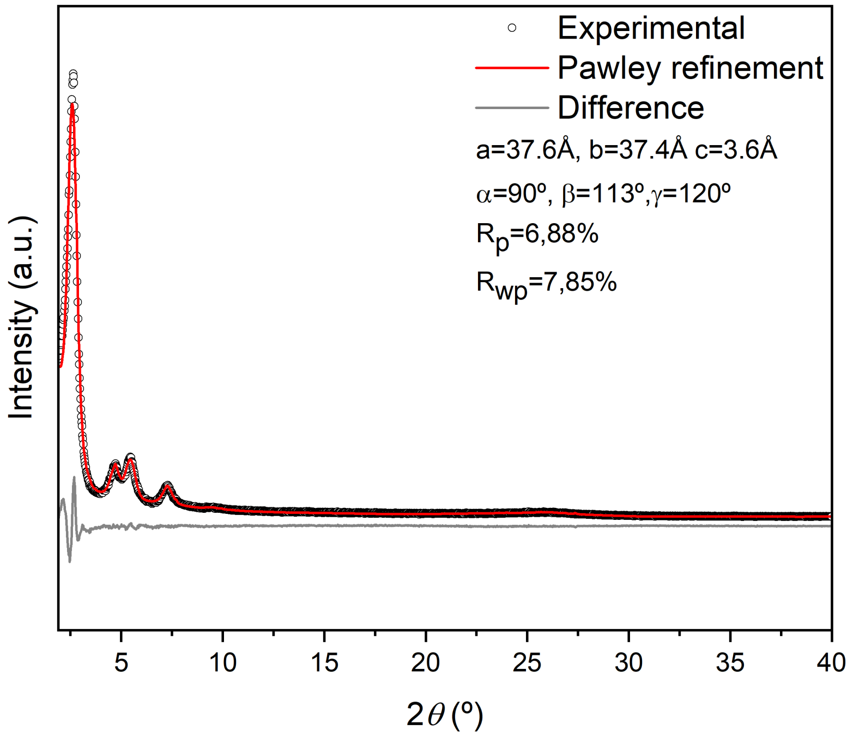

Figure S1. PXRD pattern and Pawley refinement of **Tp-BT-AA** COF.

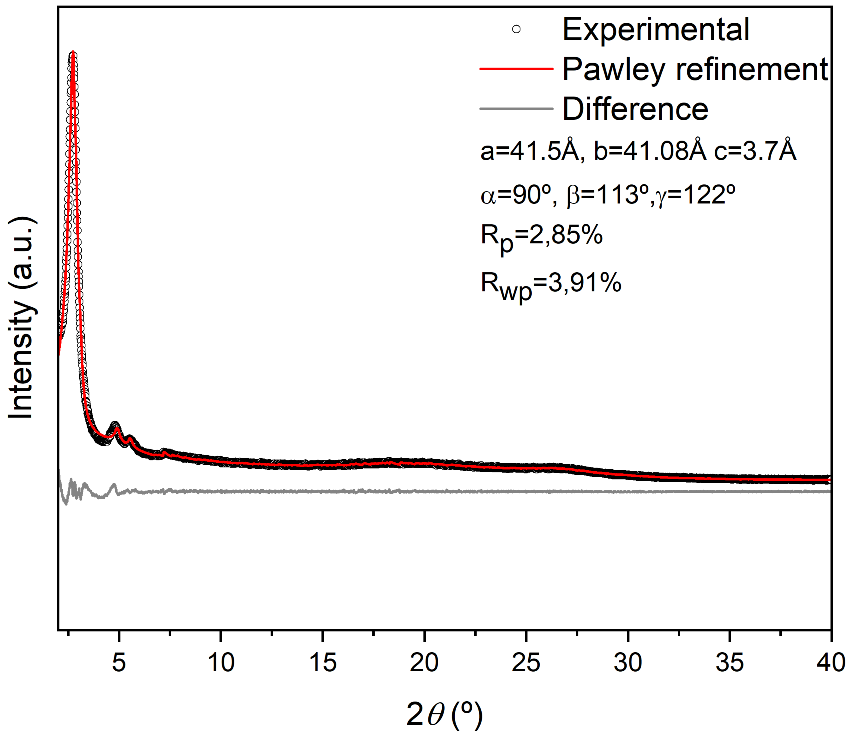

Figure S2. PXRD pattern and Pawley refinement of **Tp-TD-AA** COF.

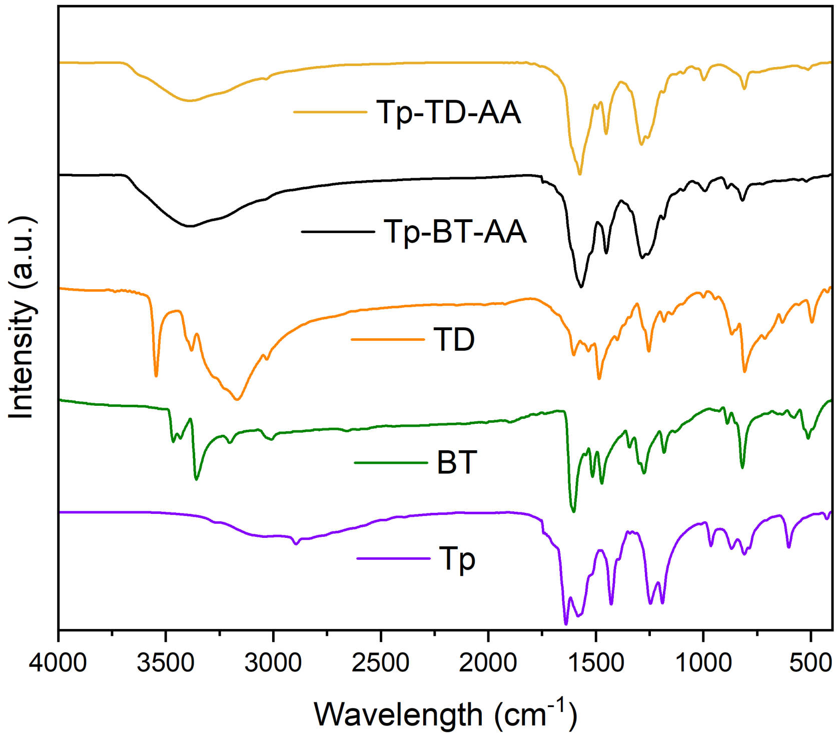

Figure S3. FT-IR spectra of Tp, BT, TD, **Tp-BT-AA** and **Tp-TD-AA** COF.

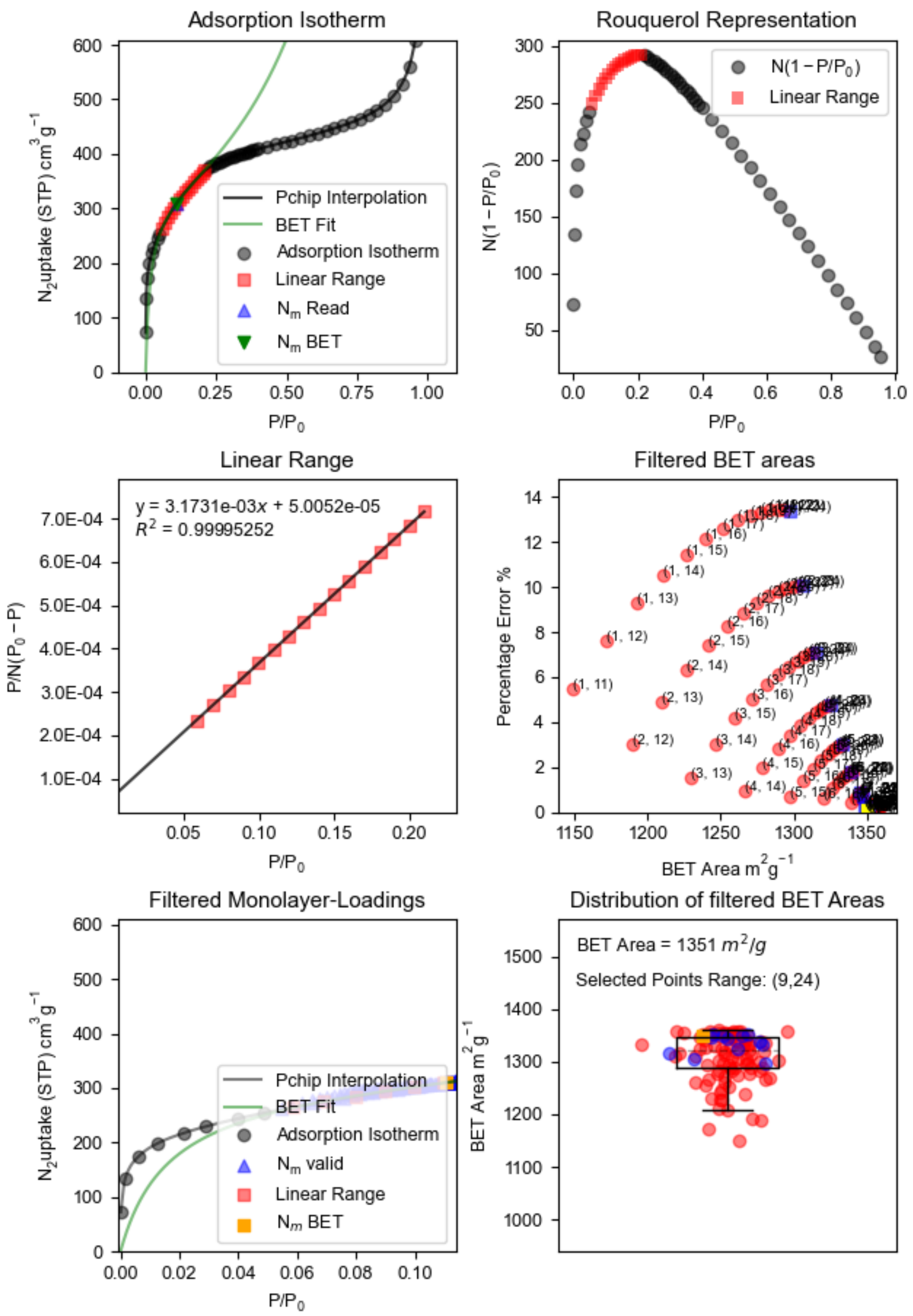

Figure S4. BETSI regression diagnostics for **Tp-BT-AA** COF.

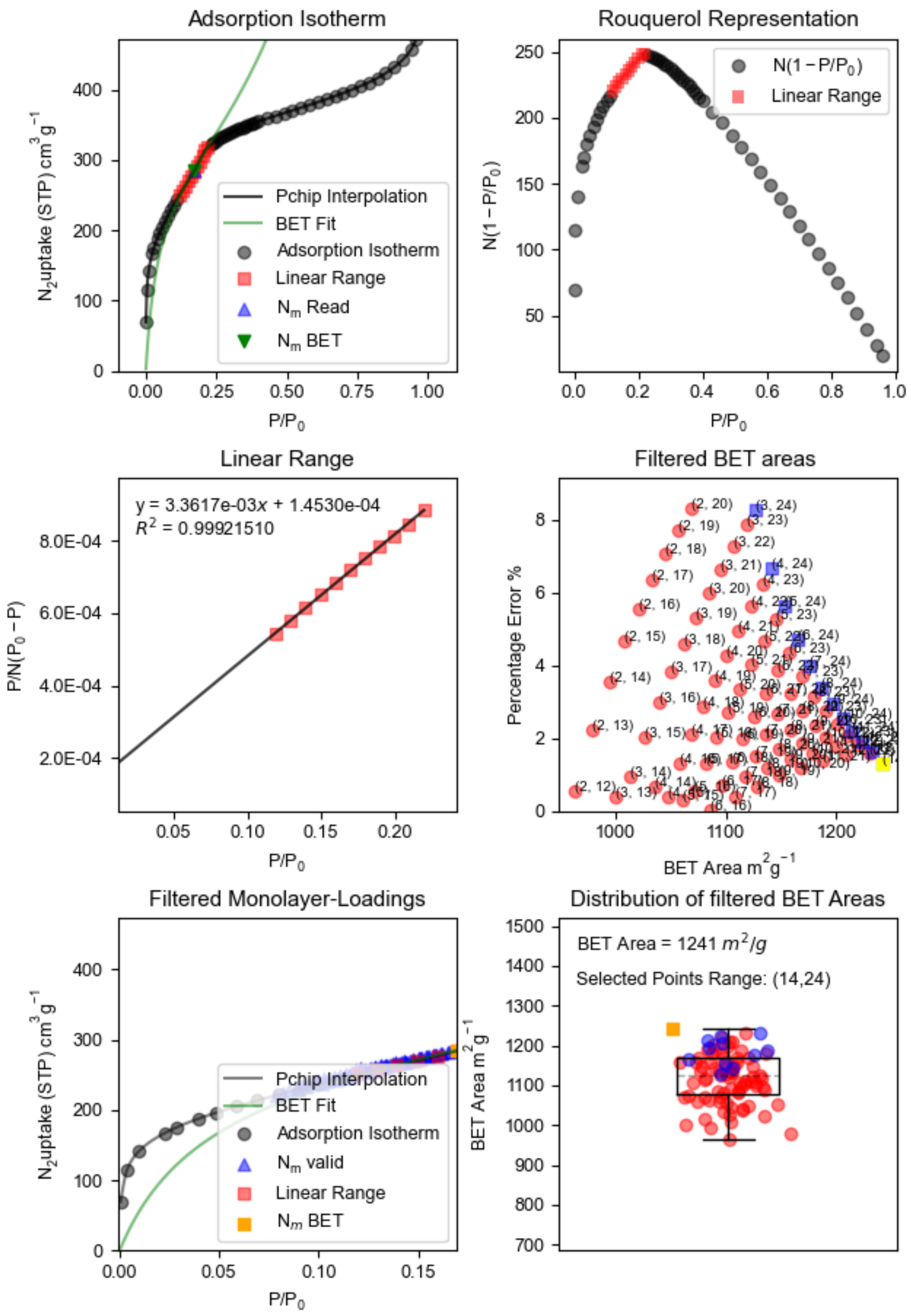

Figure S5. BETSI regression diagnostics for **Tp-TD-AA** COF.

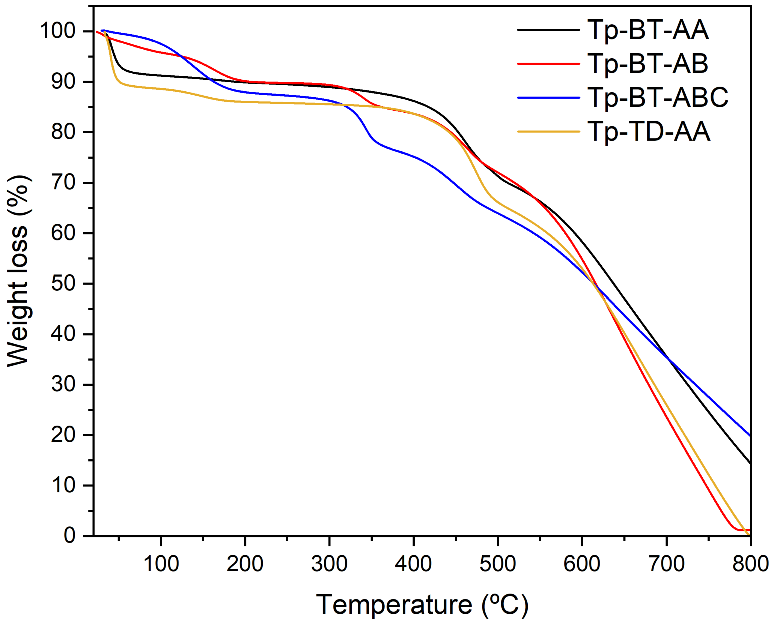

Figure S6. TGA profile of **Tp-BT-AA**, **Tp-BT-AB**, **TP-BT-ABC**, **Tp-TD**.

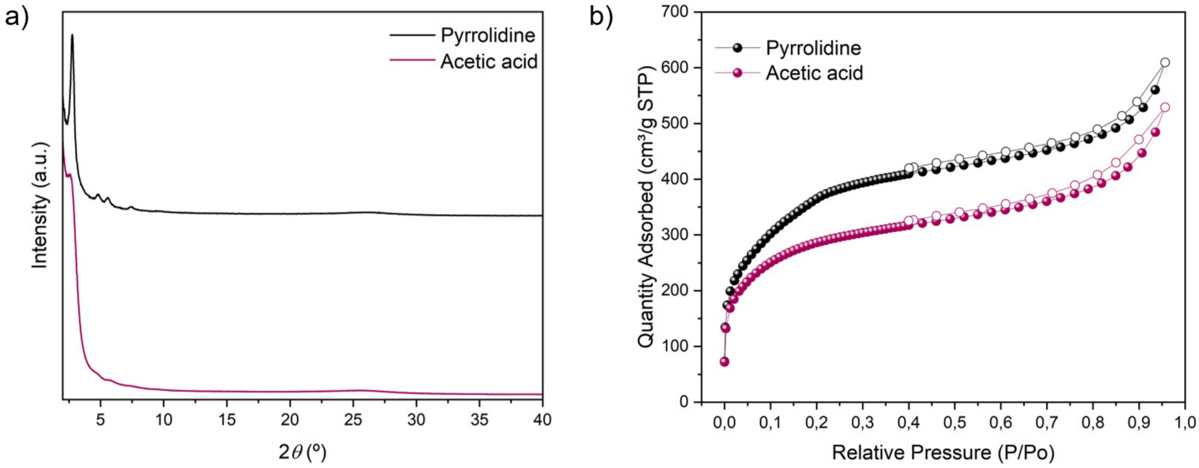

Figure S7. a) Experimental powder X-ray diffraction (PXRD) patterns, and b) N_2 adsorption (filled symbols) and desorption (empty symbols) isotherms of **Tp-BT-AA** synthesized using acetic acid or pyrrolidine as modulator.

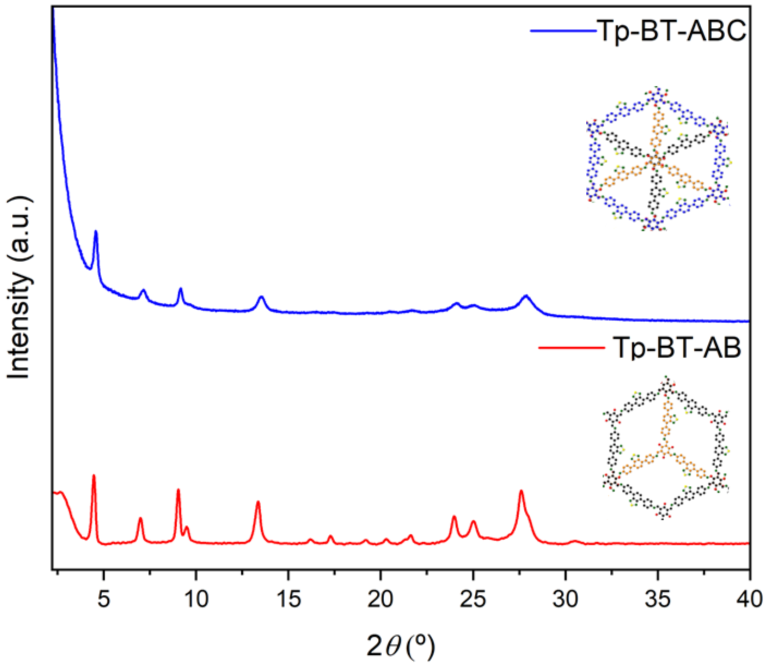

Figure S8. Experimental PXRD pattern of **Tp-BT-AB** (red lines) **and Tp-BT-ABC** (blue lines).

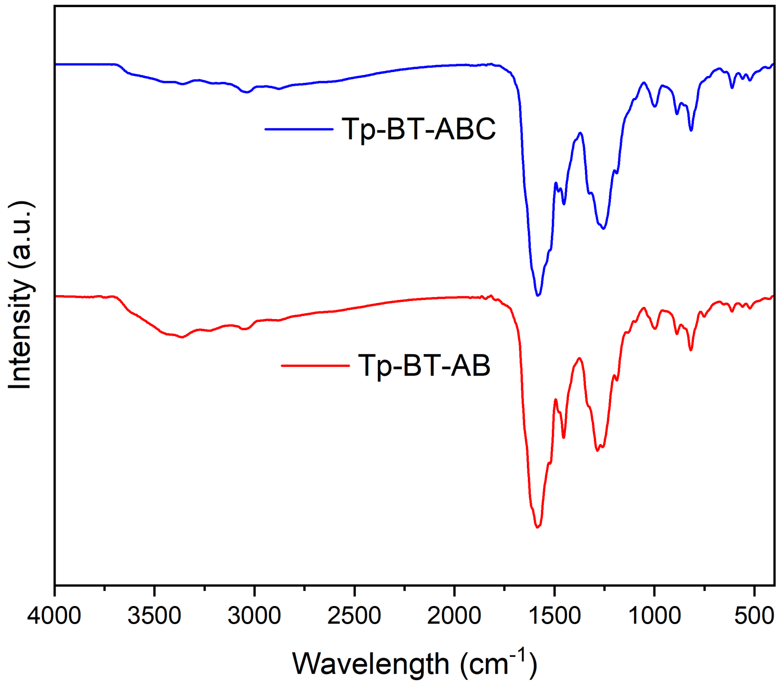

Figure S9. FT-IR spectra of **Tp-BT-AB** COF and **Tp-BT-ABC** COF.

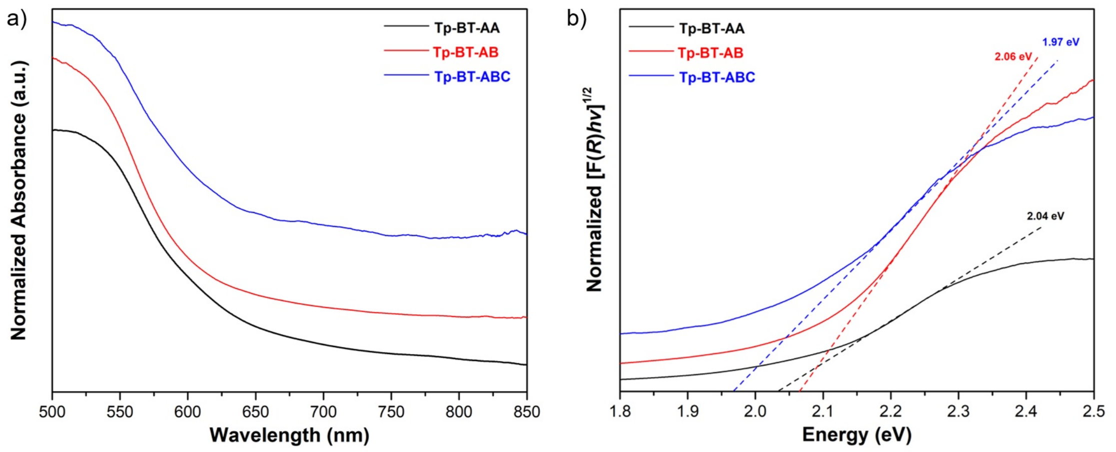

Figure S10. a) Absorbance spectra and b) normalized Tauc plot of the Kubelka–Munk-transformed data for **Tp-BT-AA**, **Tp-BT-AB** and **Tp-BT-ABC**.

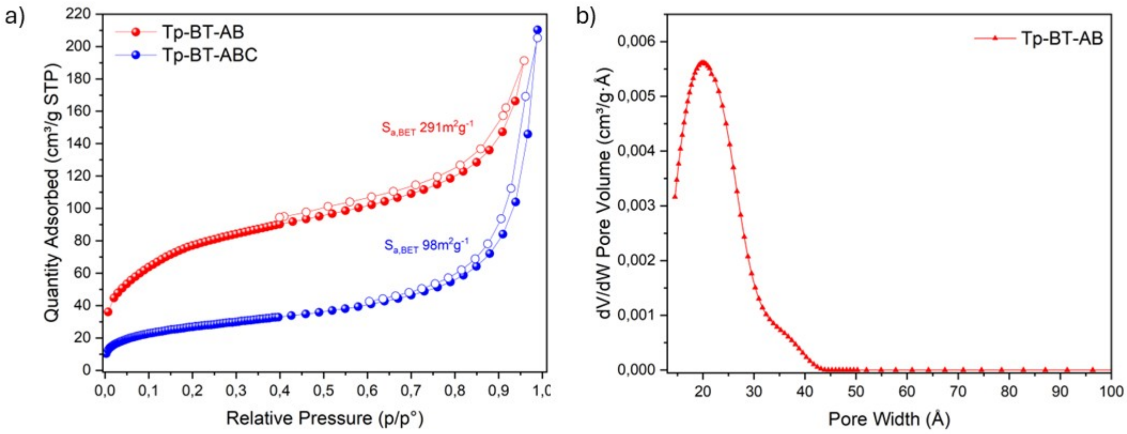

Figure S11. a) N_2 adsorption (filled symbols) and desorption (empty symbols) isotherms of **Tp-BT-AB** and **Tp-BT-ABC**, and b) pore size distribution of **Tp-BT-AB**.

3. List of starting materials

a) Aniline derivatives **1**

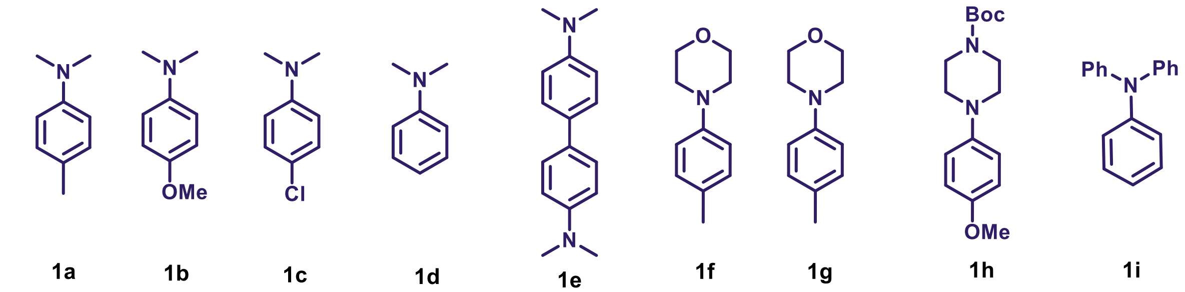

1a 1b 1c 1d 1e 1f 1g 1h 1i

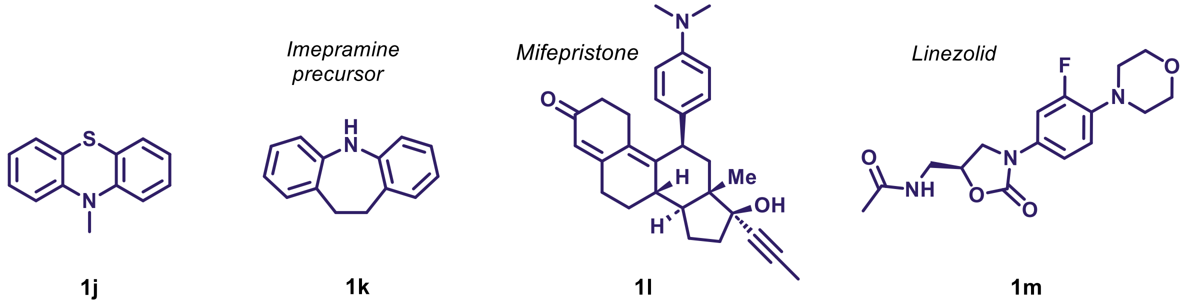

1j 1k 1l 1m

Synthesis of *tert*-butyl 4-(4-methoxyphenyl)piperazine-1-carboxylate (1g)

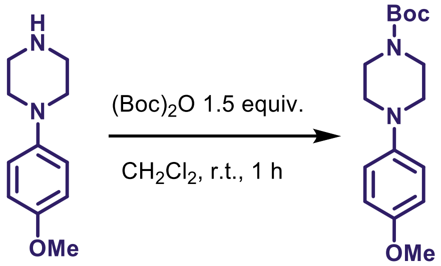

To a solution of 1-(4-methoxyphenyl)piperazine (192 mg, 1.0 mmol) in dry CH_2Cl_2 (5 mL), was added the BOC-anhydride (725 mg, 1.5 mmol) and the reaction mixture was stirred at room temperature for 1 h. Then, it was concentrated under reduced pressure and the residue was purified by flash column chromatography (hexane:EtOAc, 9:1) to afford **1g** as a white solid (290 mg, 99% yield).

^{1}H NMR (300 MHz, $CDCl_3$): δ 6.93 – 6.78 (m, 4H), 3.75 (s, 3H), 3.60 – 3.52 (m, 4H), 3.07 – 2.93 (m, 4H), 1.47 (s, 9H).

^{13}C NMR (75.5 MHz, $CDCl_3$) δ 154.8, 154.3, 145.8, 118.9, 114.6, 79.9, 55.6, 51.0, 43.8, 28.5. **HRMS** (APCI, m/z): calculated for $C_{16}H_{25}N_2O_3$ [M^+ + H]: 293.1860; found: 293.1853.

The spectroscopic signals are in accordance with the previous literature.[8]

b) Sulfinates **2**

Non-commercially available sulfinates **2c**,[4] **2e**,[5] **2f**,[6] **2g**[7] were synthesized according to literature procedures.

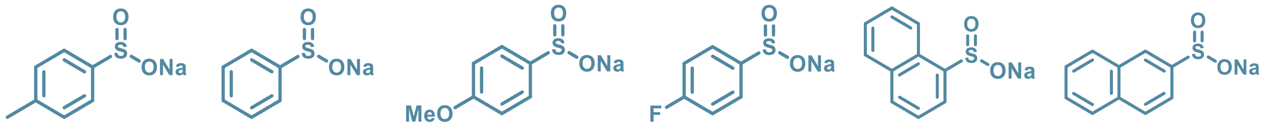

2a **2b** **2c** **2d** **2e** **2f**

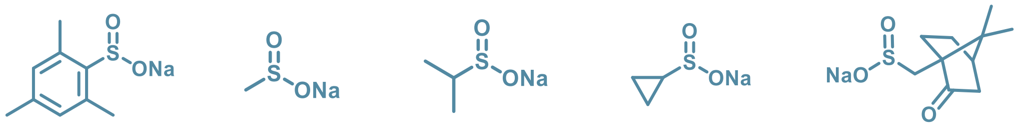

2g **2h** **2i** **2j** **2k**

Synthesis of sodium [(1*S*)-7,7-dimethyl-2-oxobicyclo[2.2.1]heptan-1-yl]methanesulfinate (2k)

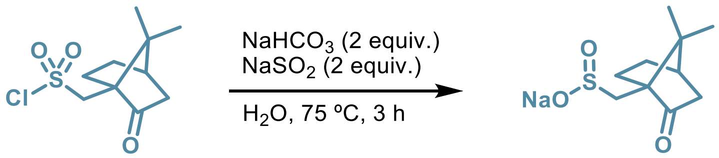

To a solution of sodium bicarbonate (0.84 g, 10 mmol) and sodium sulfite (1.26 g, 10 mmol) in H_2O (10 mL), was added the (1*S*)-(+)-10-camphorsulfonyl chloride (1.25 g, 5 mmol) and the reaction mixture was stirred at 0 ºC for 30 min., after this time, it was heated to 75 ºC for 3h. After cooling down to room temperature, H_2O was removed in vacuo. Methanol (25 mL) was then added to this white residue and the resulting heterogeneous solution was filtered. The filtrate was concentrated under reduced pressure and co-evaporated with Et_2O to afford **2k** as white powder (1.05 g, 88% yield).

^{1}H NMR (300 MHz, $CDCl_3$): δ 3.20 (s, 2H), 2.58 (d, *J* = 13.6 Hz, 1H), 2.31 (d, *J* = 13.6 Hz, 1H), 2.21 – 2.06 (m, 1H), 2.02 – 1.83 (m, 2H), 1.80 – 1.66 (m, 1H), 1.43 – 1.25 (m, 1H), 0.95 (s, 3H), 0.81 (s, 3H).

^{13}C NMR (75.5 MHz, $CDCl_3$) δ 219.8, 59.3, 59.0, 48.5, 43.3, 42.7, 27.5, 26.2, 20.3, 19.7.

HRMS (APCI, m/z): calculated for $C_{10}H_{15}O_3S$ [M^-]: 215.0747; found: 215.0749.

Specific rotation: $[\alpha]_D^{20}$ + 42.7 (*c*=1.0, $CHCl_3$).

4. Optimization studies

a) Initial studies

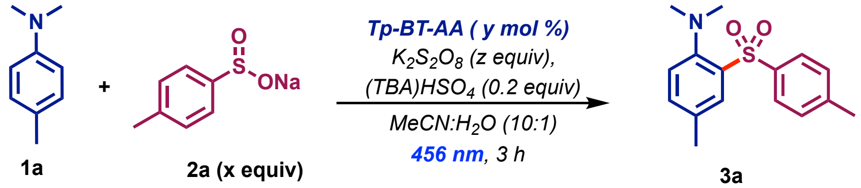

Entry	Tp-BT-AA (mol%)	2a (equiv)	$K_2S_2O_8$ (equiv)	3a yield (%)[b]
**1**	0.14	5	3	87
2	0.14	3	3	88
3	0.14	3	2	75
4	0.14	2	3	73
5	***0.07***	***3***	***3***	***89***
6	***0.035***	***3***	***3***	***88***

[a] Reaction conditions: **1a** (0.4 mmol), **2a** (x equiv), **Tp-BT-AA** (y mol%), $K_2S_2O_8$ (z equiv), (TBA)HSO_4 (0.2 equiv) in MeCN/H_2O 10:1 (4 mL), stirred at room temperature, under air atmosphere and irradiated with blue light 456 nm Kessil lamp for 3 h. [b] Yield of isolated product.

b) Screening of light sources

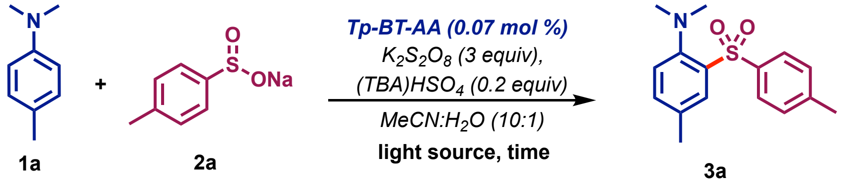

Entry	Light	Time (h)	3a yield (%)[b]
1	White CFL	24	44
2	456 nm	3	89
3	***525 nm***	***2***	***95***
4	595 nm	6	87
5	660 nm	30	57
6	***2 x 660 nm***	***12***	***85***
7	740 nm	48	35

[a]Reaction conditions: **1a** (0.4 mmol), **2a** (3 equiv), **Tp-BT-AA** (0.07 mol%), $K_2S_2O_8$ (3 equiv), (TBA)HSO_4 (0.2 equiv) in MeCN/H_2O 10:1 (4 mL), stirred at room temperature, under air atmosphere and irradiated at the indicated wavelength. [b] Yield of isolated product.

c) Comparison with other COFs

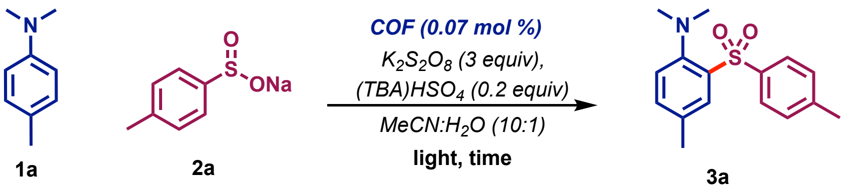

Entry	Variation	3a yield (%)[b]
1	**Tp-BT-AA**, **525nm**, 5 h (**660nm**, 20 h)	95 (85)
2	**Tp-BT-AA**,[c] **525nm**, 5 h (**660nm**, 20 h)	91 (82)
3	**Tp-BT-AB**, **525nm**, 5 h (**660nm**, 20 h)	19 (traces)
4	**Tp-BT-ABC**, **525nm**, 5 h (**660nm**, 20 h)	traces (traces)
5	**Tp-TD-AA**, **525nm**, 2 h	93
6	**Tp-TD-AA**, **595nm**, 6 h	23
7	**Tp-TD-AA**, **660nm**, 12 h	traces
8	**Tp** as photocatalyst	traces
9	**BT** as photocatalyst	traces

[a] Reaction conditions: **1a** (0.4 mmol), **2a** (3 equiv), **COF** (0.07 mol%), $K_2S_2O_8$ (3 equiv), (TBA)HSO_4 (0.2 equiv) in MeCN/H_2O 10:1 (4 mL), stirred at room temperature, under air atmosphere and irradiated with at the indicated wavelength. [b] Yield of isolated product. [c] **Tp-BT-AA** COF synthesized using acetic acid as the modulator instead of pyrrolidine. **TP** = 4,4''-diamino-*p*-terphenyl, **BT** = 4,7-bis(4-aminophenyl)-2,1,3-benzothiadiazole.

d) Control studies

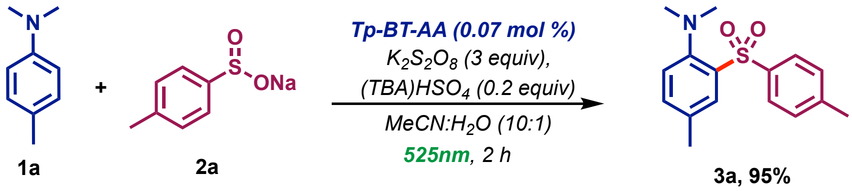

Entry	Variation	3a yield (%)[b]
1	*none*	*95*
2	O_2 instead of $K_2S_2O_8$	traces
3	MeCN as solvent	traces
4	H_2O as solvent	8
5	No **Tp-BT-AA** COF	traces
6	Reaction in dark	traces
9	No (TBA)HSO_4	65

[a] Reaction conditions: **1a** (0.4 mmol), **2a** (3 equiv), **Tp-BT-AA** (0.07 mol%), $K_2S_2O_8$ (3 equiv), (TBA)HSO_4 (0.2 equiv) in MeCN/H_2O 10:1 (4 mL), stirred at room temperature, under air atmosphere and irradiated with a 525 nm Kessil lamp. [b] Yield of isolated product.

5. General procedure for the photocatalytic sulfonylation of anilines (GP-1)

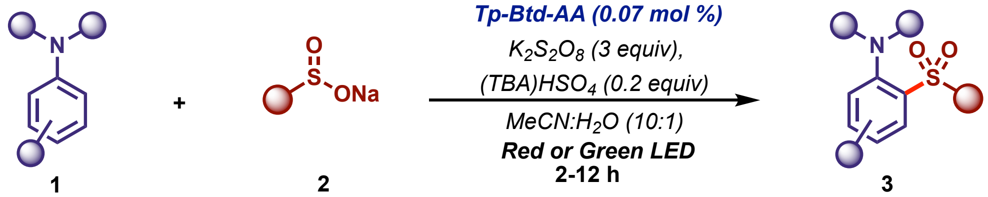

A 10 ml screw cap glass vial was charged with **Tp-BT-AA** COF (0.8 mg, 0.07 mol%), sodium sulfinate **2** (3 equiv, 1.2 mmol), tetrabutylammonium hydrogenosulfate (28 mg, 20 mol%), potassium persulfate (324 mg, 1.2 mmol) and aniline **1** (1 eq., 0.4 mmol) followed by 4 mL of a mixture of MeCN/H_2O 10:1. The resulting mixture was sonicated for 2 minutes, after which it was stirred under irradiation using either a 525 nm (green) Kessil lamp or two 660 nm (red) Kessil lamps. Ambient reaction temperature was maintained using a fan. Once the starting material **1** was consumed (checked by TLC), the mixture was diluted with EtOAc (4 mL) and H_2O (2 mL) followed by extraction with EtOAc (3 x 5mL). The combined organic layers were transferred to a centrifuge tube and centrifuged at 6500 rpm for 3 minutes and the liquid phase was separated. To the residue was added MeOH (8 mL), mixed thoroughly, centrifuged at 6500 rpm for 3 minutes, and the liquid phase was separated. The same procedure was repeated with 8 mL of MeCN. The combined liquid phases were concentrated under reduced pressure, and the crude product was purified by flash chromatography on a FlashPure cartridge (EcoFlex Silica 12 g) using a gradient solvent system (hexane:EtOAc) to afford product **3**.

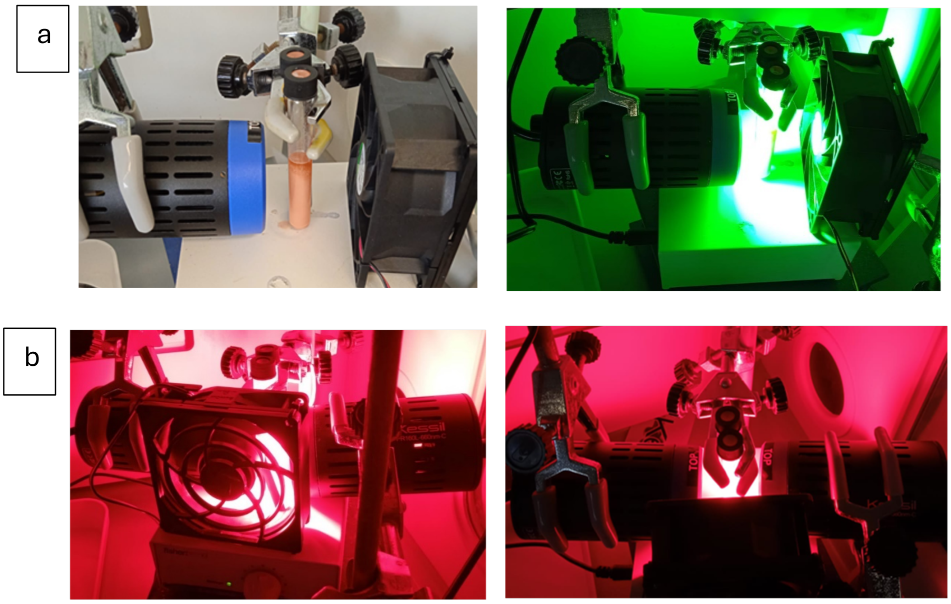

Figure S12. Set-up for the photocatalytic reactions at 0.4 mmol scale: a) Performed with green light (525 nm). b) Performed with red light (660 nm).

6. Large scale reaction

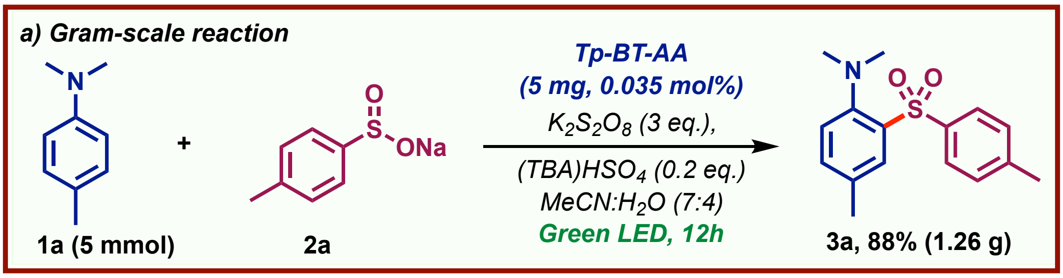

To a 100 mL schlenk tube charged with **Tp-BT-AA** (5 mg, 0.0175 mmol, 0.035 mol%), sodium sulfinate **2a** (2.67 g, 15 mmol), tetrabutylammonium hydrogensulfate (510 mg, 1.5 mmol), potassium persulfate (4.05 g, 15 mmol) and aniline **1** (730 µL 5 mmol) was added 69 mL of a mixture of MeCN/H_2O (7:4). The resulting mixture was sonicated for 2 minutes, after which it was stirred under irradiation by 2 x 44W 525 nm (green) Kessil lamps. Ambient reaction temperature was maintained using two fans (**Figure S11**). The reaction was monitored using TLC, once the starting material **1a** was consumed (12h), the mixture was diluted with EtOAc (50 mL) and H_2O (50 mL) followed by extraction with EtOAc (3 x 50 mL). The combined organic layers were concentrated under reduced pressure to 20 ml which was transferred to a centrifuge tube and centrifuged at 6500 rpm for 3 minutes and the liquid phase was separated. To the solid residue was added MeOH (20 mL), mixed thoroughly, centrifuged at 6500 rpm for 3 minutes, and the liquid phase was separated. The same procedure was repeated with 20 mL of MeCN. The combined liquid phases were concentrated under reduced pressure, and the crude product was purified by Flash chromatography on a FlashPure cartridge (EcoFlex Silica 40 g) using a gradient solvent system (hexane:EtOAc; 99:1 to 60:40) to afford product **3a** as a white solid (88% yield; 1.27 g).

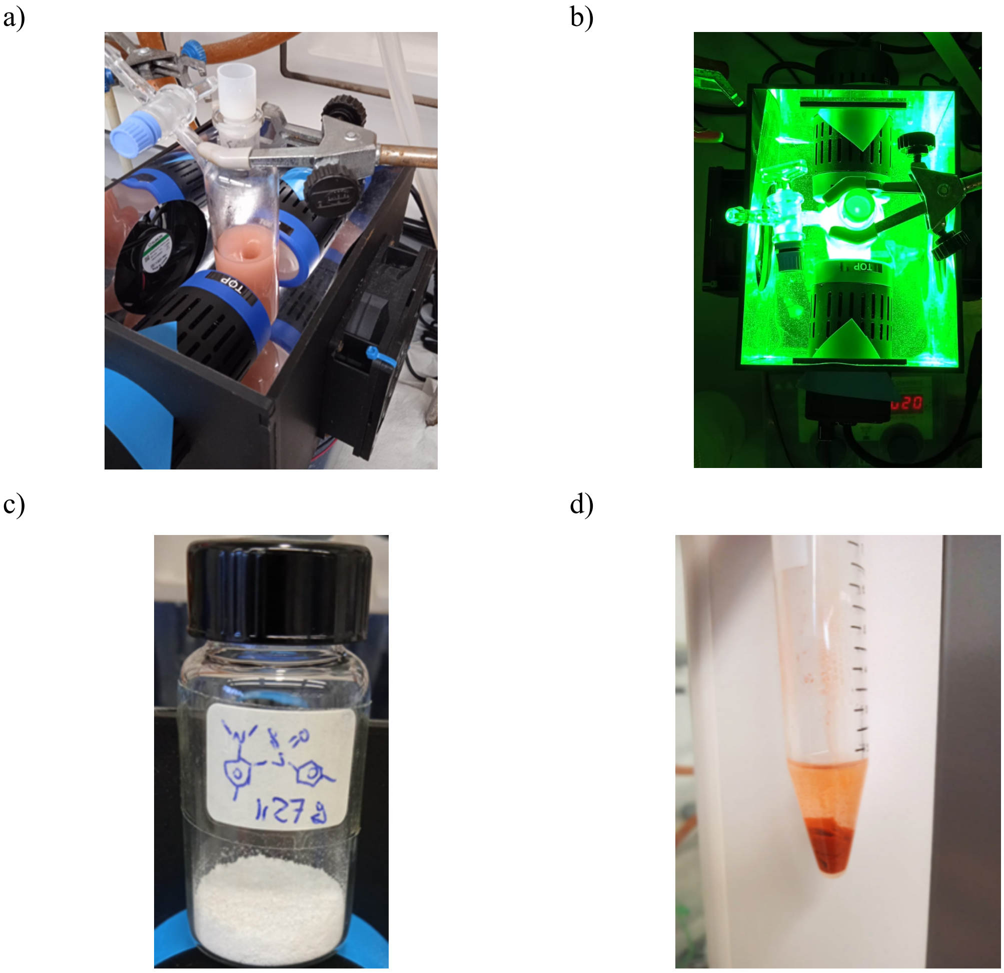

Figure S13. Large scale reaction set-up, a) side and b) top views. c) Isolated product. d) Recovered **Tp-BT-AA** COF.

7. Photocatalyst recyclability studies

The photocatalytic reaction was performed according to the GP-1 using **Tp-BT-AA** COF (10 mg, 0.035 mmol, 0.07 mol%), sodium sulfinate **2a** (2.67 g, 15 mmol), tetrabutylammonium hydrogensulfate (510 mg, 1.5 mmol), potassium persulfate (4.05 g, 15 mmol), aniline **1a** (730 μL 5 mmol) and CH_3CN/H_2O 7:4 (69 mL). On completion of the reaction, the COF was recovered by centrifugation and washed with MeOH (20 mL), CH_3CN (20 mL), H_2O (20 mL), THF (20 mL), and acetone (20 mL), dried at room temperature under vacuum overnight, and used for the next cycle. **Tp-BT-AA** was used recovered and reused for six consecutive cycles and after each cycle, product **3a** was isolated and purified by flash chromatography on a FlashPure cartridge (EcoFlex Silica 40 g) using a gradient solvent system (hexane:EtOAc; 99:1 to 60:40).

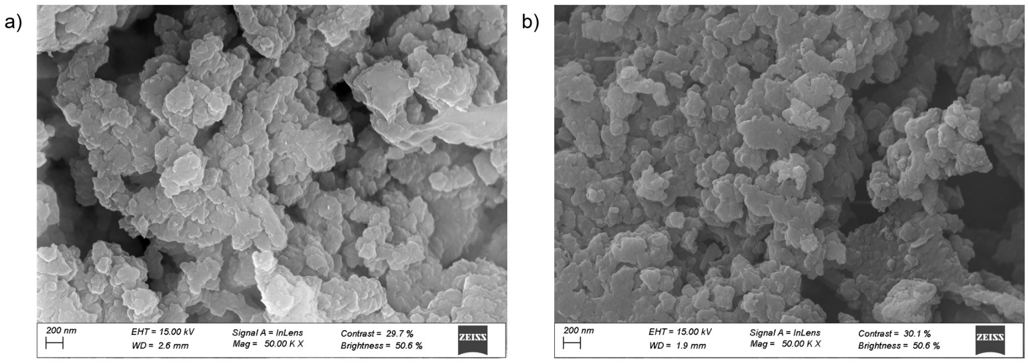

Figure S14. SEM images of **Tp-BT-AA** COF a) before and b) after six consecutive catalytic cycles.

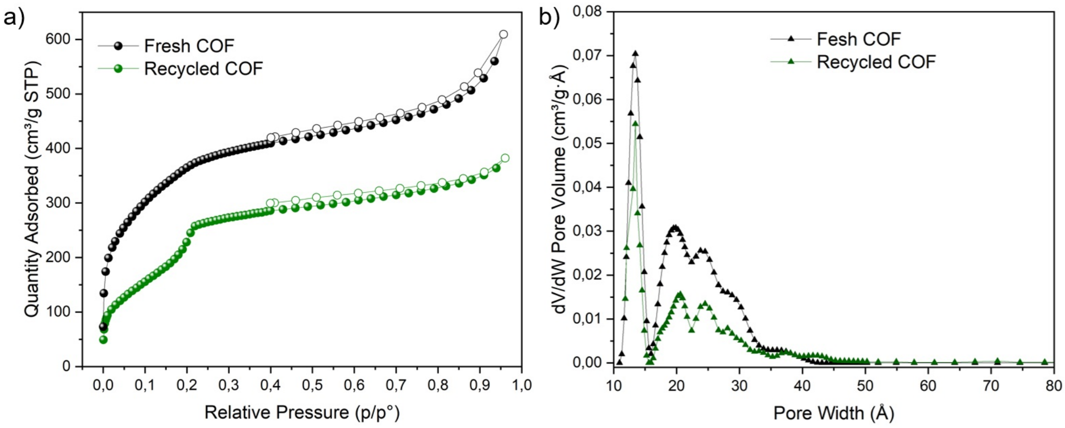

Figure S15. a) N_2 adsorption (filled symbols) and desorption (empty symbols) isotherms, and b) pore size distribution of **Tp-BT-AA** before and after six catalytic cycles.

8. Control experiments

Light on/light off experiment

Five identical reactions were setup following the GP-1 using sodium sulfinate **2a** (214 mg, 1.2 mmol) and aniline **1a** (59 µL, 0.4 mmol), in green light. The light was switched on and off every 40 minutes and one reaction was removed at each time point, and the isolated yields were obtained and plotted with time to obtain the graph in **Figure S13**.

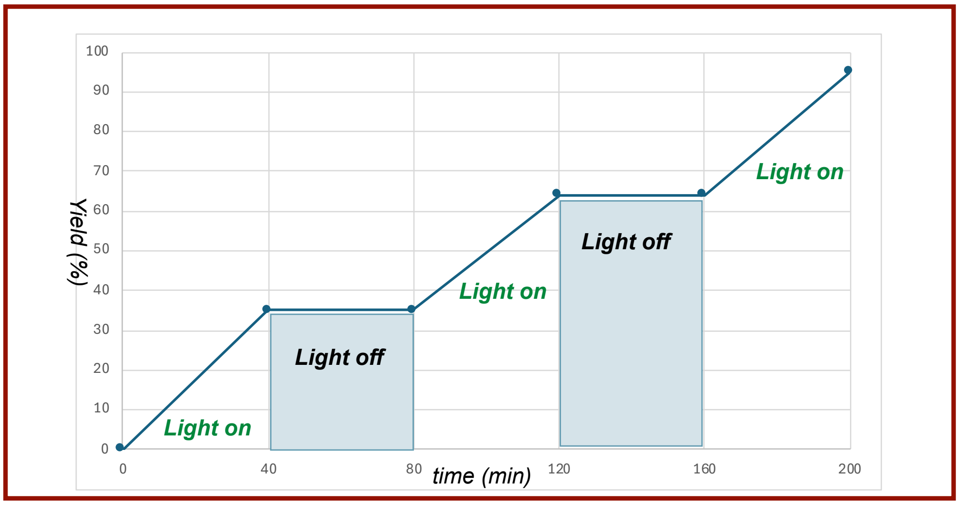

Figure S16. Light on/light off experiment

9. Compound characterization

N,*N*,4-Trimethyl-2-tosylaniline (3a)

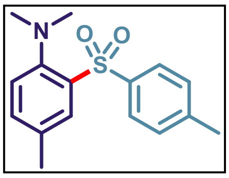

Synthesized from aniline **1a** (59 µL, 0.4 mmol) and sulfinate **2a** (214 mg, 1.2 mmol) following the GP-1. Isolated after flash column chromatography (hexane:EtOAc, 100:0 to 70:30) as a white solid; 95% yield (110 mg, green light, 2 h) and 85% (98 mg, red light, 12 h).

^{1}H NMR (300 MHz, $CDCl_3$): δ 8.06 (s, 1H), 7.79 (d, *J* = 8.1 Hz, 2H), 7.36 (d, *J* = 8.0 Hz, 1H), 7.24 (d, *J* = 8.1 Hz, 2H), 7.17 (d, *J* = 8.0 Hz, 1H), 2.42 (s, 3H), 2.41 (s, 3H), 2.39 (s, 6H).

^{13}C NMR (75.5 MHz, $CDCl_3$) δ 151.2, 143.1, 139.5, 137.9, 135.3, 135.2, 129.7, 128.6, 128.2, 124.3, 45.4, 21.6, 20.9.

The spectroscopic signals are in accordance with the previous literature.[9]

N,*N*,4-Trimethyl-2-(phenylsulfonyl)aniline (3b)

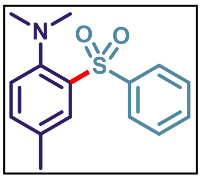

Synthesized from aniline **1a** (59 µL, 0.4 mmol) and sulfinate **2b** (197 mg, 1.2 mmol) following the GP-1. Isolated after flash column chromatography (hexane:EtOAc, 100:0 to 70:30) as a white solid; 94% yield (103 mg, green light, 2 h) and 89% (98 mg, red light, 12 h).

^{1}H NMR (300 MHz, $CDCl_3$): δ 8.04 (s, 1H), 7.87 (d, *J* = 8.0, Hz, 2H), 7.54 – 7.46 (m, 1H), 7.45 – 7.38 (m, 2H), 7.38 – 7.30 (m, 1H), 7.14 (d, *J* = 8.0, Hz, 1H), 2.40 (s, 3H), 2.33 (s, 6H). [1]

^{3}C NMR (75.5 MHz, $CDCl_3$) δ 151.3, 142.6, 137.8, 135.5, 135.4, 132.4, 129.8, 128.1, 128.1, 124.4, 45.3, 21.0.

The spectroscopic signals are in accordance with the previous literature.[10]

2-[(4-Methoxyphenyl)sulfonyl]-*N*,*N*,4-trimethylaniline (3c)

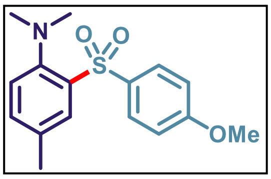

Synthesized from aniline **1a** (59 µL, 0.4 mmol) and sulfinate **2c** (231 mg, 1.2 mmol) following the GP-1. Isolated after flash column chromatography (hexane:EtOAc, 100:0 to 60:40) as a white solid; 60% yield (73 mg, green light, 5 h) and 78% (95 mg, red light, 16 h).

^{1}H NMR (300 MHz, $CDCl_3$): δ 8.02 (s, 1H), 7.83 (d, *J* = 8.9 Hz, 2H), 7.32 (d, *J* = 8.0 Hz, 1H), 7.14 (d, *J* = 8.0 Hz, 1H), 6.89 (d, *J* = 8.9 Hz, 2H), 3.82 (s, 3H), 2.39 (s, 9H).

^{13}C NMR (75.5 MHz, $CDCl_3$) δ 162.9, 151.2, 138.3, 135.3, 135.2, 134.2, 130.6, 129.7, 124.3, 113.3, 55.6, 45.6, 21.0.

The spectroscopic signals are in accordance with the previous literature.[9]

2-[(4-Fluorophenyl)sulfonyl]-N,N,4-trimethylaniline (3d)

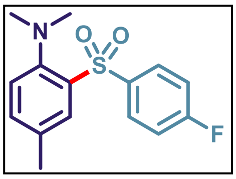

Synthesized from aniline **1a** (59 µL, 0.4 mmol) and sulfinate **2d** (218 mg, 1.2 mmol) following the GP-1. Isolated after flash column chromatography (hexane:EtOAc, 100:0 to 70:30) as a white solid; 89% yield (104 mg, green light, 2 h) and 86% (101 mg, red light, 12 h).

^{1}H NMR (300 MHz, $CDCl_3$): δ 8.04 (s, 1H), 7.96 – 7.86 (m, 2H), 7.36 (d, *J* = 8.5 Hz, 1H), 7.16 (d, *J* = 8.4 Hz, 1H), 7.13 – 7.05(m, 2H), 2.41 (s, 3H), 2.38 (s, 6H).

^{13}C NMR (75.5 MHz, $CDCl_3$): δ 165.0 (d, *J* = 254.2 Hz), 151.1, 138.4, 137.5, 135.5, 135.4, 130.9 (d, *J* = 9.3 Hz), 129.7, 124.2, 115.1 (d, *J* = 22.6 Hz), 45.4, 20.9.

^{19}F NMR (282 MHz, $CDCl_3$): δ –105.95 (tt, *J* = 8.4, 4.2 Hz, 1F).

The spectroscopic signals are in accordance with the previous literature.[11]

N,N,4-Trimethyl-2-(naphthalen-1-ylsulfonyl)aniline (3e)

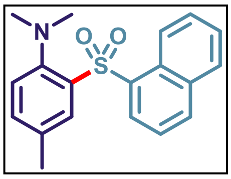

Synthesized from aniline **1a** (59 µL, 0.4 mmol) and sulfinate **2e** (257 mg, 1.2 mmol) following the GP-1. Isolated after flash column chromatography (hexane:EtOAc, 100:0 to 70:30) as a white solid; 37% (48 mg, blue light, 6 h), 54% yield (70 mg, green light, 5 h) and 49% (64 mg, red light, 16 h).

^{1}H NMR (300 MHz, $CDCl_3$): δ 8.50 (d, *J* = 7.4 Hz, 1H), 8.28 (d, *J* = 7.5 Hz, 1H), 8.24 (s, 1H), 8.02 (d, *J* = 8.1 Hz, 1H), 7.92 – 7.81 (m, 1H), 7.60 (t, *J* = 7.8 Hz, 1H), 7.51 – 7.38 (m, 2H), 7.32 (d, *J* = 8.1 Hz, 1H), 7.06 (d, *J* = 8.1 Hz, 1H), 2.46 (s, 3H), 2.10 (s, 6H).

^{13}C NMR (126 MHz, $CDCl_3$): δ 151.1, 138.6, 137.4, 135.6, 135.4, 133.9, 133.9, 130.2, 129.4, 128.8, 128.5, 127.8, 126.3, 124.5, 124.3, 124.1, 44.9, 21.2.

The spectroscopic signals are in accordance with the previous literature.[12]

N,N,4-Trimethyl-2-(naphthalen-2-ylsulfonyl)aniline (3f)

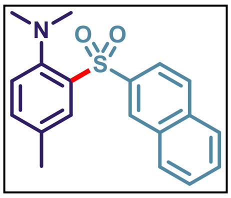

Synthesized from aniline **1a** (59 µL, 0.4 mmol) and sulfinate **2f** (257 mg, 1.2 mmol) following the GP-1. Isolated after flash column chromatography (hexane:EtOAc, 100:0 to 70:30) as a white solid; 39% (51 mg, blue light, 6 h), 62% yield (80 mg, green light, 2 h) and 74% (96 mg, red light, 16 h) and

^{1}H NMR (300 MHz, $CDCl_3$): δ 8.49 (s, 1H), 8.12 (s, 1H), 8.00 – 7.80 (m, 4H), 7.66 – 7.49 (m, 2H), 7.35 (d, *J* = 7.4 Hz, 1H), 7.13 (d, *J* = 7.6 Hz, 1H), 2.43 (s, 3H), 2.32 (s, 5H).

^{13}C NMR (75.5 MHz, $CDCl_3$): δ 151.3, 139.7, 138.0, 135.6, 135.5, 134.9, 132.1, 130.0, 129.5, 129.4, 128.7, 128.0, 127.9, 127.2, 124.4, 123.8, 45.5, 21.1.

The spectroscopic signals are in accordance with the previous literature.[11]

2-(Mesitylsulfonyl)-N,N,4-trimethylaniline (3g)

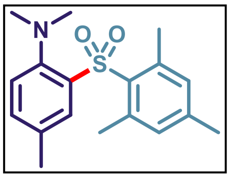

Synthesized from aniline derivative **1a** (59 µL, 0.4 mmol) and sulfinate **2g** (247 mg, 1.2 mmol) following the GP-1. Isolated after flash column chromatography (hexane:EtOAc, 100:0 to 70:30) as a

white solid; 29% yield (37 mg, green light, 5 h) and 28% (35 mg, red light, 16 h).

^{1}H NMR (300 MHz, $CDCl_3$): δ 7.99 (s, 1H), 7.35 (d, *J* = 8.2 Hz, 1H), 7.22 (d, *J* = 8.2 Hz, 1H), 6.85 (s, 2H), 2.48 (s, 6H), 2.41 (s, 3H), 2.25 (s, 3H), 2.22 (s, 6H).

^{13}C NMR (75.5 MHz, $CDCl_3$): δ ^{13}C NMR (75 MHz, $cdcl_3$) δ 151.1, 141.6, 140.4, 139.3, 136.7, 135.6, 134.8, 131.37, 129.1, 124.8, 45.4, 22.1, 21.0, 20.9.

HRMS (APCI, m/z): calculated for $C_{18}H_{24}NO_2S$ [M^+ + H]: 318,1522; found: 318.1519.

***N*,*N*,4-Trimethyl-2-(methylsulfonyl)aniline (3h)**

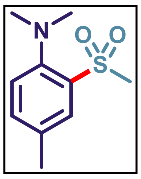

Synthesized from aniline derivative **1a** (59 µL, 0.4 mmol) and sulfinate **2h** (122 mg, 1.2 mmol) following the GP-1. Isolated after flash column chromatography (hexane:EtOAc, 100:0 to 60:40) as a white solid; 86% yield (73 mg, green light, 2 h) and 80% (68 mg, red light, 12 h).

^{1}H NMR (300 MHz, $CDCl_3$): δ 7.85 (d, *J* = 1.0 Hz, 1H), 7.39 (dd, *J* = 8.1, 1.0 Hz, 1H), 7.31 (d, *J* = 8.1 Hz, 1H), 3.30 (s, 3H), 2.75 (s, 6H), 2.37 (s, 3H).

^{13}C NMR (75.5 MHz, $CDCl_3$) δ 151.4, 136.4, 135.3, 135.2, 129.6, 123.4, 46.2, 42.6, 20.8.

The spectroscopic signals are in accordance with the previous literature.[9]

2-(Isopropylsulfonyl)-*N*,*N*,4-trimethylaniline (3i)

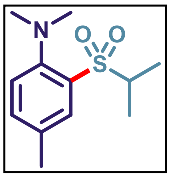

Synthesized from aniline derivative **1a** (59 µL, 0.4 mmol) and sulfinate **2i** (156 mg, 1.2 mmol) following the GP-1. Isolated after flash column chromatography (hexane:EtOAc, 100:0 to 70:30) as a white solid; 78% yield (75 mg, green light, 2 h) and 62% (60 mg, red light, 12 h).

^{1}H NMR (300 MHz, $CDCl_3$): δ 7.83 (s, 1H), 7.37 (d, *J* = 8.1 Hz, 1H), 7.29 (d, *J* = 8.1 Hz, 1H) 4.14 (hept, *J* = 6.9 Hz, 1H), 2.72 (s, 6H), 2.37 (s, 3H), 1.23 (d, *J* = 6.9 Hz, 6H).

^{13}C NMR (75.5 MHz, $CDCl_3$) δ 151.8, 135.4, 135.1, 133.8, 131.2, 123.3, 52.7, 46.4, 20.9, 15.3.

The spectroscopic signals are in accordance with the previous literature.[9]

N,N,4-Trimethyl-2-(methylsulfonyl)aniline (3j)

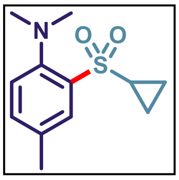

Synthesized from aniline derivative **1a** (59 µL, 0.4 mmol) and sulfinate **2j** (154 mg, 1.2 mmol) following the GP-1. Isolated after flash column chromatography (hexane:EtOAc, 100:0 to 70:30) as a white solid; 94% yield (90 mg, green light, 2 h) and 86% (82 mg, red light, 12 h).

^{1}H NMR (300 MHz, $CDCl_3$): δ 7.71 (s, 1H), 7.36 (m, 2H), 3.49 – 3.29 (m, 1H), 2.75 (s, 6H), 2.36 (s, 3H), 1.31 – 1.17 (m, 2H), 1.01 – 0.88 (m, 2H).

^{13}C NMR (75.5 MHz, $CDCl_3$) δ 151.6, 136.9, 135.3, 134.9, 129.3, 123.8, 46.3, 31.4, 20.9, 5.4.

The spectroscopic signals are in accordance with the previous literature.[9]

4-Methoxy-N,N-dimethyl-2-tosylaniline (3k)

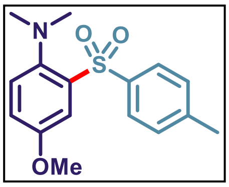

Synthesized from aniline derivative **1b** (61 mg, 0.4 mmol) and sulfinate **2a** (214 mg, 1.2 mmol) following the GP-1. Isolated after flash column chromatography (hexane:EtOAc, 100:0 to 50:50) as a white solid; 78% yield (95 mg, green light, 3 h) and 87% (106 mg, red light, 14 h).

^{1}H NMR (300 MHz, $CDCl_3$): δ 7.78 (d, J = 8.5 Hz, 2H), 7.75 (d, J = 2.8 Hz, 1H), 7.23 (d, J = 8.5 Hz, 2H), 7.20 (d, J = 8.7 Hz, 1H), 7.08 (dd, J = 8.7, 2.8 Hz, 1H), 3.87 (s, 3H), 2.39 (s, 3H), 2.31 (s, 6H).

^{13}C NMR (75.5 MHz, $CDCl_3$) δ 156.9, 146.5, 143.3, 139.51, 139.47, 128.8, 128.4, 125.4, 121.4, 113.2, 56.0, 45.5, 21.7.

The spectroscopic signals are in accordance with the previous literature.[13]

4-Chloro-N,N-dimethyl-2-tosylaniline (3l)

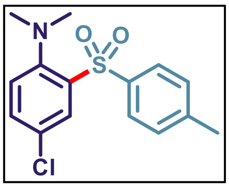

Synthesized from aniline derivative **1c** (63 mg, 0.4 mmol) and sulfinate **2a** (214 mg, 1.2 mmol) following the GP-1. Isolated after flash column chromatography (hexane:EtOAc, 100:0 to 70:30) as a white solid; 32% yield (40 mg, green light, 5 h) and 36% (45 mg, red light, 16 h).

^{1}H NMR (300 MHz, $CDCl_3$): δ 8.22 (d, J = 2.5 Hz, 1H), 7.77 (d, J = 8.6 Hz, 2H), 7.49 (dd, J = 8.5, 2.5 Hz, 1H), 7.25 (d, J = 8.6 Hz, 2H), 7.18 (d, J = 8.5 Hz, 1H), 2.41 (s, 6H), 2.40 (s, 3H).

^{13}C NMR (75.5 MHz, $CDCl_3$) δ 152.4, 143.8, 139.8, 138.8, 134.7, 130.7, 129.8, 128.9, 128.5, 125.9, 45.5, 21.7.

The spectroscopic signals are in accordance with the previous literature.[9]

_N,N_-Dimethyl-2-tosylaniline (3m)

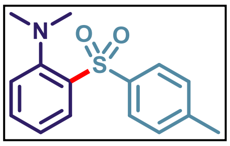

Synthesized from aniline derivative **1d** (51 μL, 0.4 mmol) and sulfinate **2a** (214 mg, 1.2 mmol) following the GP-1. Isolated after flash column chromatography (hexane:EtOAc, 100:0 to 50:50) as a white solid; 38% (42 mg, green light, 3 h) and 32% (35 mg, red light, 16 h).

^{1}H NMR (300 MHz, $CDCl_3$): δ 8.23 (dd, J = 8.0, 1.4 Hz, 1H), 7.77 (d, J = 8.3 Hz, 2H), 7.54 (dt, J = 8.0, 1.4 Hz, 1H), 7.35 – 7.28 (m, 1H), 7.25 – 7.18 (m, 3H), 2.42 (s, 6H), 2.39 (s, 3H).

^{13}C NMR (126 MHz, $CDCl_3$) δ 154.0, 143.3, 139.6, 138.4, 134.7, 129.9, 128.8, 128.4, 125.2, 124.4, 45.5, 21.7.

The spectroscopic signals are in accordance with the previous literature.[12]

_N,N_-Dimethyl-4-tosylaniline (3m´)

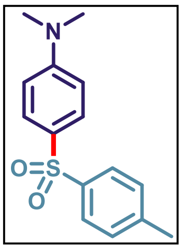

Synthesized from aniline derivative **1d** (51 μL, 0.4 mmol) and sulfinate **2a** (214 mg, 1.2 mmol) following the GP-1. Isolated after flash column chromatography (hexane:EtOAc, 100:0 to 60:40) as a white solid; in 18% yield (20 mg, green light, 3 h) and 15% (16 mg, red light, 16 h).

^{1}H NMR (300 MHz, $CDCl_3$): δ 7.81 – 7.68 (m, 4H), 7.24 (d, J = 9.5 Hz, 2H), 6.64 (d, J = 9.0 Hz, 2H), 3.01 (s, 6H), 2.37 (s, 3H).

^{13}C NMR (126 MHz, $CDCl_3$) δ 153.2, 143.1, 140.7, 129.8, 129.5, 127.2, 127.2, 111.2, 40.2, 21.6.

The spectroscopic signals are in accordance with the previous literature.[14]

Note: This compound was isolated with around 15% of the p-toluene sulfonic acid (2 mg)

***N,N,N',N'*-Tetramethyl-3-tosyl-(1,1'-biphenyl)-4,4'-diamine (3n)**

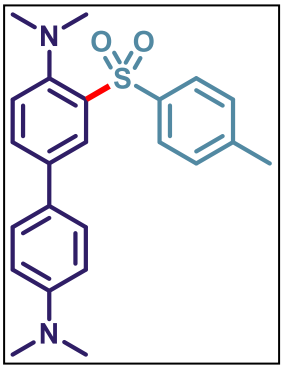

Synthesized from aniline derivative **1e** (96 mg, 0.4 mmol) and sulfinate **2a** (214 mg, 1.2 mmol) following the GP-1. Isolated after flash column chromatography (hexane:EtOAc, 100:0 to 50:50) as a white solid; 57% yield (90 mg, green light, 2 h) and 48% (76 mg, red light, 12 h).

Melting range: 156-158 °C

^{1}H NMR (300 MHz, $CDCl_3$): δ 8.43 (d, *J* = 2.2 Hz, 1H), 7.81 (d, *J* = 8.2 Hz, 2H), 7.71 (dd, *J* = 8.3, 2.2 Hz, 1H), 7.53 (d, *J* = 8.8 Hz, 2H), 7.30 – 7.19 (m, 3H), 6.81 (d, *J* = 8.8 Hz, 2H), 3.01 (s, 6H), 2.44 (s, 6H), 2.39 (s, 3H).

^{13}C NMR (75.5 MHz, $CDCl_3$): δ 151.5, 150.4, 143.2, 139.7, 138.5, 131.8, 128.8, 128.4, 127.8, 127.2, 127.1, 124.7, 112.9, 45.6, 40.6, 21.7.

HRMS (APCI, m/z): calculated for $C_{23}H_{27}N_2O_2S$ [M^+ + H]: 395.1788; found: 395.1787.

4-(4-Methyl-2-tosylphenyl)morpholine (3o)

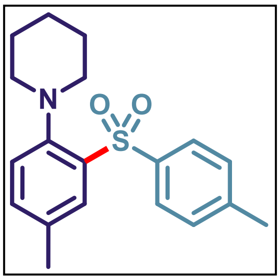

Synthesized from aniline derivative **1f** (70 mg, 0.4 mmol) and sulfinate **2a** (214 mg, 1.2 mmol) following the GP-1. Isolated after flash column chromatography (hexane:EtOAc, 100:0 to 50:50) as a yellow oil; 77% yield (101 mg, green light, 5 h) and 74% (97 mg, red light, 16 h).

^{1}H NMR (300 MHz, $CDCl_3$): δ 8.10 (s, 1H), 7.79 (d, *J* = 8.3 Hz, 2H), 7.37 (d, *J* = 8.0 Hz, 1H), 7.24 (d, *J* = 7.5 Hz, 2H), 7.15 (s, 1H), 2.63 (brs, 4H), 2.43 (s, 3H), 2.41 (s, 3H), 1.47 (brs, 6H).

^{13}C NMR (300 MHz, $CDCl_3$): δ 150.6, 141.9, 138.9, 135.5, 134.2, 133.2, 129.5, 127.7, 126.6, 123.1, 53.90, 24.6, 22.9, 20.5, 19.9.

HRMS (APCI, m/z): calculated for $C_{19}H_{24}NO_2S$ [M^+ + H]: 330.1522; found: 330.1526.

4-(4-Methyl-2-tosylphenyl)morpholine (3o)

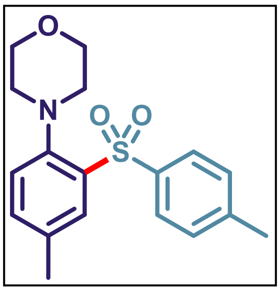

Synthesized from aniline derivative **1g** (71 mg, 0.4 mmol) and sulfinate **2a** (214 mg, 1.2 mmol) following the GP-1. Isolated after flash column chromatography (hexane:EtOAc, 100:0 to 50:50) as a white solid; 75% yield (99 mg, green light, 5 h) and 71% (94 mg, red light, 16 h).

Melting range: 161-164 °C

^{1}H NMR (300 MHz, $CDCl_3$): δ 8.11 (s, 1H), 7.77 (d, *J* = 8.8 Hz, 2H), 7.40 (d, *J* = 7.7 Hz, 1H), 7.24 (d, *J* = 8.8 Hz, 2H), 7.18 (d, *J* = 7.7 Hz, 1H), 3.65 – 3.53 (m, 4H), 2.75 – 2.62 (m, 4H), 2.43 (s, 3H), 2.39 (s, 3H).

^{13}C NMR (300 MHz, $CDCl_3$): δ 149.9, 143.3, 140.1, 137.3, 136.0, 135.6, 130.8, 129.0, 127.7, 124.4, 66.8, 53.7, 21.7, 21.1.

HRMS (APCI, m/z): calculated for $C_{18}H_{22}NO_3S$ [M^+ + H]: 332.1315; found: 332.1320.

***tert*-Butyl 4-(4-methoxy-2-tosylphenyl)piperazine-1-carboxylate (3p)**

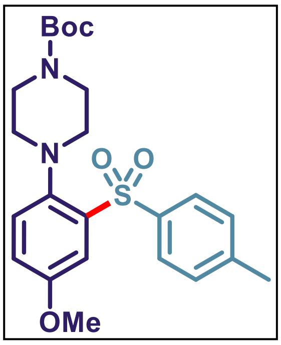

Synthesized from aniline derivative **1h** (117 mg, 0.4 mmol) and sulfinate **2a** (214 mg, 1.2 mmol) following the GP-1. Isolated after flash column chromatography (hexane:EtOAc, 100:0 to 50:50) as a white solid; 87% yield (156 mg, green light, 2 h) and 81% (145 mg, red light, 12 h).

Melting range: 78-82 °C

^{1}H NMR (300 MHz, $CDCl_3$): δ 7.82 (d, *J* = 2.5 Hz, 1H), 7.74 (d, *J* = 7.9 Hz, 2H), 7.25 (d, *J* = 8.0 Hz, 2H), 7.17 (d, *J* = 8.9 Hz, 1H), 7.11 (dd, *J* = 8.9, 2.5 Hz, 1H), 3.89 (s, 3H), 3.29 (br s, 4H), 2.59 (br s, 4H), 1.47 (s, 9H).

^{13}C NMR (75.5 MHz, $CDCl_3$): δ 157.2, 154.9, 145.1, 143.5, 139.9, 138.4, 129.1, 127.6, 125.6, 121.3, 114.4, 79.9, 56.1, 53.4, 43.7, 28.6, 21.7.δ.

HRMS (APCI, m/z): calculated for $C_{23}H_{31}N_2O_5S$ [M^+ + H]: 447,1948; found: 447,1950

_N,N_-Diphenyl-4-tosylaniline (3q)

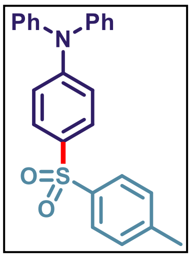

Synthesized from aniline derivative **1i** (98 mg, 0.4 mmol) and sulfinate **2a** (214 mg, 1.2 mmol) following the GP-1. Isolated after flash column chromatography (hexane:EtOAc, 100:0 to 70:30) as a white solid; 71% yield (113 mg, green light, 2 h) and 66% (105 mg, red light, 12 h).

^{1}H NMR (300 MHz, $CDCl_3$): δ 7.81 (d, J = 8.2 Hz, 2H), 7.68 (d, J = 8.9 Hz, 2H), 7.36 – 7.21 (m, 6H), 7.19 – 7.02 (m, 6H), 6.96 (d, J = 8.9 Hz, 2H), 2.40 (s, 3H).

^{13}C NMR (75.5 MHz, $CDCl_3$): δ 152.2, 146.2, 143.7, 139.8, 132.4, 129.9, 129.0, 127.5, 126.3, 125.2, 119.7, 21.7.

The spectroscopic signals are in accordance with the previous literature.[15]

10-Methyl-3-tosyl-10_H_-phenothiazineamine (3r)

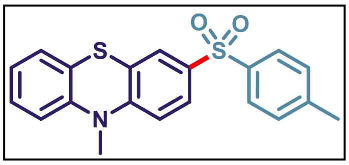

Synthesized from aniline derivative **1j** (85 mg, 0.4 mmol) and sulfinate **2** (214 mg, 1.2 mmol) following the GP-1. Isolated after flash column chromatography (hexane:EtOAc, 100:0 to 50:50) as a yellow oil; 84% yield (123 mg, green light, 2 h) and 62% (91 mg, red light, 12 h).

^{1}H NMR (300 MHz, $CDCl_3$): δ 7.78 (d, J = 8.2 Hz, 2H), 7.71 (dd, J = 8.5, 2.1 Hz, 1H), 7.60 (d, J = 2.1 Hz, 1H), 7.27 (d, J = 8.5 Hz, 2H), 7.21 – 7.13 (m, 1H), 7.10 (dd, J = 7.6, 1.4 Hz, 1H), 7.00 – 6.92 (m, 1H), 6.83 – 6.75 (m, 2H), 3.37 (s, 3H), 2.38 (s, 3H).

^{13}C NMR (75.5 MHz, $CDCl_3$): δ 150.1, 144.4, 144.0, 139.4, 135.3, 130.0, 128.0, 127.6, 127.5, 127.4, 126.3, 124.7, 123.8, 122.4, 114.9, 113.8, 35.8, 21.7.

HRMS (APCI, m/z): calculated for $C_{20}H_{18}NO_2S_2$ [M^+ + H]: 368.0773; found: 368.0787.

(1_S_)-1-{[(2-(Dimethylamino)-5-methylphenyl)sulfonyl]methyl}-7,7-dimethylbicyclo[2.2.1]heptan-2-one (3s)

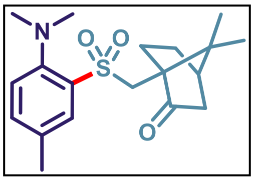

Synthesized from aniline derivative **1a** (59 µL, 0.4 mmol) and sulfinate **2k** (154 mg, 1.2 mmol) following the GP-1. Isolated after flash column chromatography (hexane:EtOAc, 100:0 to 60:40) as a

yellow oil; 82% yield (114 mg, green light, 5 h) and 84% (117 mg, red light, 16 h).

^{1}H NMR (300 MHz, $CDCl_3$): δ 7.84 (s, 1H), 7.38 (d, J = 8.1 Hz, 1H), 7.30 (d, J = 8.1 Hz, 1H), 4.12 (d, J = 14.8 Hz, 1H), 3.55 (d, J = 14.8 Hz, 1H), 2.77 (s, 6H), 2.61 – 2.52 (m, 1H), 2.37 (s, 3H), 2.36 – 2.31 (m, 1H), 2.11 – 1.99 (m, 2H), 1.89 (d, J = 18.4 Hz, 1H), 1.64 – 1.59 (m, 1H), 1.44 – 1.36 (m, 1H), 1.17 (s, 3H), 0.88 (s, 3H).

^{13}C NMR (126 MHz, $CDCl_3$): δ 215.0, 151.6, 137.1, 135.3, 135.2, 130.1, 123.5, 59.3, 50.3, 48.1, 46.5, 42.8, 42.6, 27.3, 24.8, 21.0, 20.4, 20.0. δ.

HRMS (APCI, m/z): calculated for $C_{19}H_{28}NO_3S$ [M^+ + H]: 350,1784; found: 350,1788.

Specific rotation: $[\alpha]_D^{20}$ + 3.5 (c=1.0, $CHCl_3$).

2-Tosyl-10,11-dihydro-5*H*-dibenzo[b,f]azepine (3t)

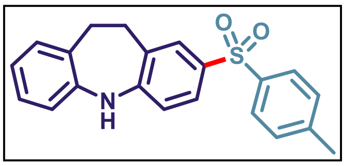

Synthesized from aniline derivative **1k** (80 mg, 0.4 mmol) and sulfinate **2a** (214 mg, 1.2 mmol) following the GP-1. Isolated after flash column chromatography (hexane:EtOAc, 100:0 to 50:50) as a pale-yellow solid; 79% yield (110 mg, green light, 2 h) and 70% yield (95 mg, red light, 12 h).

Melting range: 179-182 °C

^{1}H NMR (300 MHz, $CDCl_3$): δ 7.83 (d, J = 8.2 Hz, 2H), 7.50 – 7.55 (m, 2H), 7.30 (d, J = 8.2 Hz, 2H), 7.19 – 7.04 (m, 2H), 6.88 (t, J = 7.3 Hz, 1H), 6.79 (t, J = 9.2 Hz, 2H), 6.47 (br s, 1H), 3.17 – 2.98 (m, 4H), 2.41 (s, 3H).

^{13}C NMR (75.5 MHz, CDCl3): δ 146.6, 143.6, 140.8, 140.0, 136.2, 130.9, 130.6, 130.0, 129.9, 127.7, 127.4, 127.2, 126.9, 121.2, 118.8, 118.1, 35.6, 34.7, 21.6.

HRMS (APCI, m/z): calculated for $C_{21}H_{20}NO_2S$ [M^+ + H]: 350.1209; found: 350.1208.

2-(Cyclopropylsulfonyl)-10,11-dihydro-5*H*-dibenzo[b,f]azepine (3u)

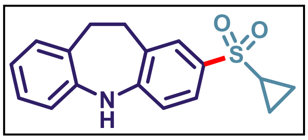

Synthesized from aniline derivative **1k** (80 mg, 0.4 mmol) and sulfinate **2j** (154 mg, 1.2 mmol) following the GP-1. Isolated after flash column chromatography (hexane:EtOAc, 100:0 to 40:60) as a pale- brown solid; 84% yield (100 mg, green light, 2 h) and 75% (90 mg, red light, 12 h).

Melting range: 153-156 °C

^{1}H NMR (300 MHz, $CDCl_3$): δ 7.61 – 7.50 (m, 2H), 7.17 – 7.04 (m, 2H), 6.93 – 6.77 (m, 3H), 6.52 (s, 1H), 3.17 – 3.00 (m, 4H), 2.53 – 2.35 (m, 1H), 1.39 – 1.25 (m, 2H), 1.13 – 0.91 (m, 2H). **^{13}C NMR** (75.5 MHz, $CDCl_3$): δ 146.9, 140.9, 130.9, 130.7, 129.9, 129.6, 127.7, 127.3, 126.8, 121.2, 118.9, 118.1, 35.6, 34.7, 33.5, 6.0.

HRMS (APCI, m/z): calculated for $C_{17}H_{18}NO_2S$ [M^+ + H]: 300,1053; found: 300.1047.

(8*S*,11*R*,13*S*,14*S*,17*S*)-11-(3-(Cyclopropylsulfonyl)-4-(dimethylamino)phenyl)-17-hydroxy-13-methyl-17-(prop-1-yn-1-yl)-1,2,6,7,8,11,12,13,14,15,16,17-dodecahydro-3*H*-cyclopenta[a]phenanthren-3-one (3v)

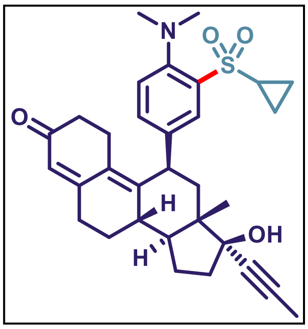

Synthesized from aniline derivative **1l** (173 mg, 0.4 mmol) and sulfinate **2j** (154 mg, 1.2 mmol) following the GP-1. Isolated after flash column chromatography (hexane:EtOAc, 100:0 to 40:60) as yellow oil; 91% yield (193 mg, green light, 2 h) and 93% (197 mg, red light, 12 h).

Melting range: 164-167 °C

^{1}H NMR (300 MHz, $CDCl_3$): δ 7.76 (s, 1H), 7.42 – 7.29 (m, 2H), 5.77 (s, 1H), 4.42 (d, *J* = 6.9 Hz, 1H), 3.40 – 3.24 (m, 1H), 2.82 – 2.69 (m, 1H), 2.76 (s, 6H), 2.63 – 2.51 (m, 2H), 2.49 – 2.34 (m, 4H), 2.32 – 2.16 (m, 3H), 2.08 – 1.91 (m, 3H), 1.88 (s, 3H), 1.80 – 1.63 (m, 3H), 1.53 – 1.29 (m, 2H), 1.26 – 1.08 (s, 2H), 0.98 – 0.84 (m, 2H), 0.52 – 0.41 (s, 2H).

^{13}C NMR (75.5 MHz, $CDCl_3$): δ 199.4, 156.6, 151.9, 144.7, 142.0, 137.1, 132.7, 130.1, 127.9, 123.9, 123.4, 82.9, 82.3, 80.1, 77.4, 49.8, 47.0, 46.4, 40.1, 39.2, 39.2, 38.9, 36.9, 31.5, 31.2, 27.5, 26.1, 23.4, 14.1, 5.6, 5.5, 3.9.

HRMS (APCI, m/z): calculated for $C_{32}H_{40}NO_4S$ [M^+ + H]: 534.2673; found: 534.2661.

Specific rotation: $[\alpha]_D^{20}$ + 53.6 (*c*=1.0, $CHCl_3$).

(*S*)-*N*-{[3-(3 and 2-(cyclopropylsulfonyl)-5-fluoro-4-morpholinophenyl)-2-oxooxazolidin-5-yl]methyl}acetamide (3w) and (3w′)

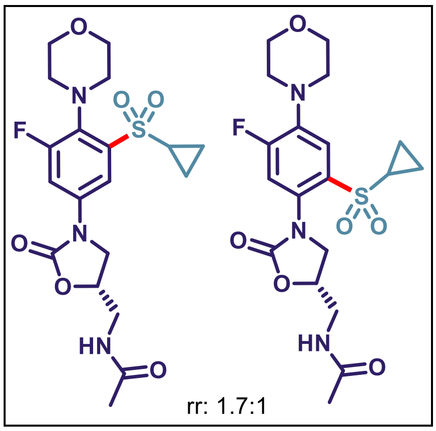

Synthesized from aniline derivative **1m** (135 mg, 0.4 mmol) and sulfinate **2j** (154 mg, 1.2 mmol) following the GP-1. Isolated after flash column chromatography (EtOAc:MeOH, 100:0 to 97:3) as a yellow oil; 90% yield (159 mg, green light, 2 h) and 84% (148 mg, red light, 12 h).

^{1}H NMR (300 MHz, $CDCl_3$):*Signals of the major isomer*: δ 7.88 (dd, *J* = 14.0, 2.5 Hz, 1H), 7.56 – 7.48 (m, 1H), 6.62 (t, *J* = 5.8 Hz, 1H) 4.85 – 4.73 (m, 1H), 4.13 – 4.04 (m, 1H), 3.94 – 3.85 (m, 2H), 3.80 – 3.74 (m, 1H), 3.80 – 3.67 (m, 2H), 3.67 – 3.54 (m, 2H), 3.50 – 3.39 (m, 2H), 3.39 – 3.28 (m, 1H), 2.98 – 2.81 (m, 2H), 1.99 (s, 3H), 1.24 – 1.17 (m, 2H), 1.04 – 0.95 (m, 2H). *Signals of the minor isomer*: δ 7.37 (d, *J* = 8.9 Hz, 1H), 7.10 (d, *J* = 12.4 Hz, 1H), 6.53 (t, *J* = 5.7 Hz, 1H), 4.95 – 4.85 (m, 1H), 4.04 – 3.96 (m, 1H), 3.87 – 3.81 (m, 4H), 3.78 – 3.72 (m, 1H), 3.74 – 3.62 (m, 2H), 3.19 – 3.09 (m, 4H), 2.98 – 2.81 (m, 1H), 2.03 (s, 3H), 1.27 – 1.24 (m, 2H), 1.10 – 1.04 (m, 2H).

^{13}C NMR (126 MHz, $CDCl_3$): *Signals of the major isomer*: δ 171.3, 162.9 (d, *J* = 256.6 Hz), 154.2, 141.7 (d, *J* = 6.4 Hz), 137.2 (d, *J* = 11.4 Hz), 133.0 (d, *J* = 13.6 Hz), 113.1 (d, *J* = 2.5 Hz), 112.1 (d, *J* = 26.8 Hz), 72.2, 67.4, 51.4 (d, *J* = 4.0 Hz), 47.5, 42.0, 31.8, 23.1, 6.0. *Signals of the minor isomer*: δ 171.2, 157.6 (d, *J* = 258.4 Hz), 157.2, 141.1 (d, *J* = 8.2 Hz), 135.5, 129.7 (d, *J* = 10.4 Hz), 120.6 (d, *J* = 5.4 Hz), 119.1 (d, *J* = 22.7 Hz), 73.4, 66.7, 52.2, 50.3 (d, *J* = 3.7 Hz), 41.9, 32.6, 23.2, 6.0.

^{19}F NMR (282 MHz, $CDCl_3$) *Signals of the major isomer*: δ –113.07 (d, *J* = 13.7 Hz, 1F). *Signals of the minor isomer*: δ –111.21 (dd, *J* = 11.0, 0.5 Hz, 1F)

HRMS (APCI, m/z): calculated for $C_{19}H_{25}FN_3O_6S$ [M^+ + H]: 442,1443; found: 442,1443.

Specific rotation: $[\alpha]_D^{20}$ – 7.6 (*c*=1.0, $CHCl_3$).

10. Apparent quantum yield (A.Q.Y.) calculations

Apparent quantum yield (A.Q.Y.) was calculated according to the previous literature.[16]

Apparent quantum yield (A.Q.Y.) = (mmol of product/mmol incident photons) x 100

$$E_{photon\ inc} = \frac{hc}{\lambda} = \frac{(6.63 \text{ x } 10^{-34}\ Js) \text{ x } (3 \text{ x } 10^{8}\ ms^{-1})}{525 \text{ x } 10^{-9}\ m} = 3.79 \text{ x } 10^{-19}\ J$$

Where h (J·s) is Planck's constant, c (ms^{-1}) is the speed of light and λ (m) is the wavelength of the incident light.

$E_{total} = PSt$ = (0.004 W/cm^{-2}) x (1 cm^2) x (2 x 3600 s) = 28.8 J

Where P (Wcm^{-2}) is the power density of the incident light,[xx] S (cm^2) is the irradiation area and t (s) is the photoreaction time.

Number of incident photons = $E_{total}/E_{photon\ inc} = \frac{28.8\ J}{3.79 \text{ x } 10^{-19}\ J} = 7.6 \text{ x } 10^{19}$

mmol of incident photons = $\frac{7.6 \text{ x } 10^{19}}{6.022 \text{ x } 10^{23}}$ = 1.26 x 10^{-4} mol = 0.126 mmol

A.Q.Y.(%) = (mmol of product/mmol incident photons) = $\frac{0.95 \text{ mmol}}{0.126 \text{ mmol}}$ x 100 = 75.4%.

11. Comparison of this work with the homogeneous Ir(III) photocatalyzed protocol.[9]

Examples	Chem. Sci., **2018**, 9, 629<br>*[Ir(dF(CF$_3$)ppy)$_2$(dtbpy)]PF$_6$ (1 mol%)*<br>*Sulfinate (5 equiv)*<br>*Blue LEDs, 72 h*<br>**Yield (%):**	This work<br>*Tp-BT-COF (0.035 mol%)*<br>*Sulfinate (3 equiv)*<br>*Red LEDs, 12 h*<br>**Yield (%):**
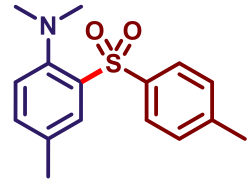	**54**	**85**
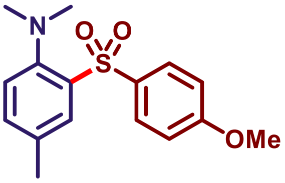	**73**	**78**
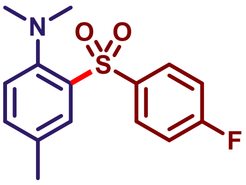	**45**	**86**
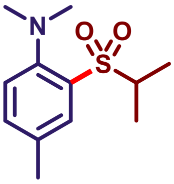	**27**	**62**
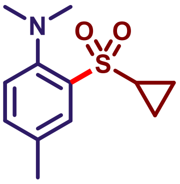	**60**	**86**

Figure S17. Comparison with the Ir(III) photocatalyzed protocol

12. References

(1) Osterrieth, J. W. M.; Rampersad, J.; Madden, D.; Rampal, N.; Skoric, L.; Connolly, B.; Allendorf, M. D.; Stavila, V.; Snider, J. L.; Ameloot, R.; Marreiros, J.; Ania, C.; Azevedo, D.; Vilarrasa-Garcia, E.; Santos, B. F.; Bu, X.; Chang, Z.; Bunzen, H.; Champness, N. R.; Griffin, S. L.; Chen, B.; Lin, R. B.; Coasne, B.; Cohen, S.; Moreton, J. C.; Colón, Y. J.; Chen, L.; Clowes, R.; Coudert, F. X.; Cui, Y.; Hou, B.; D'Alessandro, D. M.; Doheny, P. W.; Dincă, M.; Sun, C.; Doonan, C.; Huxley, M. T.; Evans, J. D.; Falcaro, P.; Ricco, R.; Farha, O.; Idrees, K. B.; Islamoglu, T.; Feng, P.; Yang, H.; Forgan, R. S.; Bara, D.; Furukawa, S.; Sanchez, E.; Gascon, J.; Telalović, S.; Ghosh, S. K.; Mukherjee, S.; Hill, M. R.; Sadiq, M. M.; Horcajada, P.; Salcedo-Abraira, P.; Kaneko, K.; Kukobat, R.; Kenvin, J.; Keskin, S.; Kitagawa, S.; Otake, K.; Lively, R. P.; DeWitt, S. J. A.; Llewellyn, P.; Lotsch, B. V.; Emmerling, S. T.; Pütz, A. M.; Martí-Gastaldo, C.; Padial, N. M.; García-Martínez, J.; Linares, N.; Maspoch, D.; Suárez del Pino, J. A.; Moghadam, P.; Oktavian, R.; Morris, R. E.; Wheatley, P. S.; Navarro, J.; Petit, C.; Danaci, D.; Rosseinsky, M. J.; Katsoulidis, A. P.; Schröder, M.; Han, X.; Yang, S.; Serre, C.; Mouchaham, G.; Sholl, D. S.; Thyagarajan, R.; Siderius, D. S.; Snurr, R. Q.; Goncalves, R. B.; Telfer, S.; Lee, S. J.; Ting, V. P.; Rowlandson, J. L.; Uemura, T.; Iiyuka, T.; van der Veen, M. A.; Rega, D.; Van Speybroeck, V.; Rogge, S. M. J.; Lamaire, A.; Walton, K. S.; Bingel, L. W.; Wuttke, S.; Andreo, J.; Yaghi, O.; Zhang, B.; Yavuz, C. T.; Nguyen, T. S.; Zamora, F.; Montoro, C.; Zhou, H.; Kirchon, A.; Fairen-Jimenez, D. How Reproducible are Surface Areas Calculated from the BET Equation? *Adv. Mater.* **2022**, *34*, 2201502.

(2) Wang, R.; Kong, W.; Zhou, T.; Wang, C.; Guo, J. Organobase modulated synthesis of high-quality β-ketoenamine-linked covalent organic frameworks. *Chem. Commun.*, **2021**, *57*, 331–334.

(3) Yang, S.; Li, X.; Qin, Y.; Cheng, Y.; Fan, W.; Lang, X.; Zheng, L.; Cao, Q. Modulating the Stacking Model of Covalent Organic Framework Isomers with Different Generation Efficiencies of Reactive Oxygen Species. *ACS Appl. Mater. Interfaces* **2021**, *13*, 29471−29481.

(4) Murár, M.; Addová, G.; Boháč, A. Synthesis of 5-(ethylsulfonyl)-2-methoxyaniline: An important pharmacological fragment of VEGFR2 and other inhibitors. *Beilstein J. Org. Chem.* **2013**, *9*, 173–179.

(5) Zhang, L.; Cheng, X.; Zhou, Q.-L. Electrochemical Synthesis of Sulfonyl Fluorides with Triethylamine Hydrofluoride. *Chin. J. Chem.* **2022**, *40*, 1687–1692.

(6) Guzzo, P. R.; Henderson, A. J.; Nacro, K.; Isherwood, M. L.; Ghosh, A.; Xiang, K. Epiminocycloalkyl(b)indole derivatives as serotonin sub-type 6 (5-ht6) modulators and uses thereof. WO2011/044134A1, April 14, **2011**.

(7) Wei, Z.; Zhao, D.; Du, Y.; Li, Z. Synthesis of *N*-Sulfinyl Sulfoximines from 5-(Sulfoximido)dibenzothiophenium Triflates and Sodium Sulfinates. *J. Org. Chem.* **2024***, 89,* 10311−10315.

(8) Urgaonkar, S.; Verkade, J. G. Scope and Limitations of $Pd_2(dba)_3/P(i\text{-}BuNCH_2CH_2)_3N$-Catalyzed Buchwald−Hartwig Amination Reactions of Aryl Chlorides. *J. Org. Chem.* **2004**, 69, 9135−9142.

(9) Johnson, T. C.; Elbert, B. L.; Farley, A. J. M.; Gorman, T. W.; Genicot, C.; Lallemand, B.; Pasau, P.; Flasz, J.; Castro, J. L.; MacCoss, M.; Dixon, D. J.; Paton, R. S.; Schofield, C. J.; Smith, M. D.; Willis, M. C. Direct sulfonylation of anilines mediated by visible light. *Chem. Sci.* **2018**, *9*, 629–633

(10) Zhou, K.; Zhang, J.; Lai, L.; Cheng, J.; Sun, J.; Wu, J. C–H bond sulfonylation of anilines with the insertion of sulfur dioxide under metal-free conditions. *Chem. Commun.* **2018**, *54*, 7459–7462.

(11) Lu, F.; Li, J.; Wang, T.; Li, Z.; Jiang, M.; Hu, X.; Pei, H.; Yuan, F.; Lu, L.; Lei, A. Electrochemical oxidative C− H sulfonylation of anilines. *Asian J. Org. Chem.* **2019**, *8*, 1838–1841.

(12) Xu, X.-H.; Zhen, J.-S.; Du, X.; Yuan, H.; Li, Y.-H.; Chu, M.-H.; Luo, Y. Visible-light-mediated late-stage sulfonylation of anilines with sulfonamides. *Org. Lett.* **2022**, *24*, 853–858.

(13) Wu, Y.-C.; Jiang, S.-S.; Luo, S.-Z.; Song, R.-J.; Li, J.-H. Transition-metal-and oxidant-free directed anodic C–H sulfonylation of N, N-disubstituted anilines with sulfinates. *Chem. Commun.* **2019**,*55*, 8995–8998.

(14) Yue, H.; Zhu, C.; Rueping, M. Cross-coupling of sodium sulfinates with aryl, heteroaryl, and vinyl halides by nickel/photoredox dual catalysis. *Angew. Chem. Int. Ed.* **2018**, *57*, 1371–1375.

(15) Luo, Y.; Ding, H.; Zhen, J.-S.; Du, X.; Xu, X.-H.; Yuan, H.; Li, Y.-H.; Qi, W.-Y.; Liu, B.-Z.; Lu, S.-M.; Xue, C.; Ding, Q. Catalyst-free arylation of sulfonamides via visible light-mediated deamination. *Chem. Sci.* **2021**, *12*, 9556–9560.

(16) Liu, S.; Pan, W.; Wu, S.; Bu, X.; Xin, S.; Yu, J.; Xu H.; Yang, X. Visible-light-induced tandem radical additioncyclization of 2-aryl phenyl isocyanides catalysed by recyclable covalent organic frameworks, *Green Chem.*, **2019**, *21*, 2905–2910.

13. NMR spectra of compounds

^{1}H NMR (300 MHz, $CDCl_3$) of **1g**

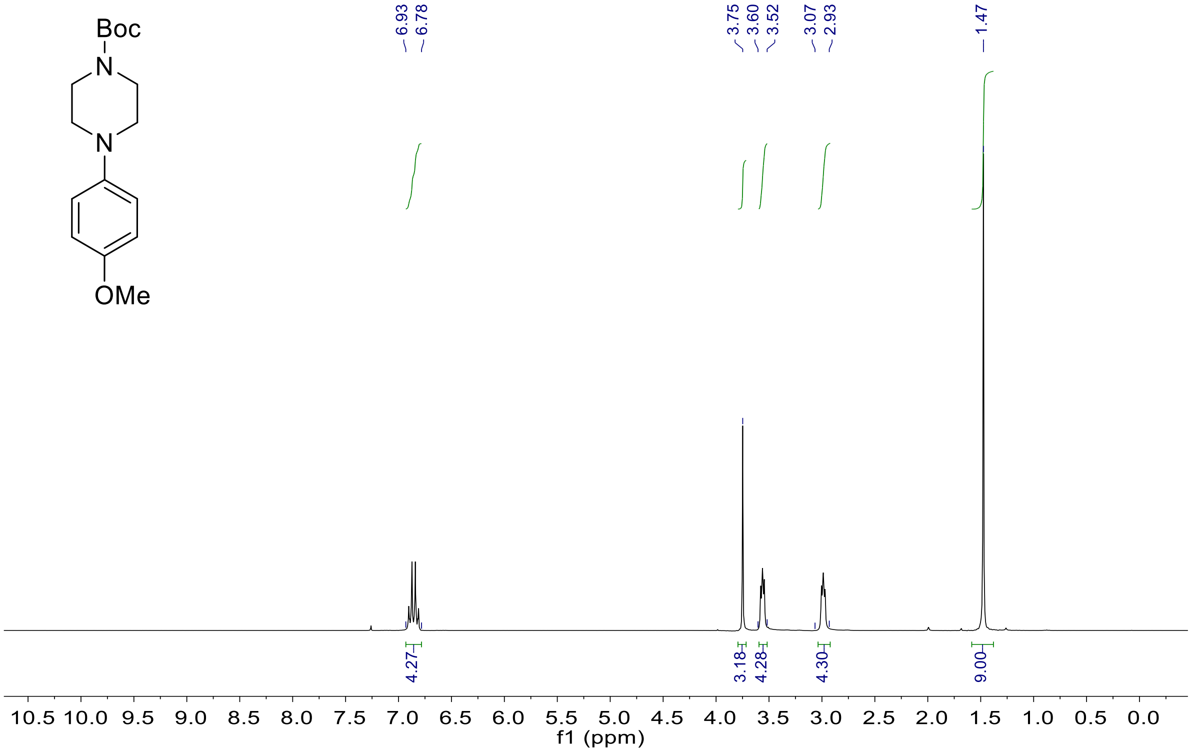

^{13}C NMR (75.5 MHz, $CDCl_3$) of **1g**

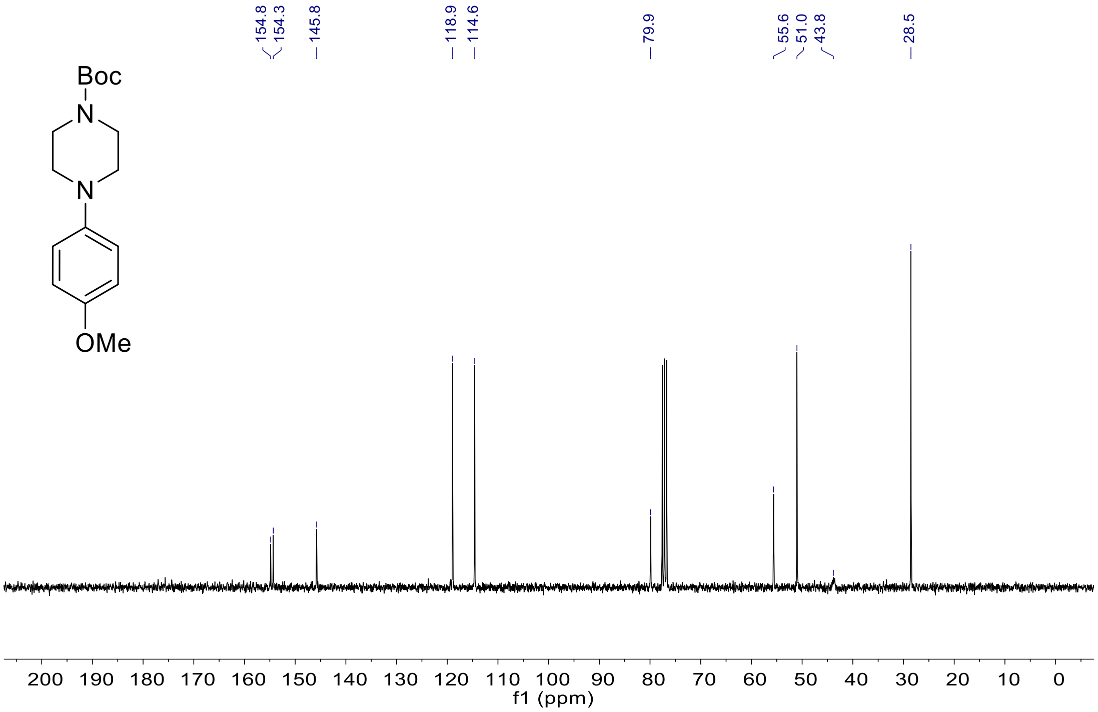

^{1}H NMR (300 MHz, $CDCl_3$) of **2k**

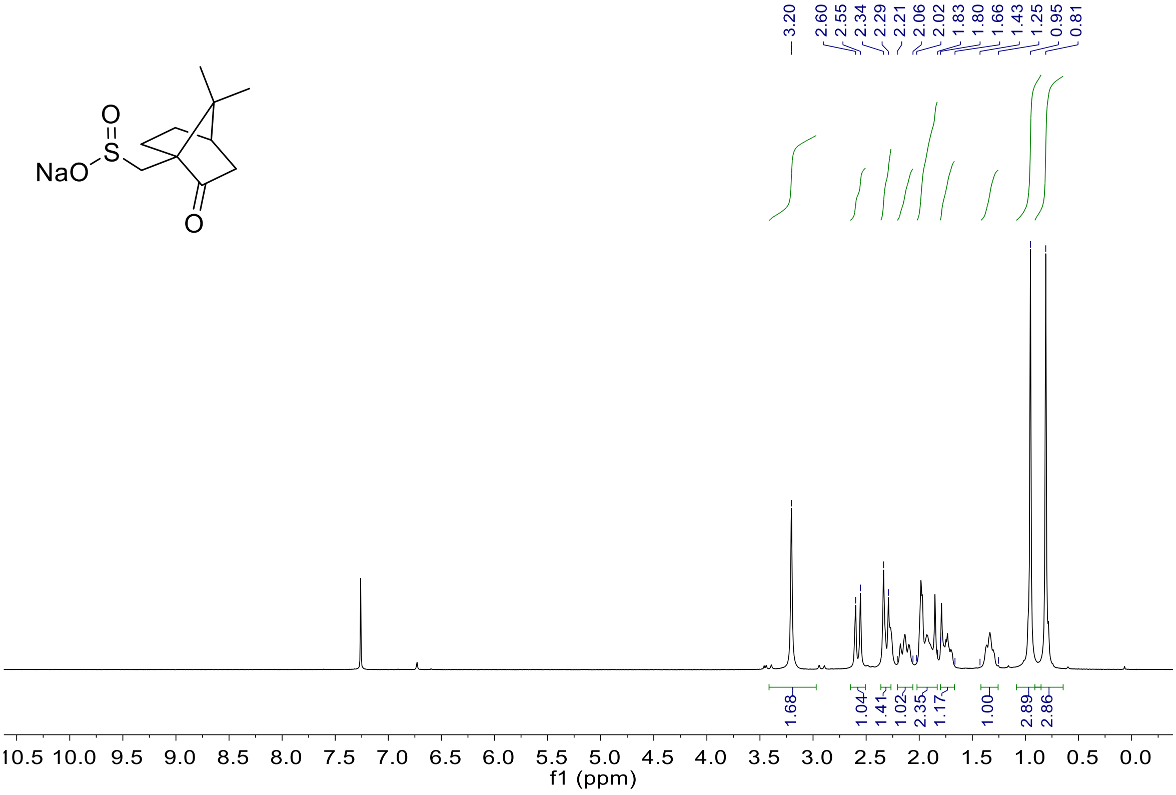

^{13}C NMR (126 MHz, $CDCl_3$) of **2k**

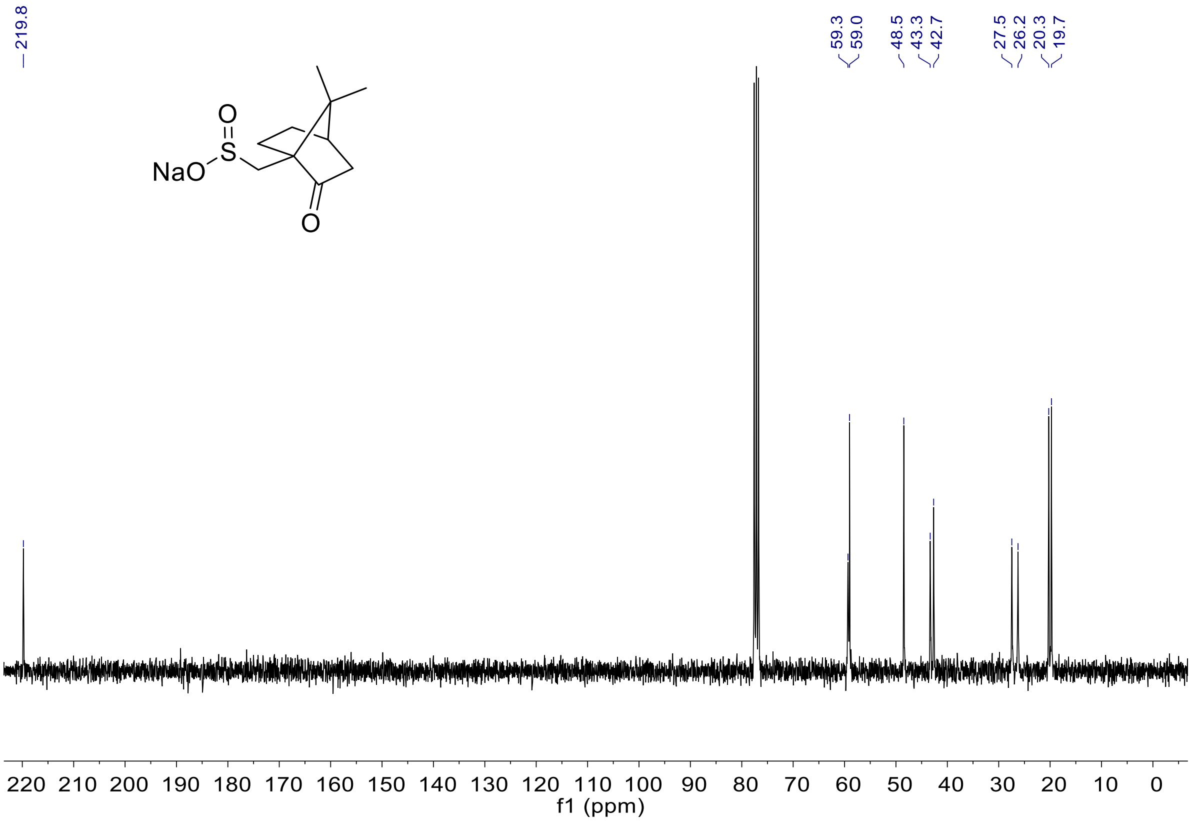

^{1}H NMR (300 MHz, $CDCl_3$) of **3a**

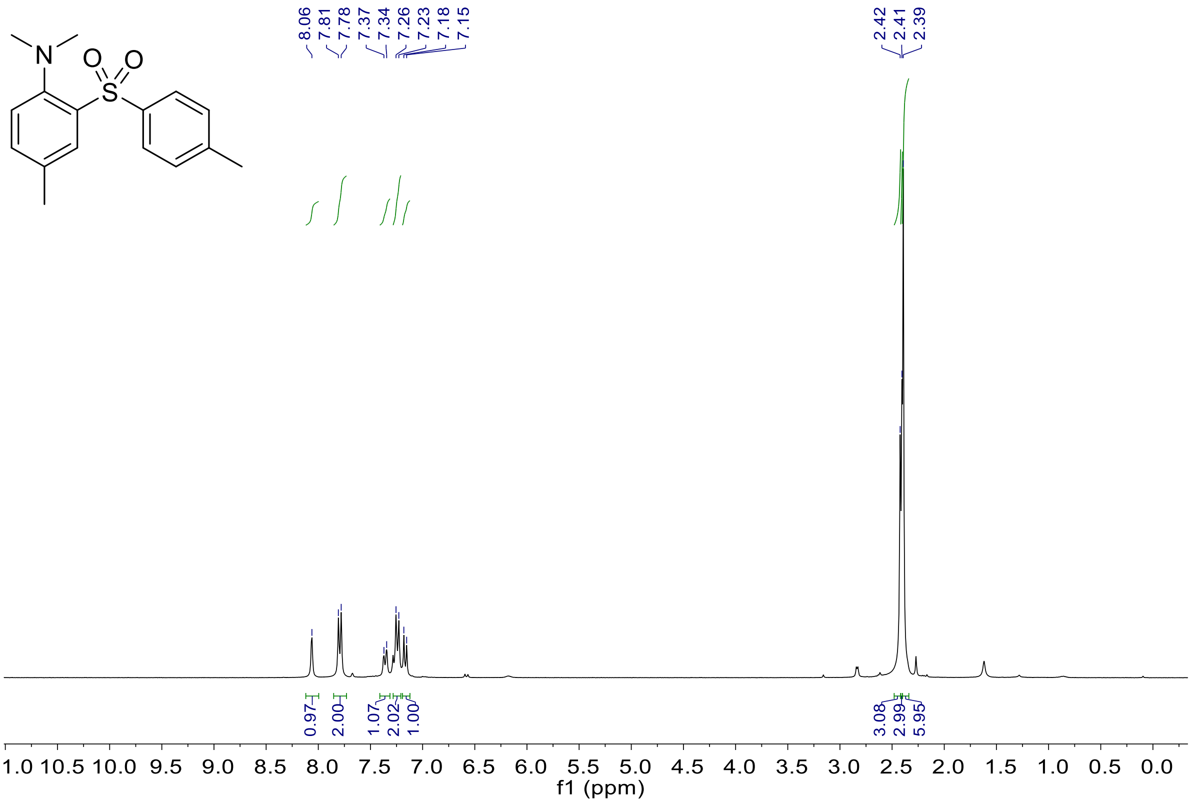

^{13}C NMR (75.5 MHz, $CDCl_3$) of **3a**

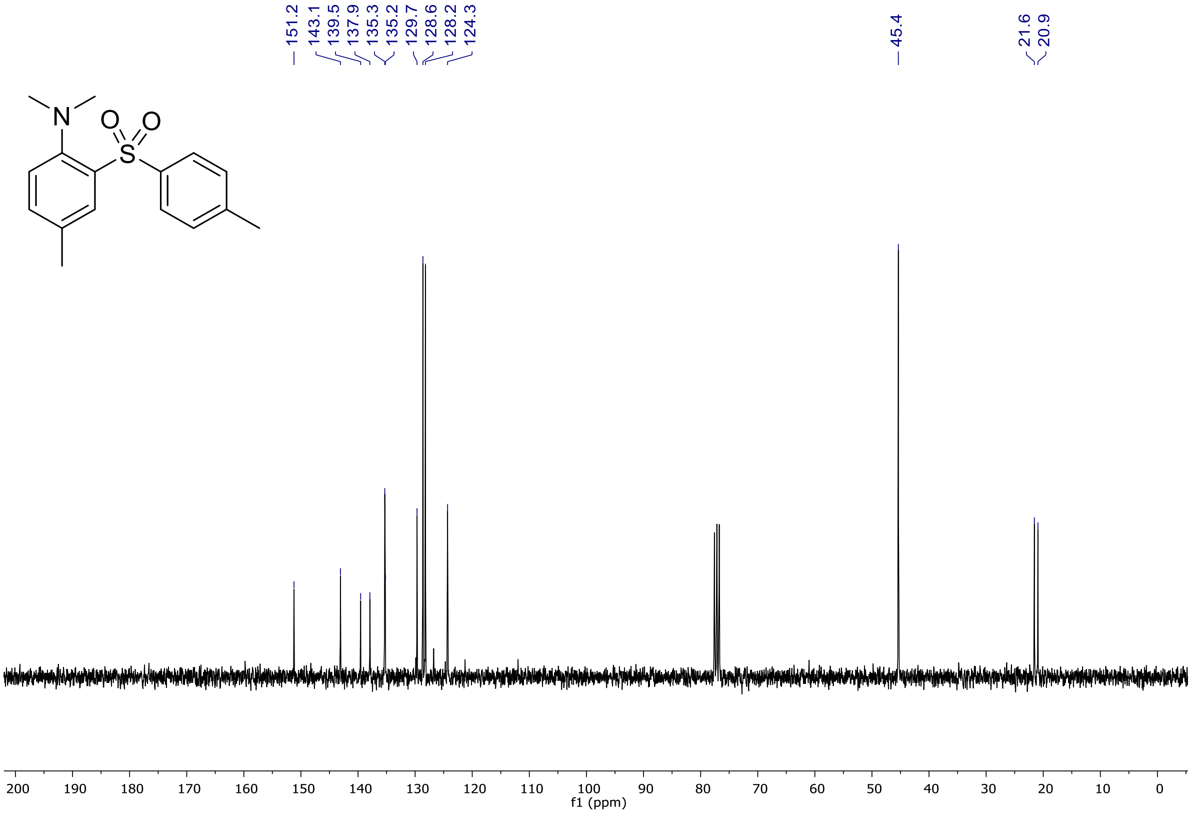

^{1}**H NMR** (300 MHz, $CDCl_3$) of **3b**

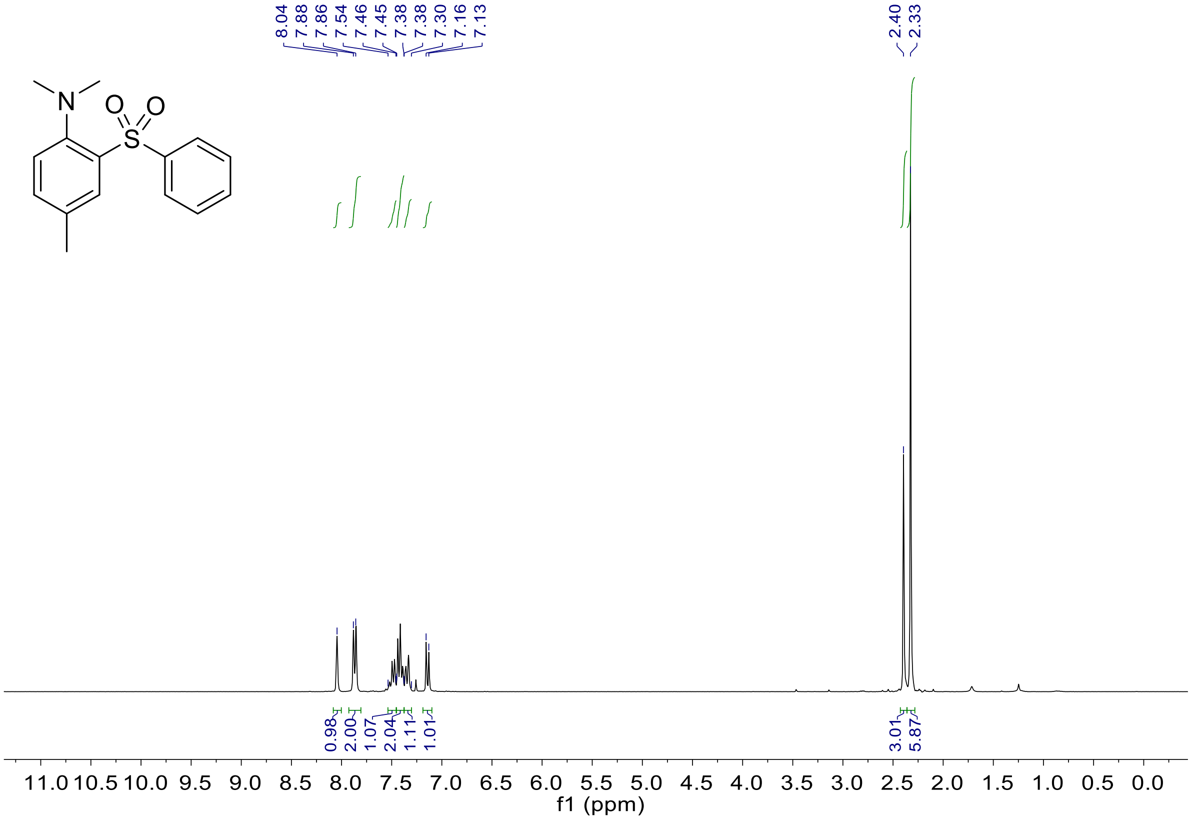

^{13}**C NMR** (75.5 MHz, $CDCl_3$) of **3b**

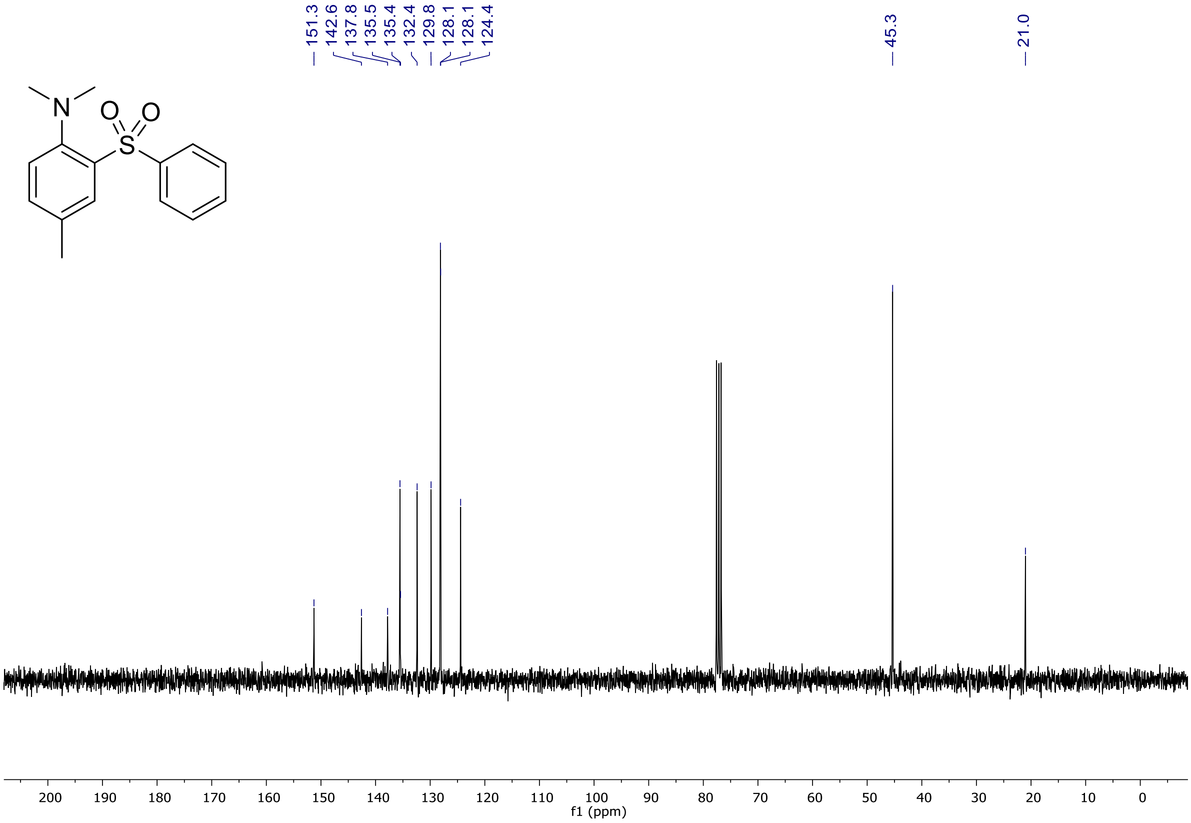

^{1}H NMR (300 MHz, $CDCl_3$) of **3c**

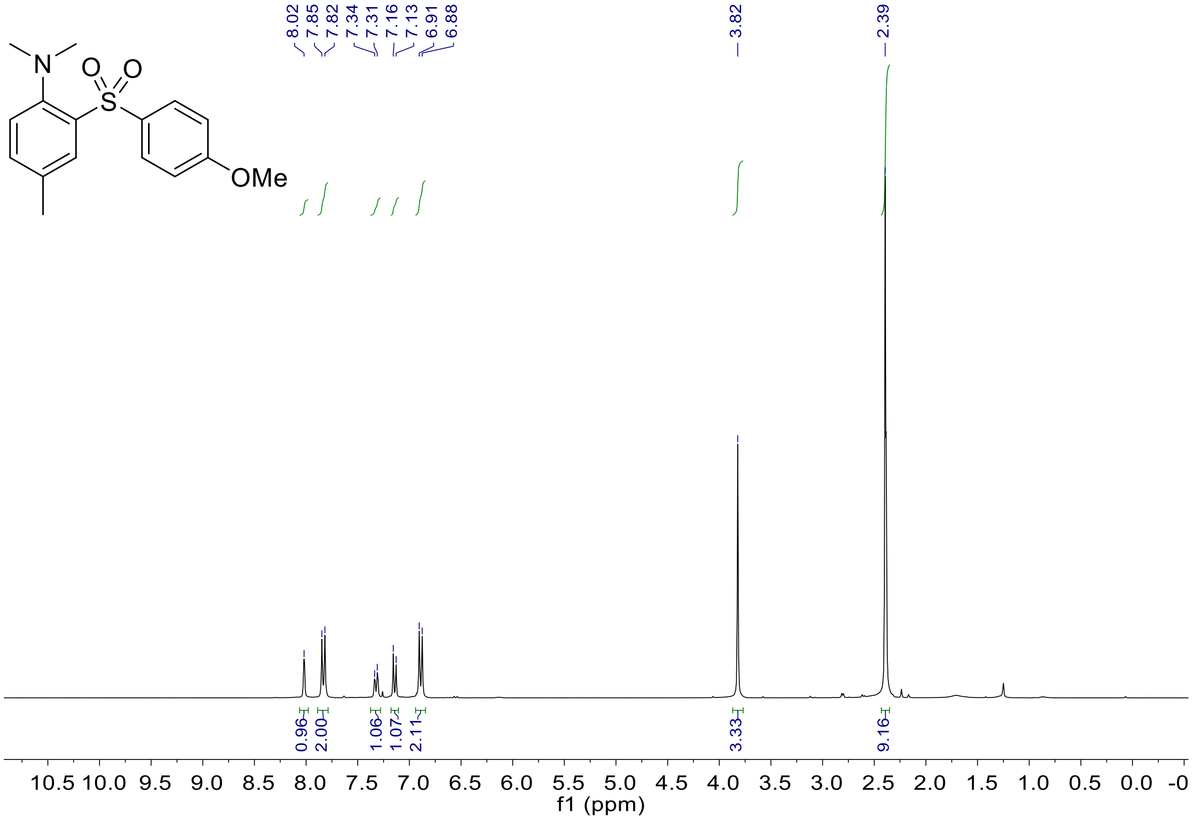

^{13}C NMR (75.5 MHz, $CDCl_3$) of **3c**

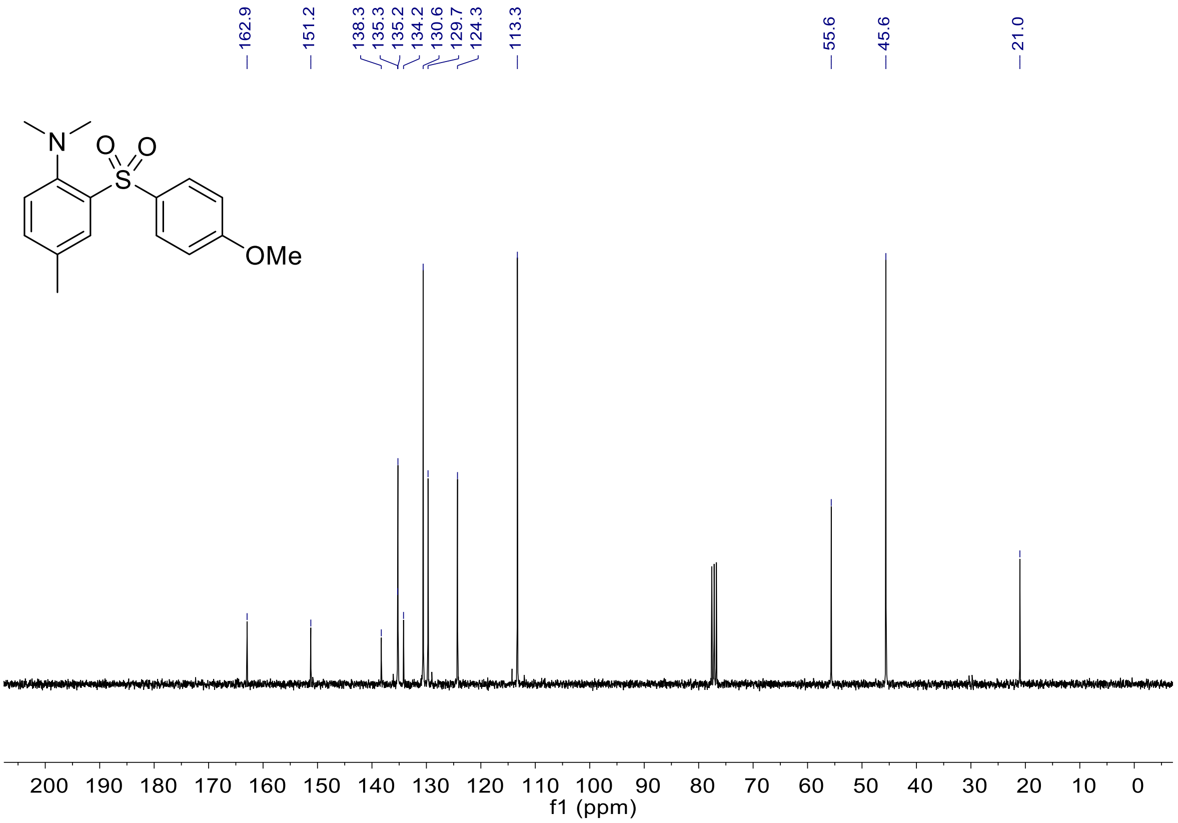

^{1}H NMR (300 MHz, $CDCl_3$) of **3d**

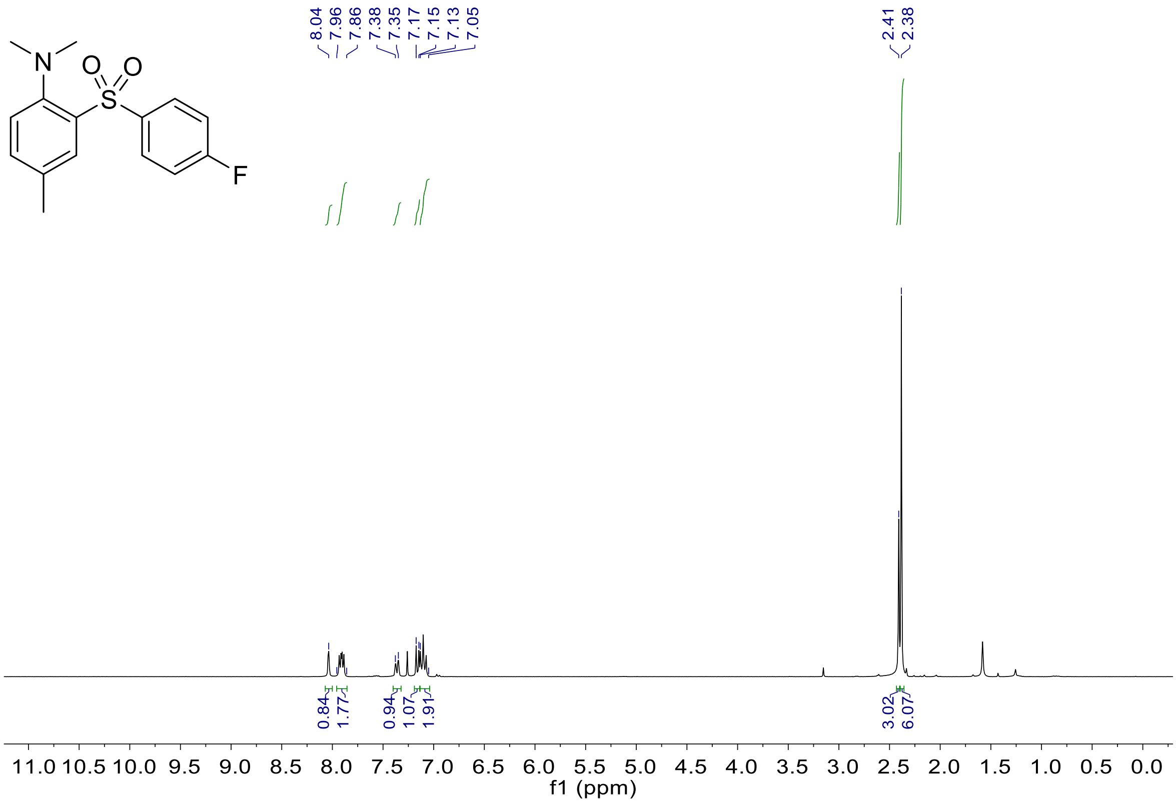

^{13}C NMR (75.5 MHz, $CDCl_3$) of **3d**

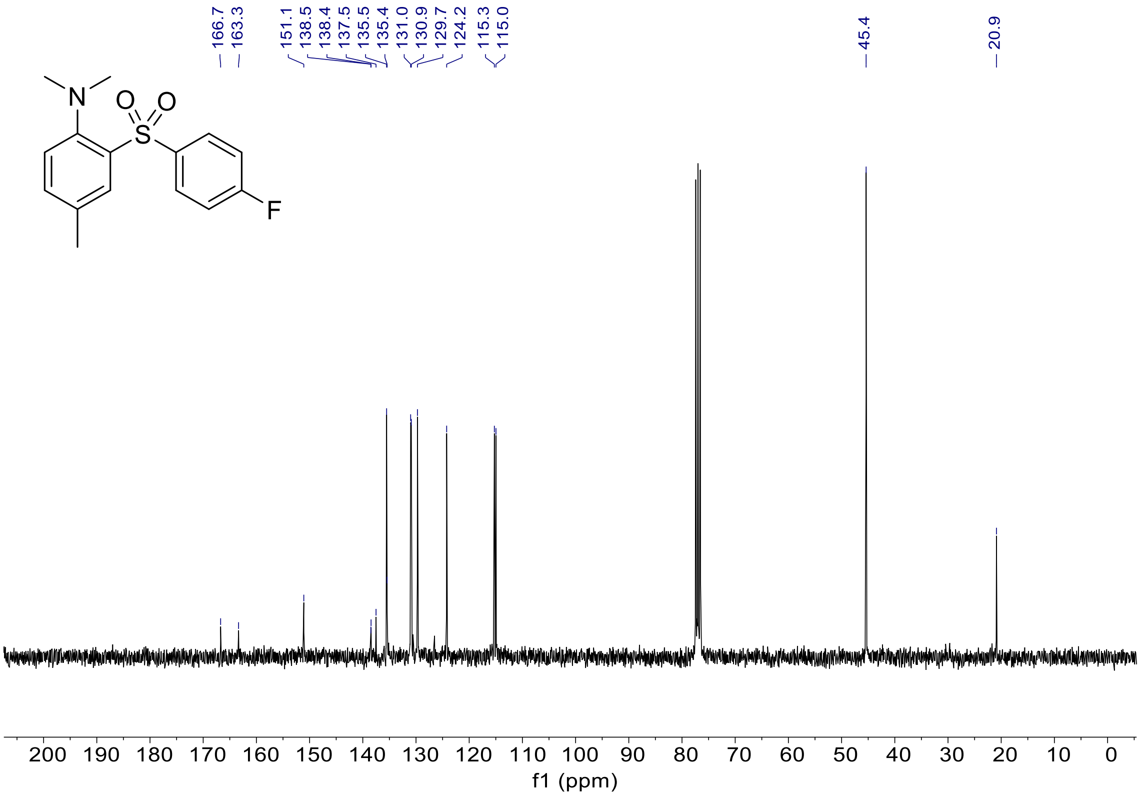

^{19}F NMR (282 MHz, $CDCl_3$) of **3d**

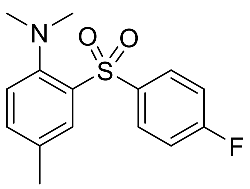

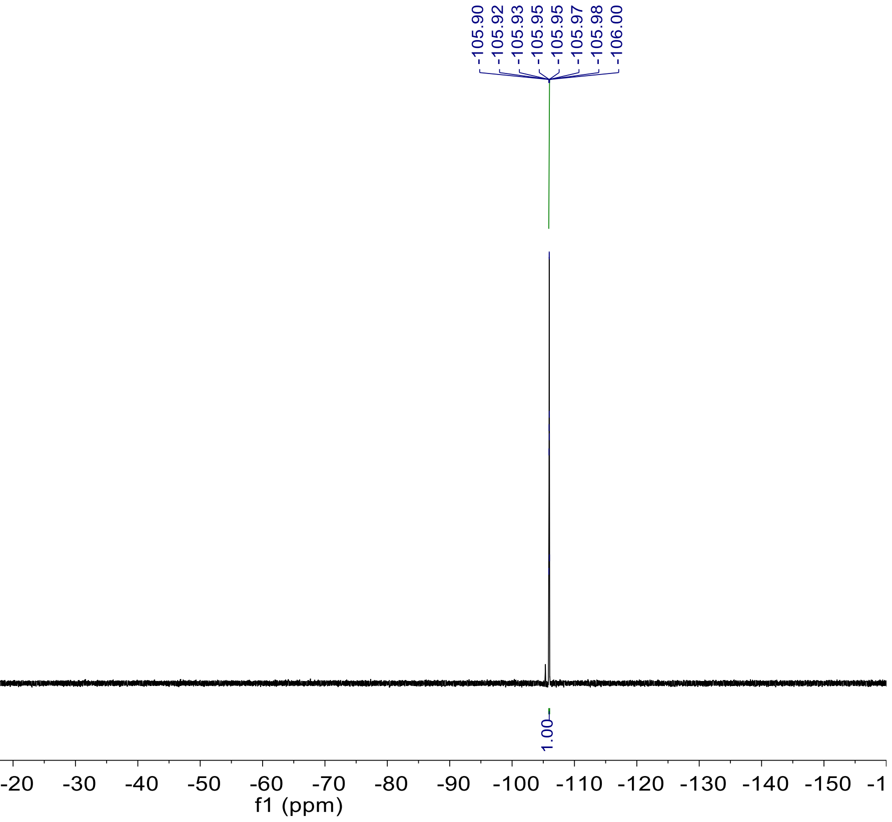

^{1}H NMR (300 MHz, $CDCl_3$) of **3e**

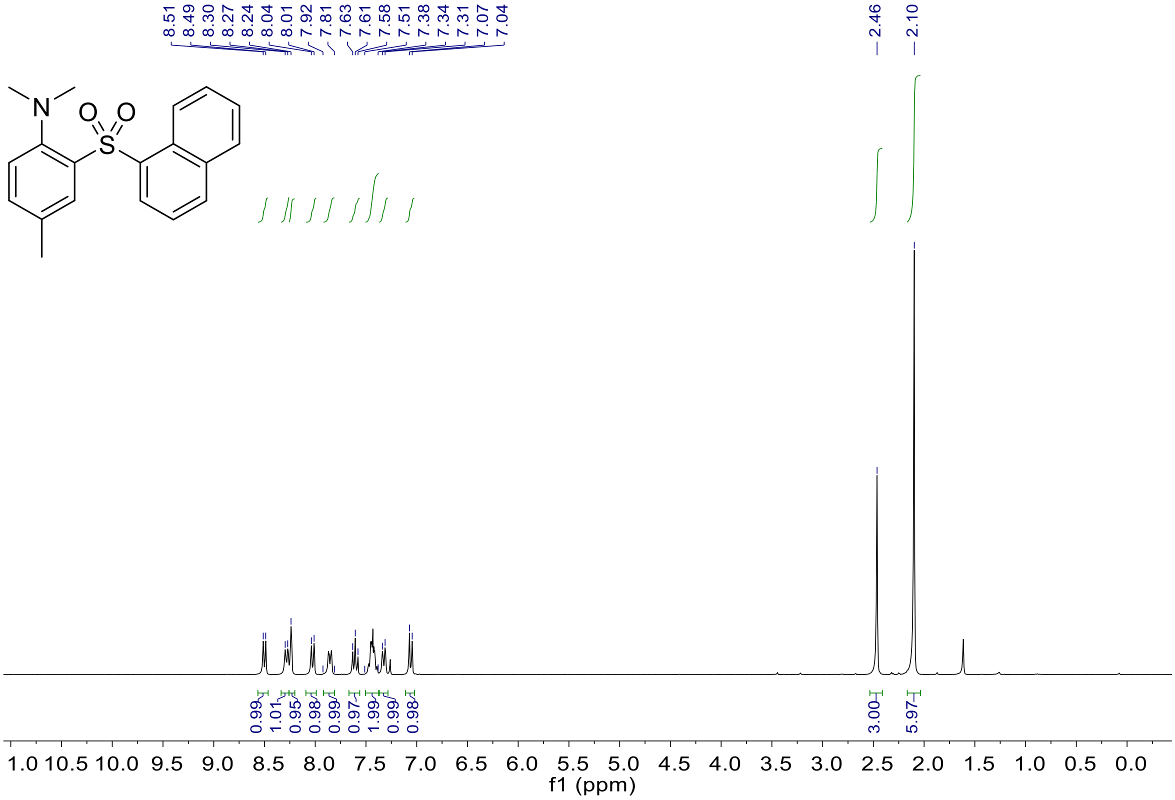

^{13}C NMR (126 MHz, $CDCl_3$) of **3e**

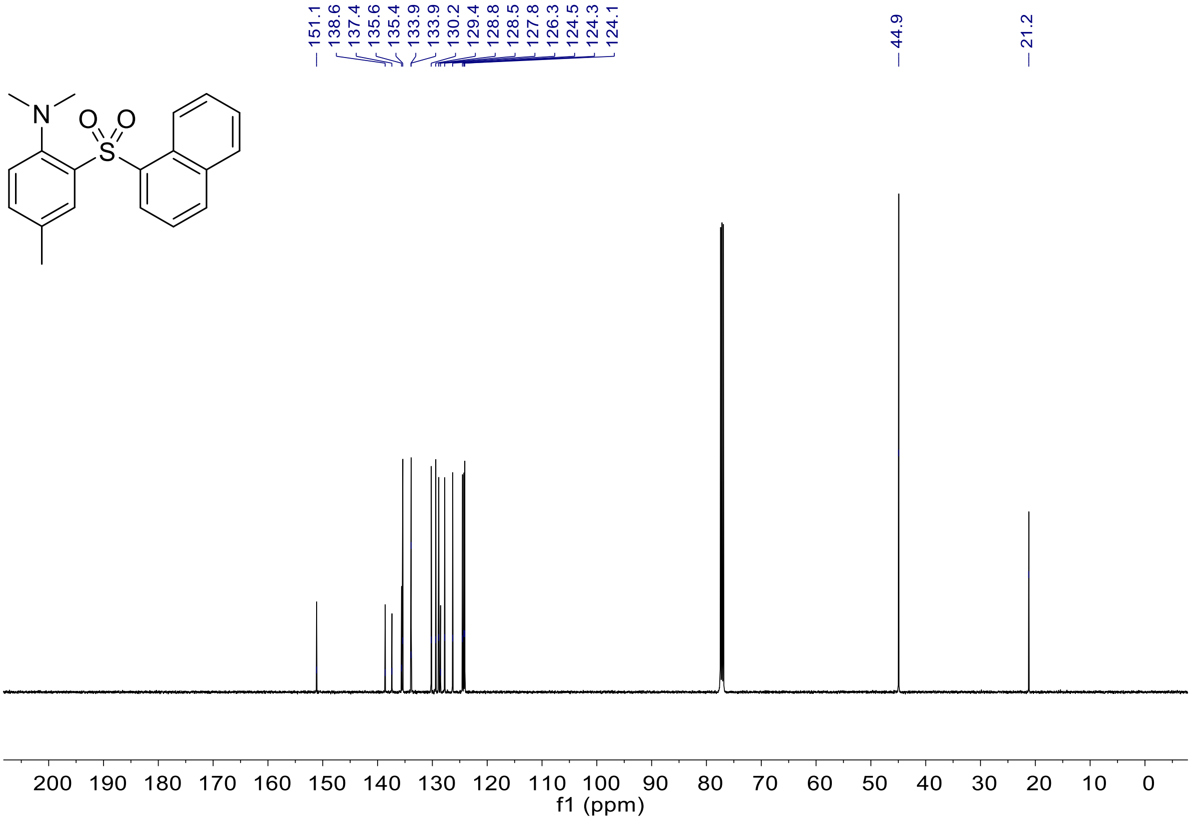

^{1}H NMR (300 MHz, $CDCl_3$) of **3f**

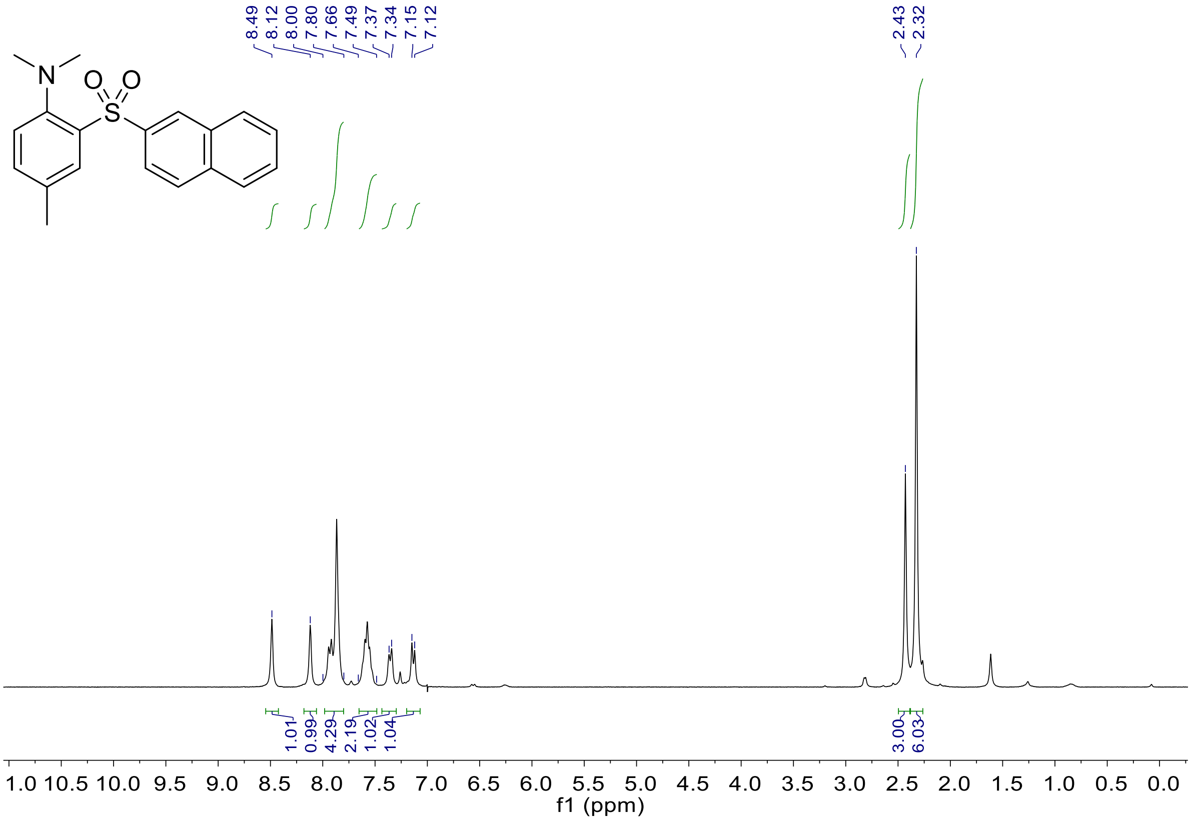

^{13}C NMR (75.5 MHz, $CDCl_3$) of **3f**

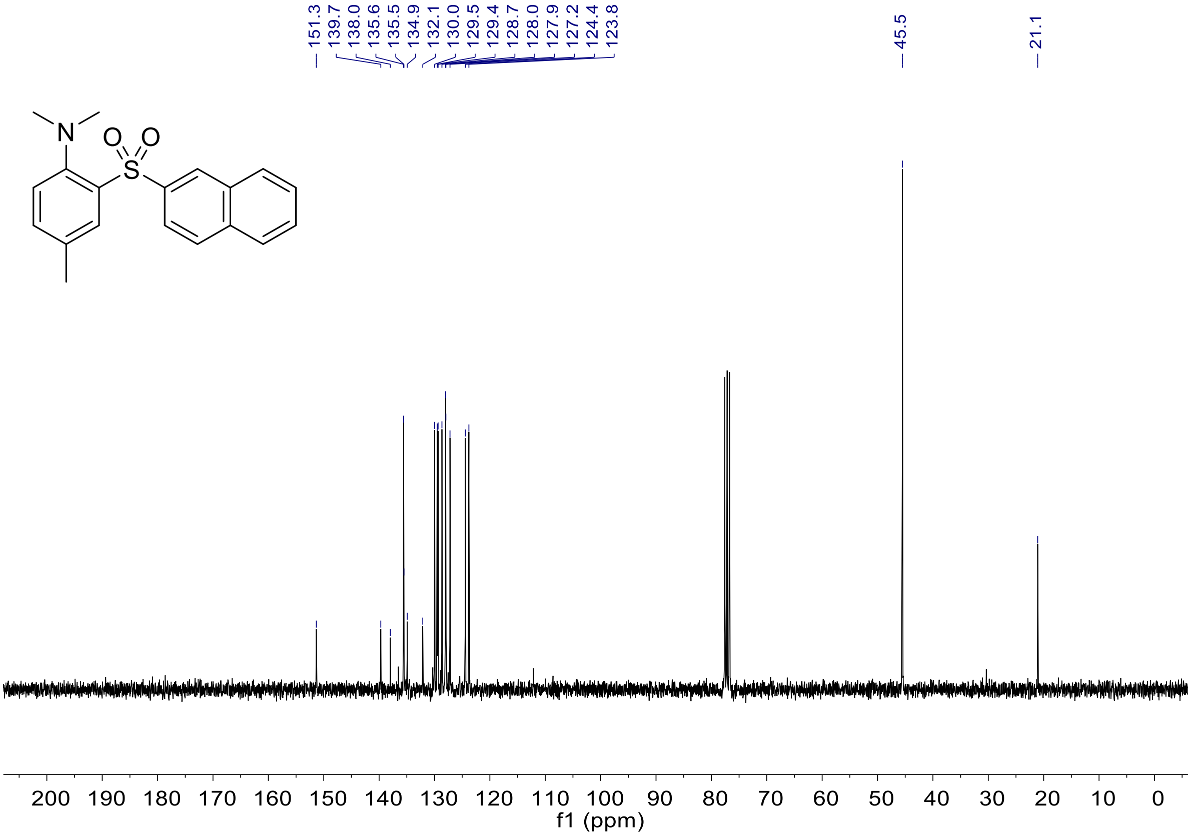

^{1}H NMR (300 MHz, $CDCl_3$) of **3g**

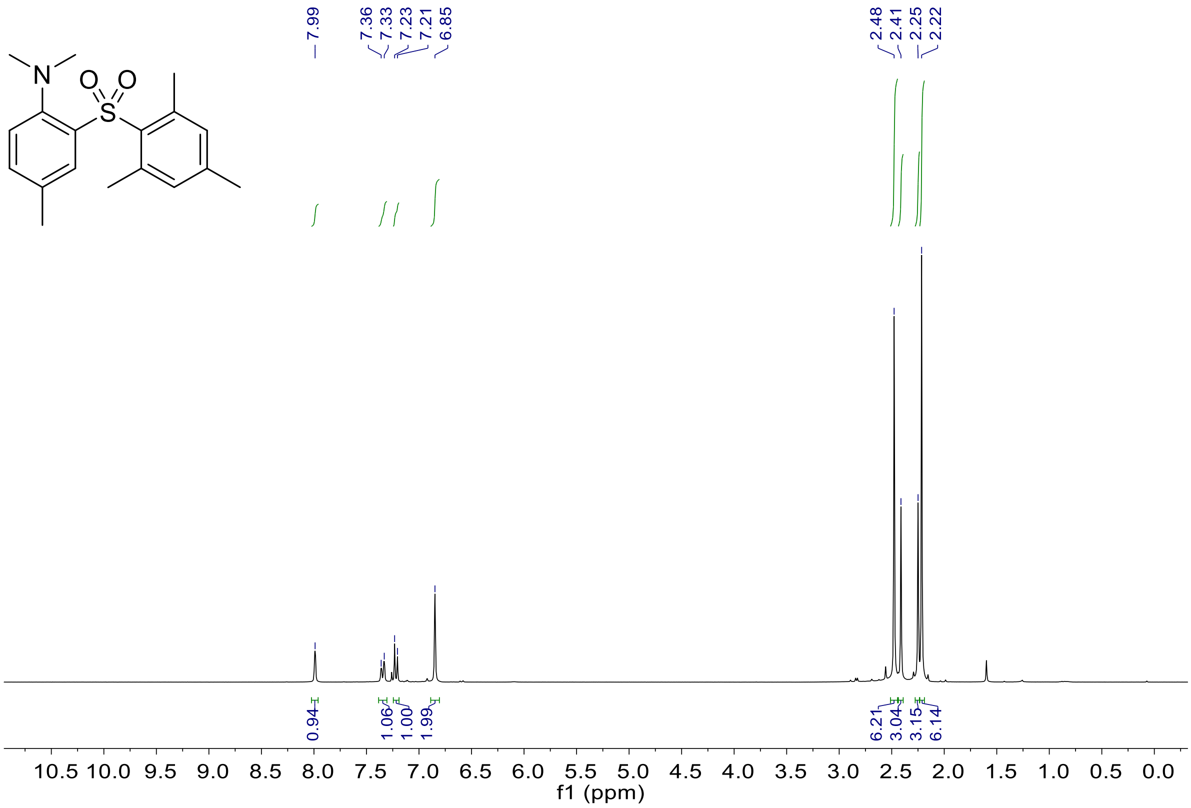

^{13}C NMR (75.5 MHz, $CDCl_3$) of **3g**

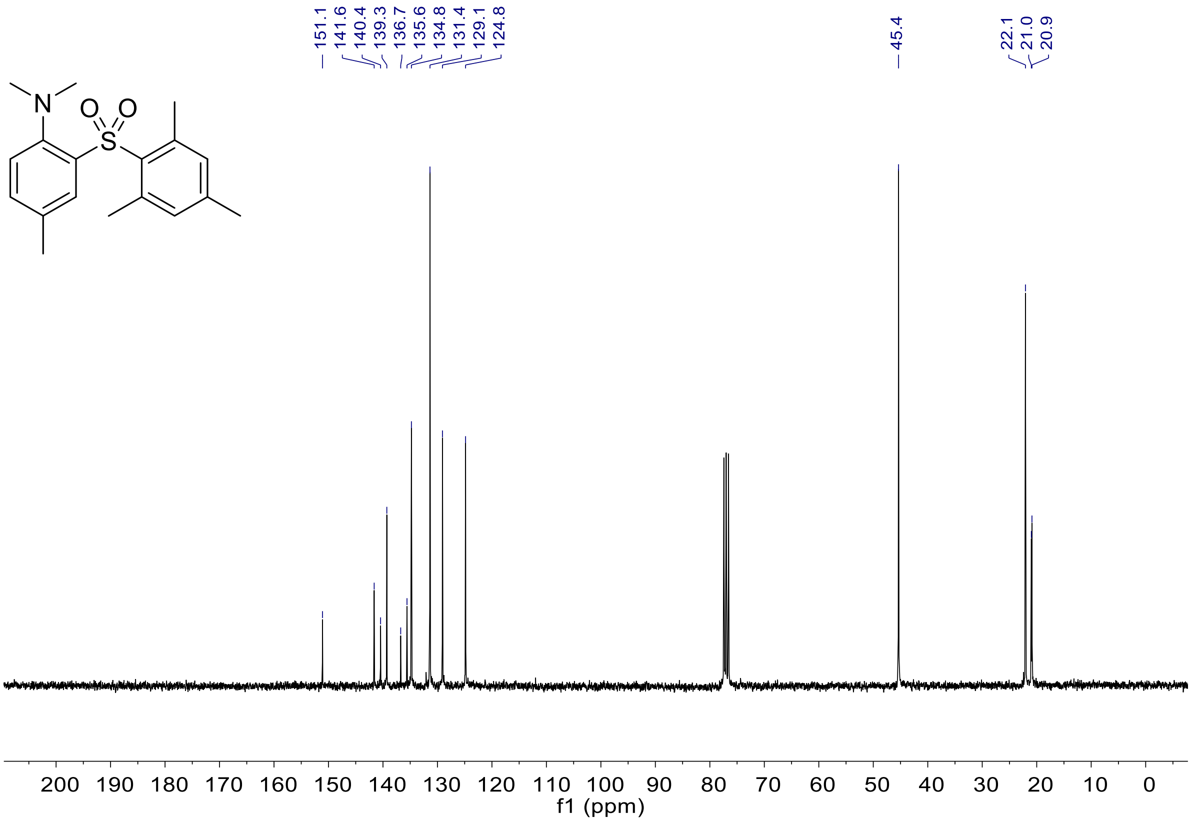

[1]**H NMR** (300 MHz, $CDCl_3$) of **3h**

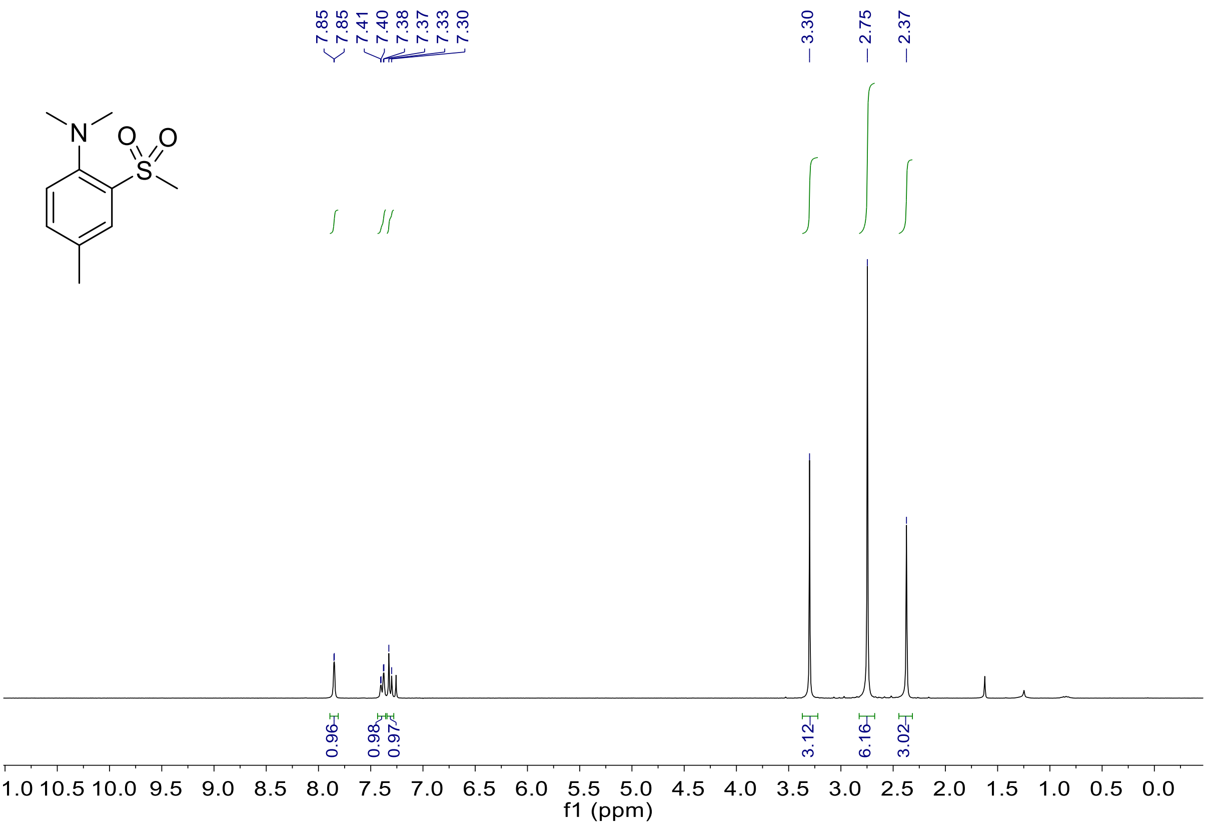

[13]**C NMR** (75.5 MHz, $CDCl_3$) of **3h**

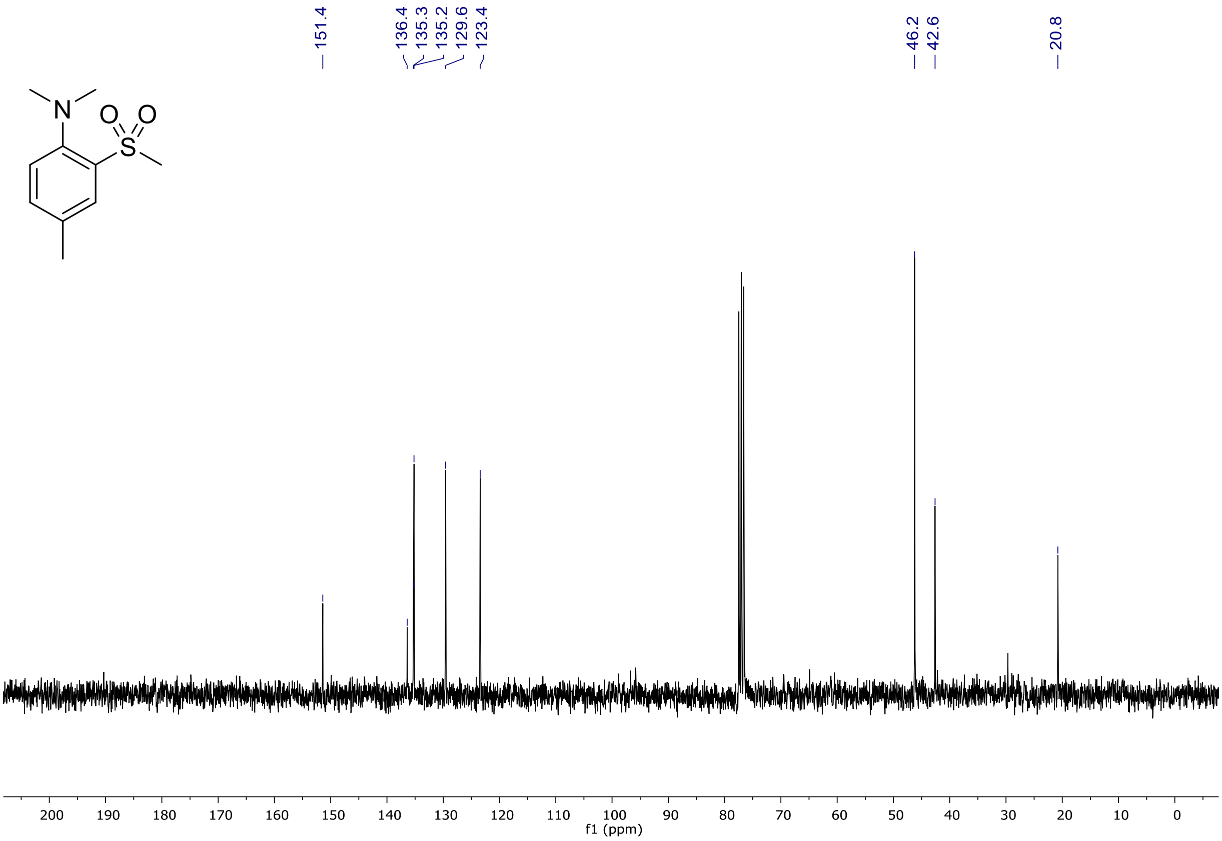

^{1}H NMR (300 MHz, $CDCl_3$) of **3i**

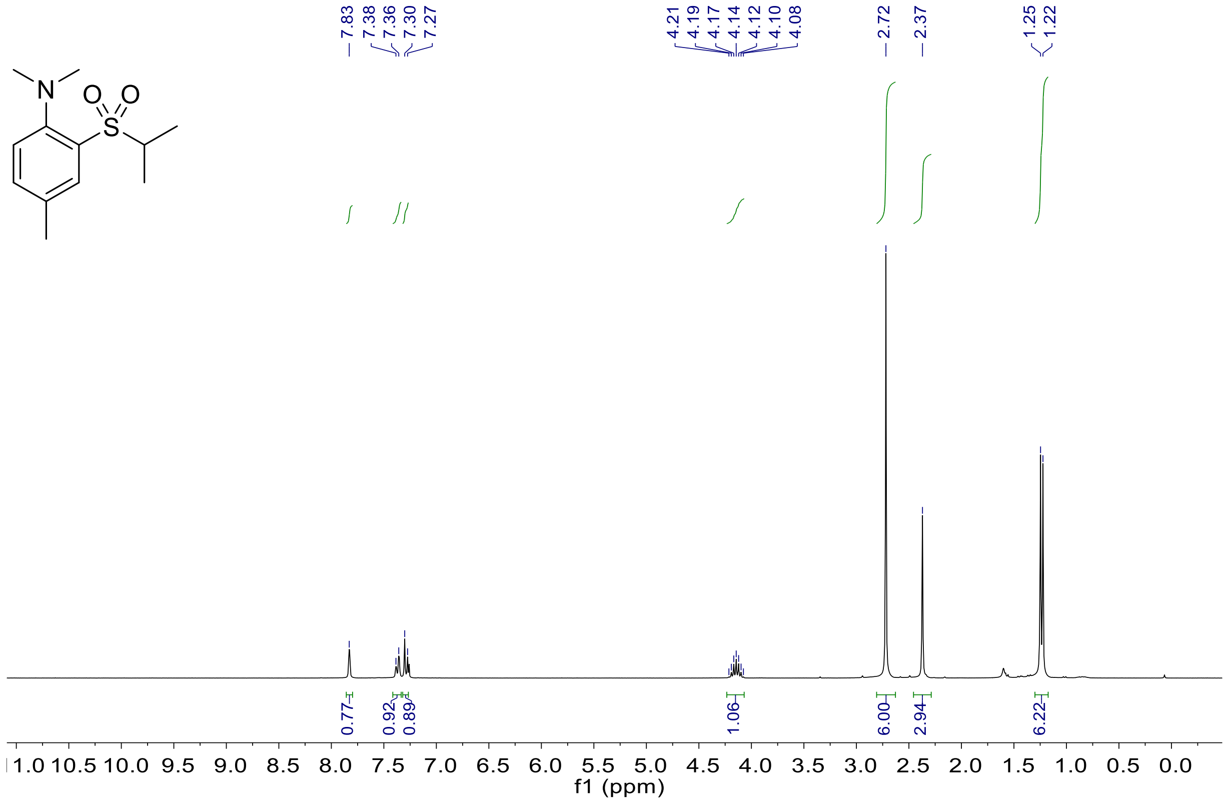

^{13}C NMR (75.5 MHz, $CDCl_3$) of **3i**

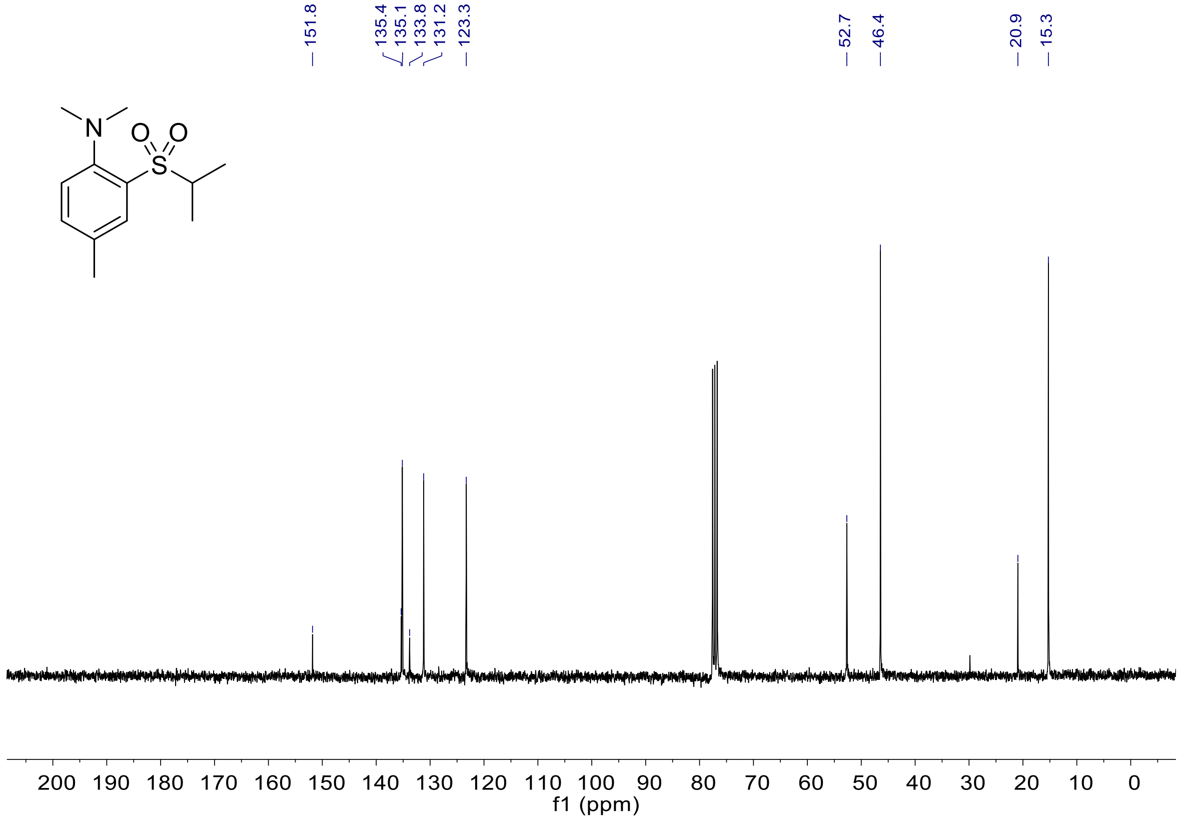

^{1}H NMR (300 MHz, $CDCl_3$) of **3j**

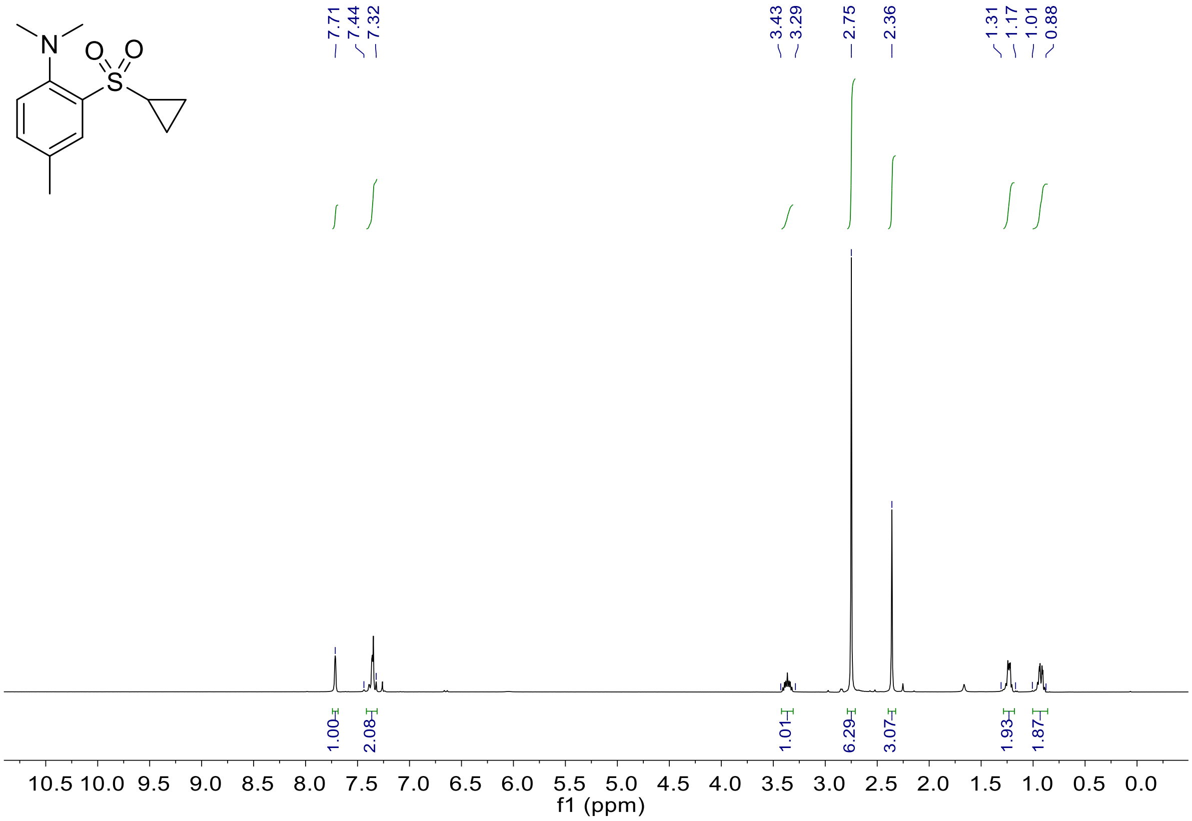

^{13}C NMR (75.5 MHz, $CDCl_3$) of **3j**

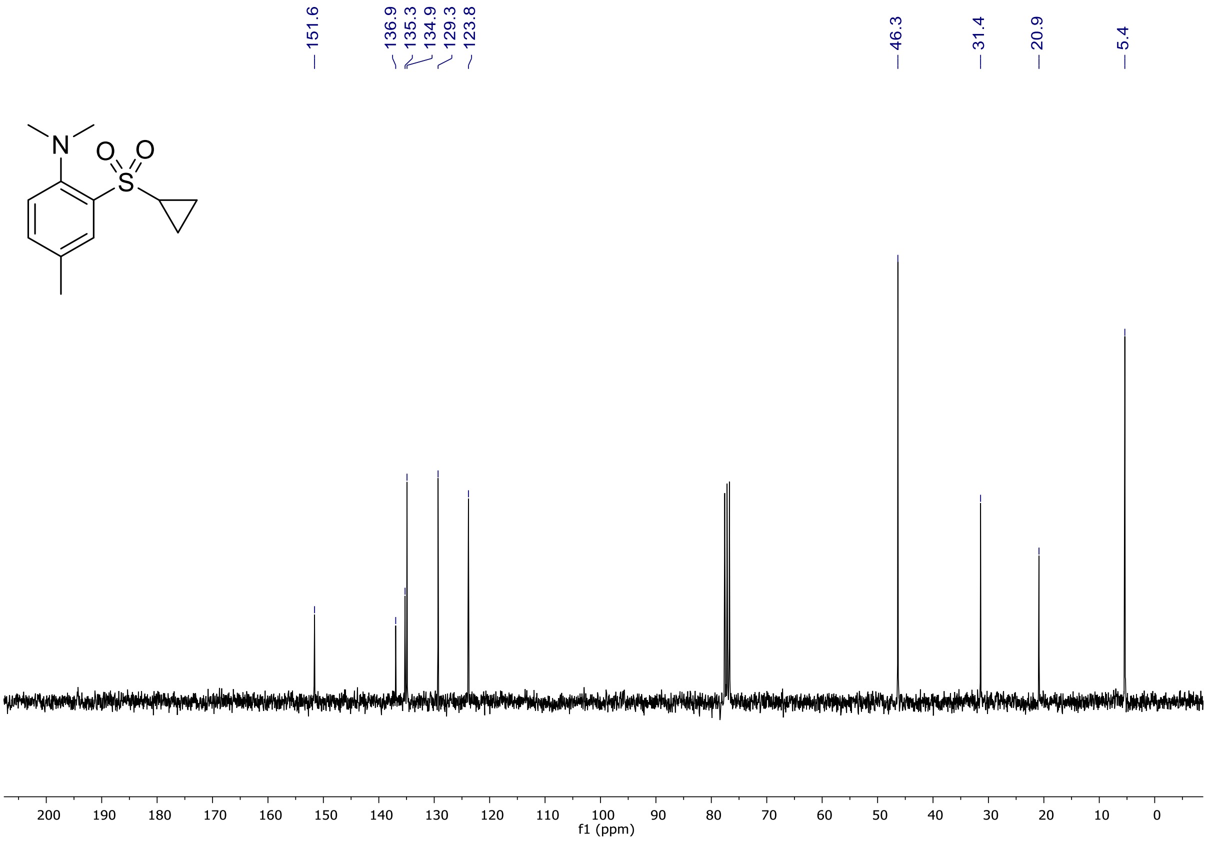

^{1}H NMR (300 MHz, $CDCl_3$) of **3k**

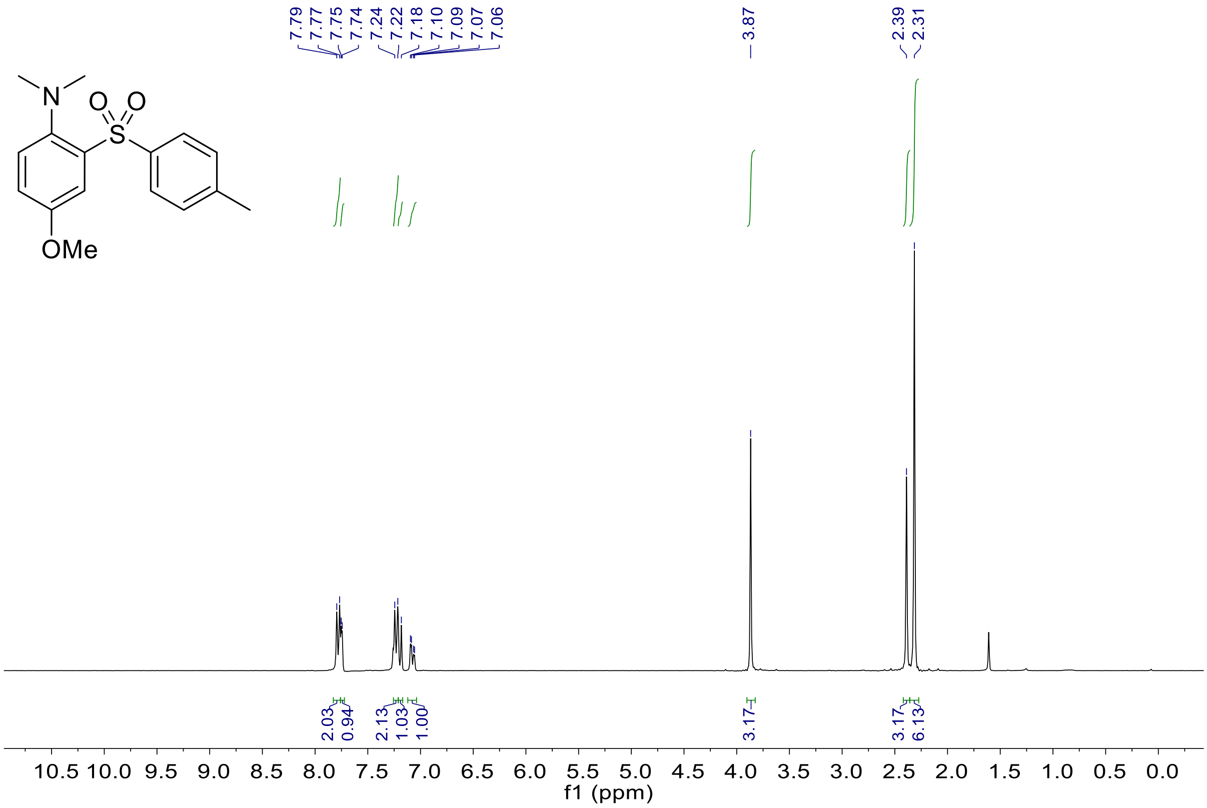

^{13}C NMR (75.5 MHz, $CDCl_3$) of **3k**

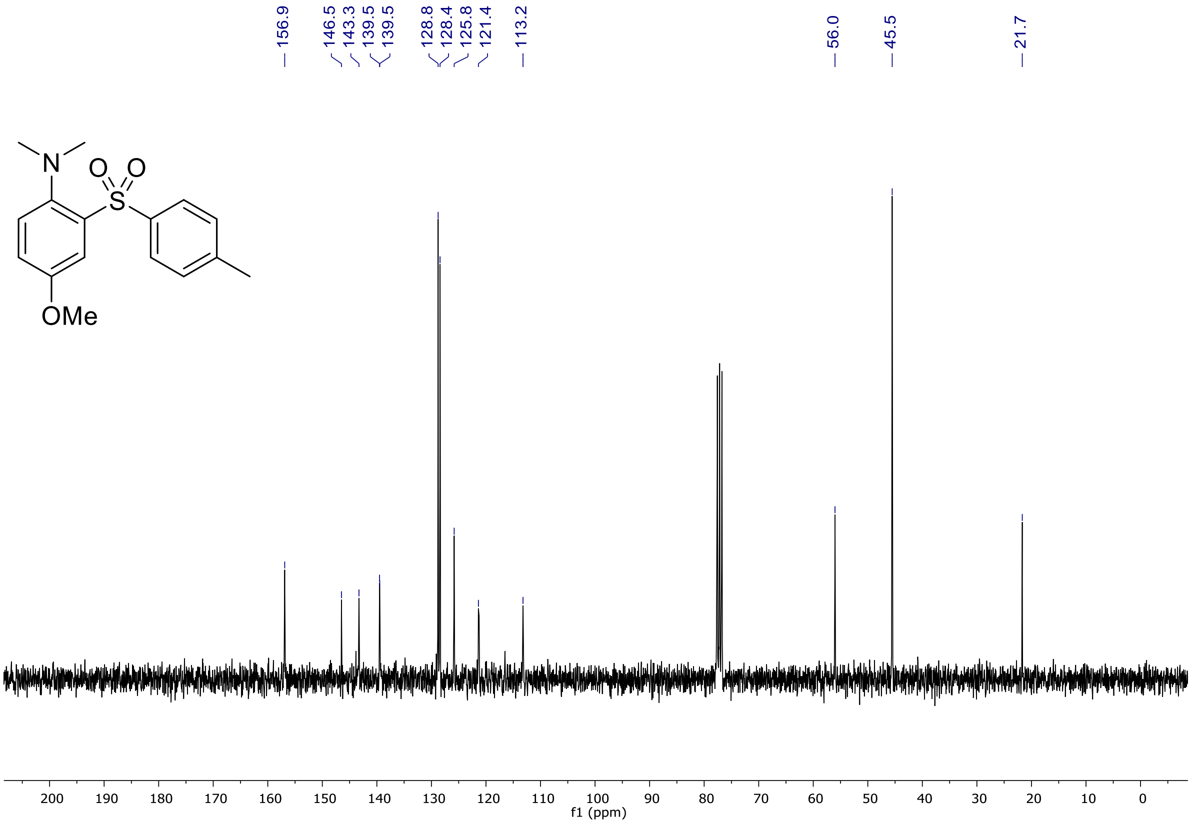

^{1}H NMR (300 MHz, $CDCl_3$) of **3l**

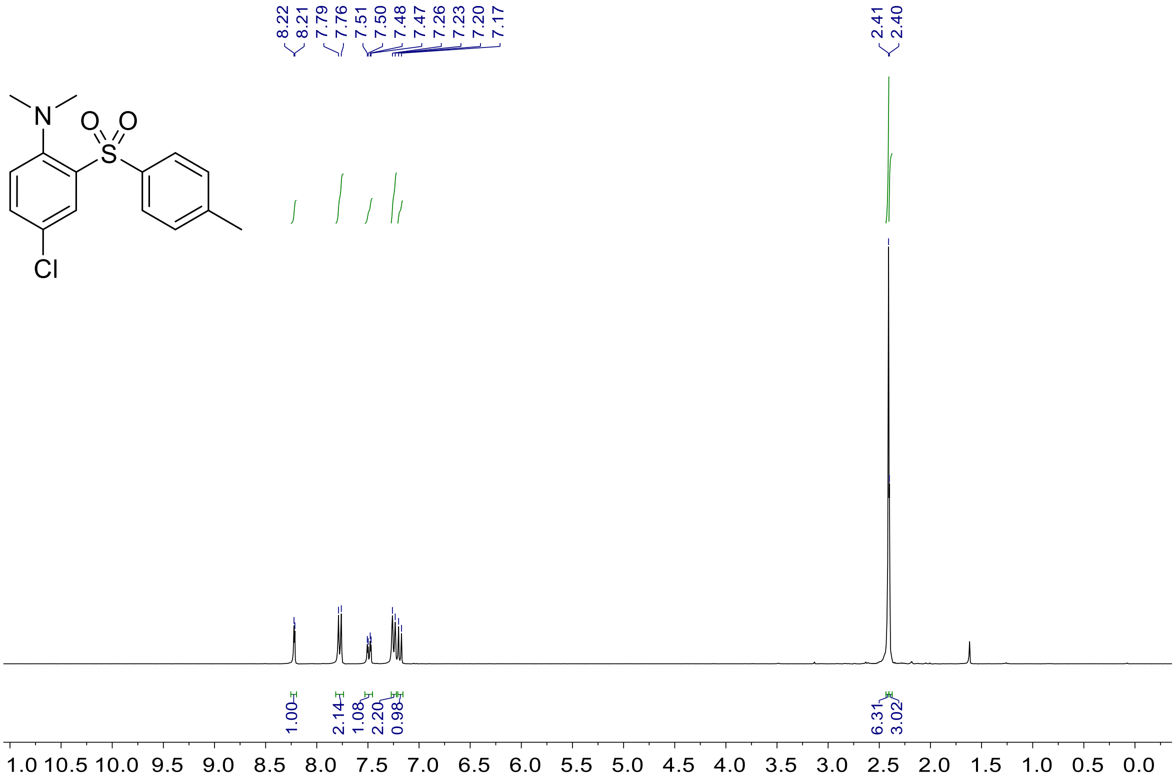

^{13}C NMR (75.5 MHz, $CDCl_3$) of **3l**

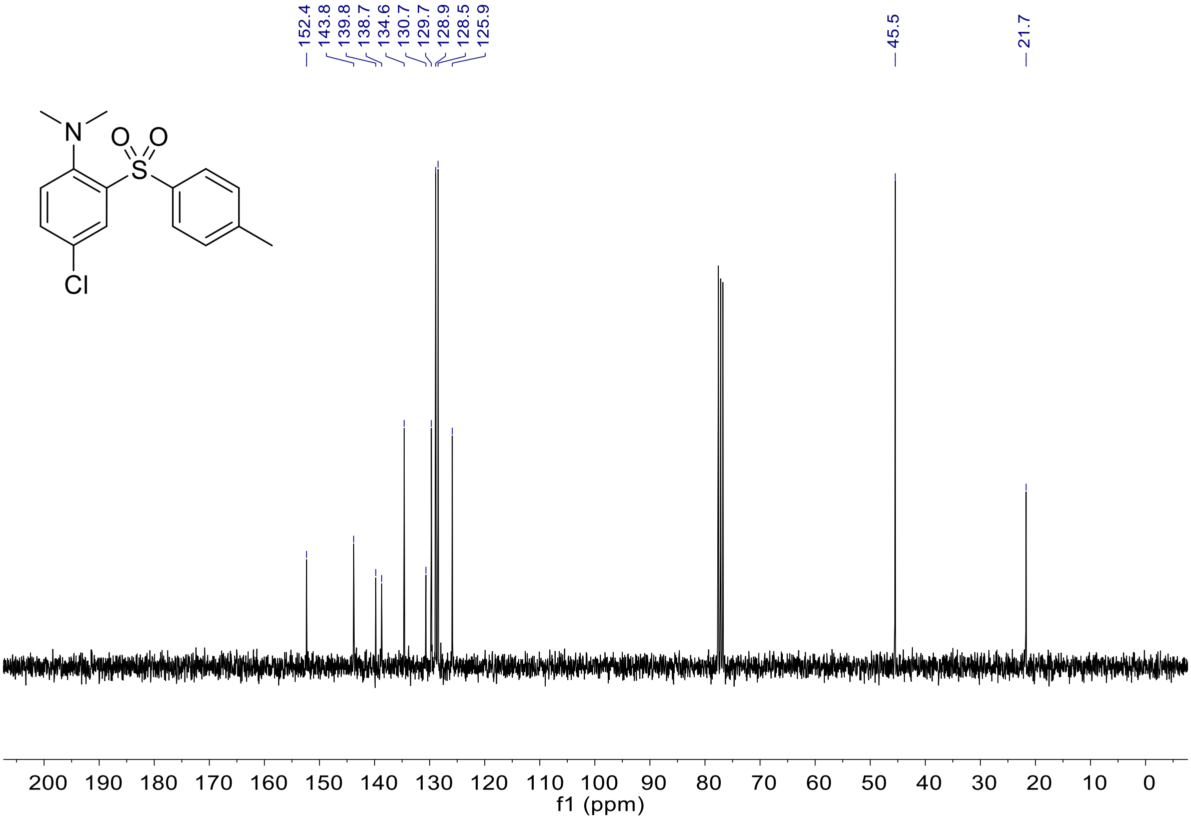

^{1}H NMR (300 MHz, $CDCl_3$) of **3m**

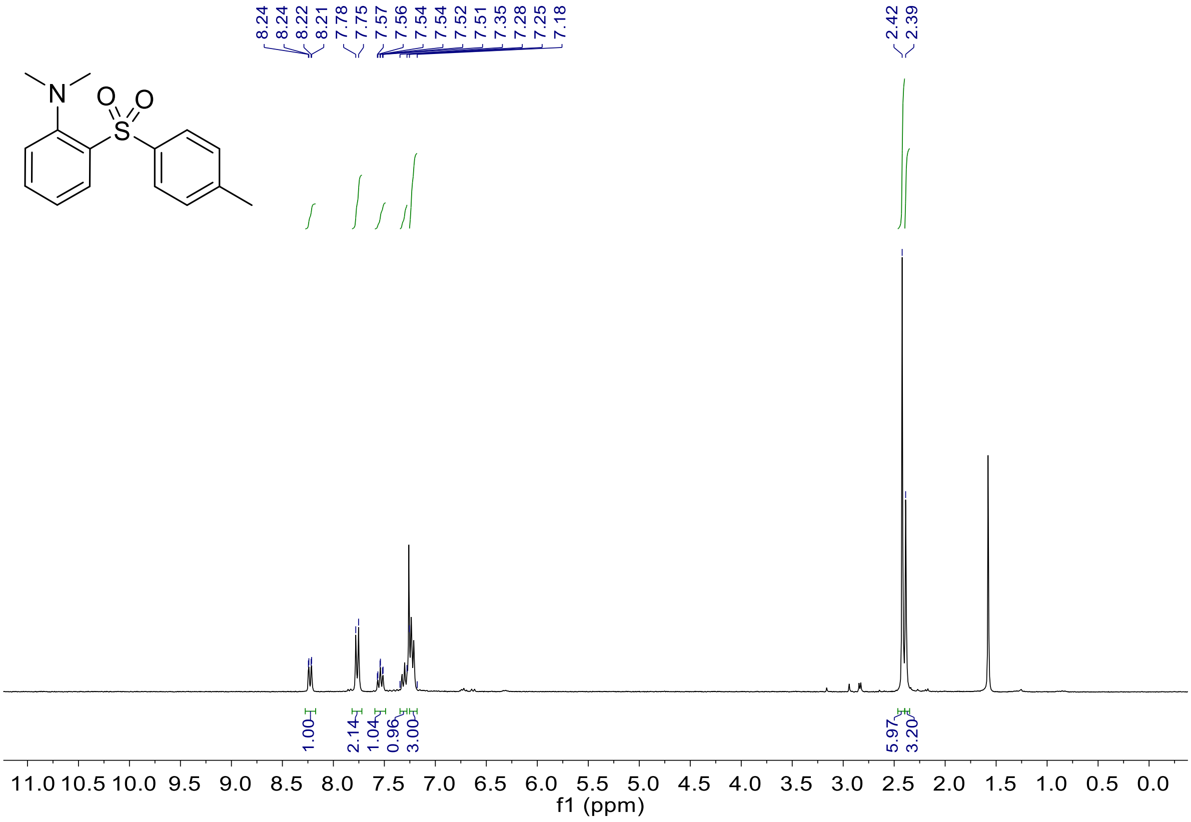

^{13}C NMR (126 MHz, $CDCl_3$) of **3m**

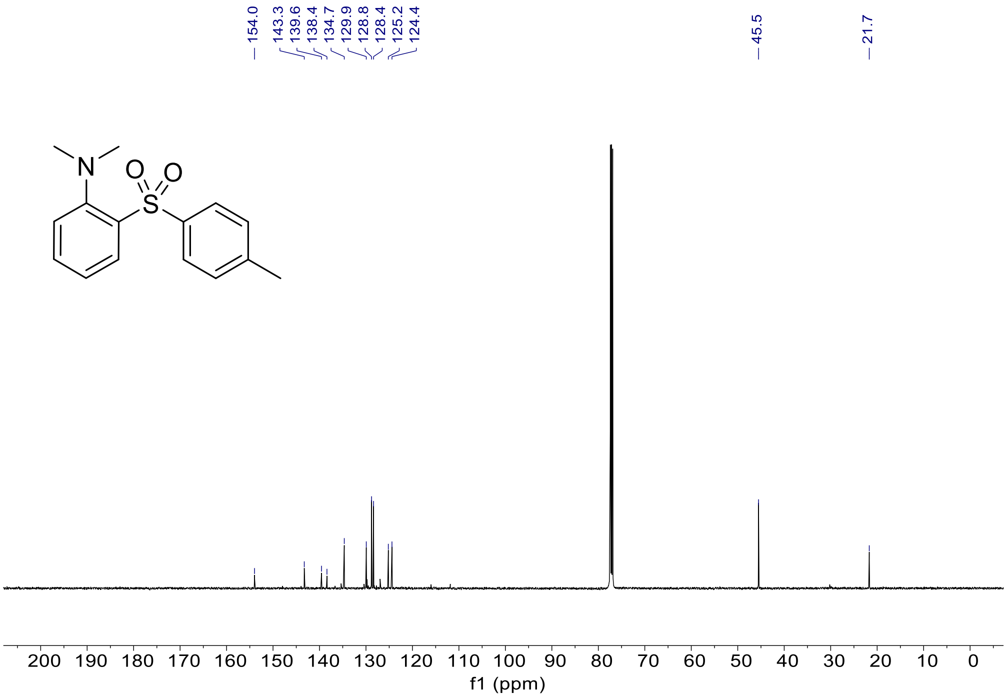

^{1}H NMR (300 MHz, $CDCl_3$) of **3m´**

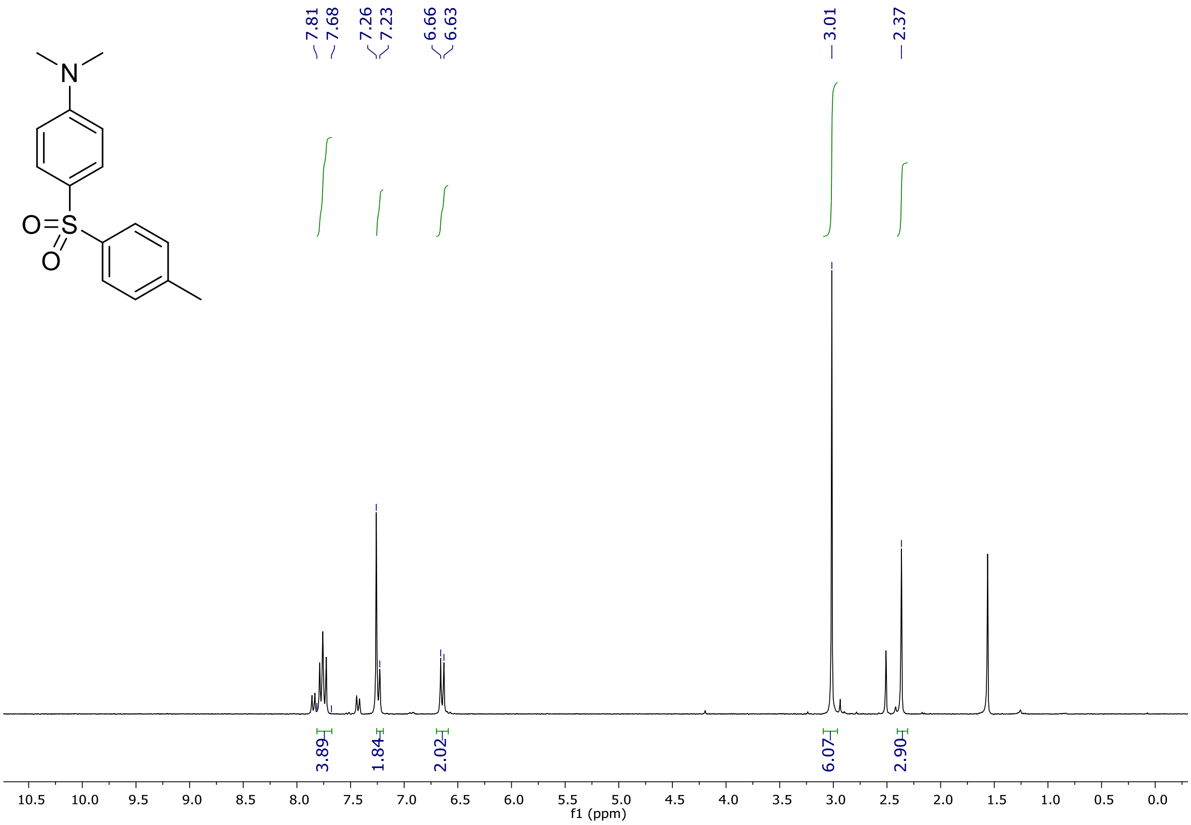

^{13}C NMR (126 MHz, $CDCl_3$) of **3m´**

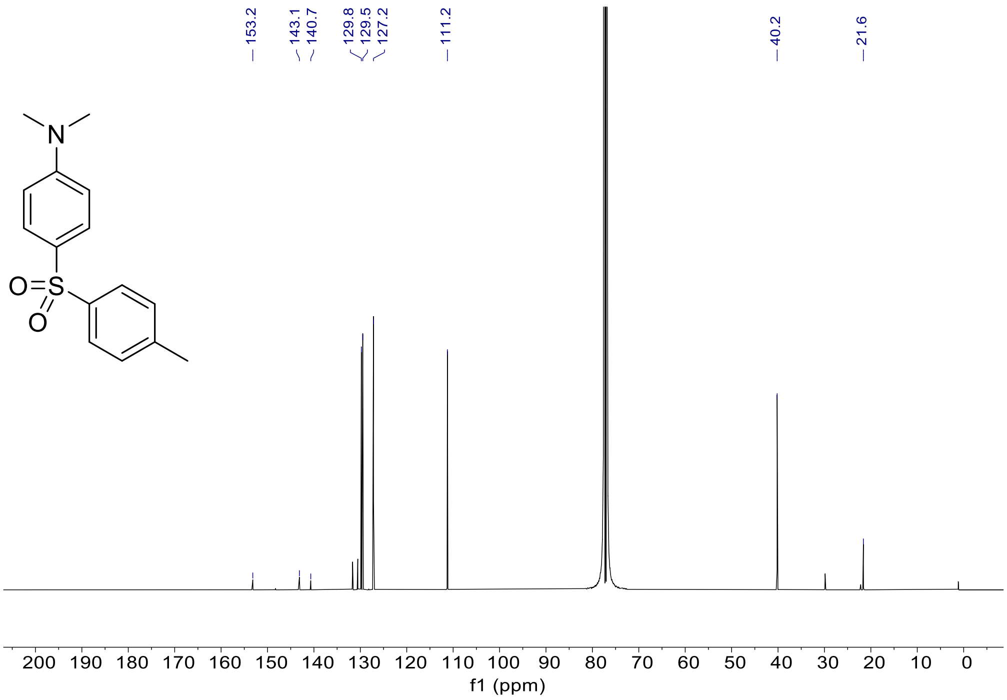

^{1}H NMR (300 MHz, $CDCl_3$) of **3n**

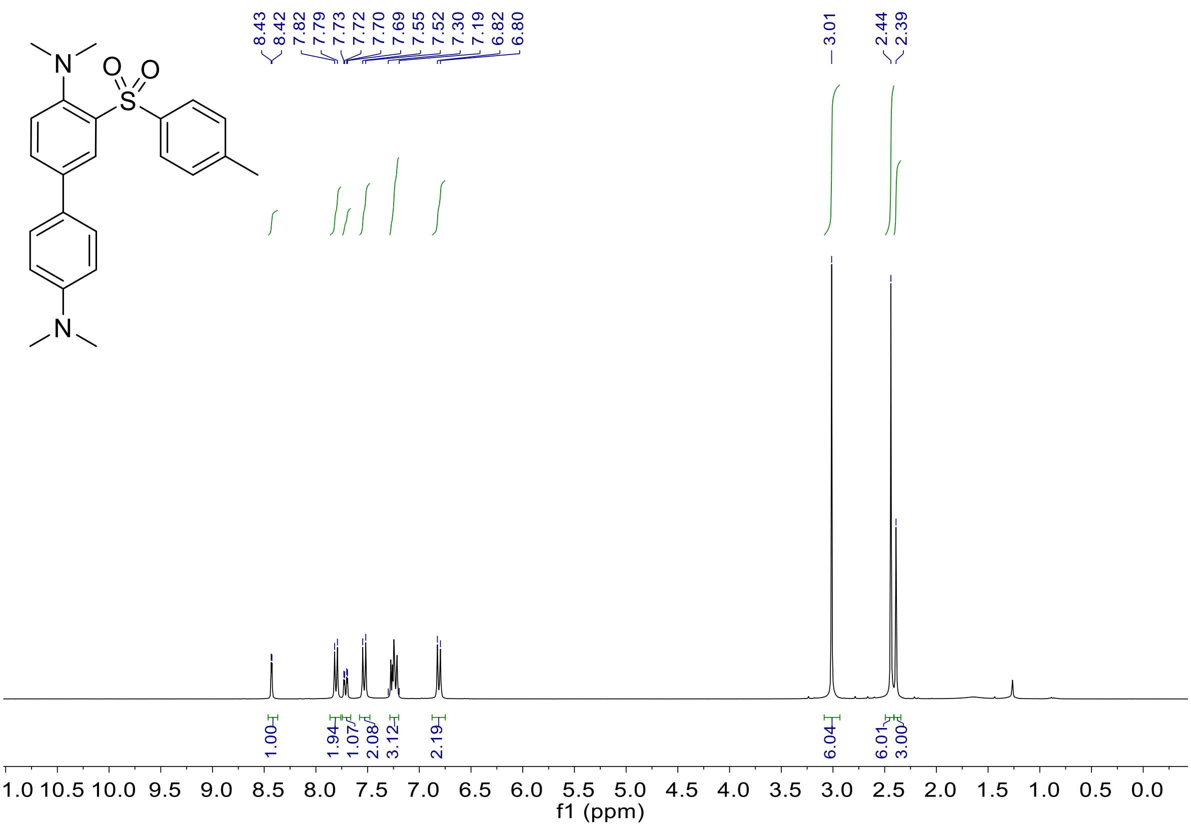

^{13}C NMR (75.5 MHz, $CDCl_3$) of **3n**

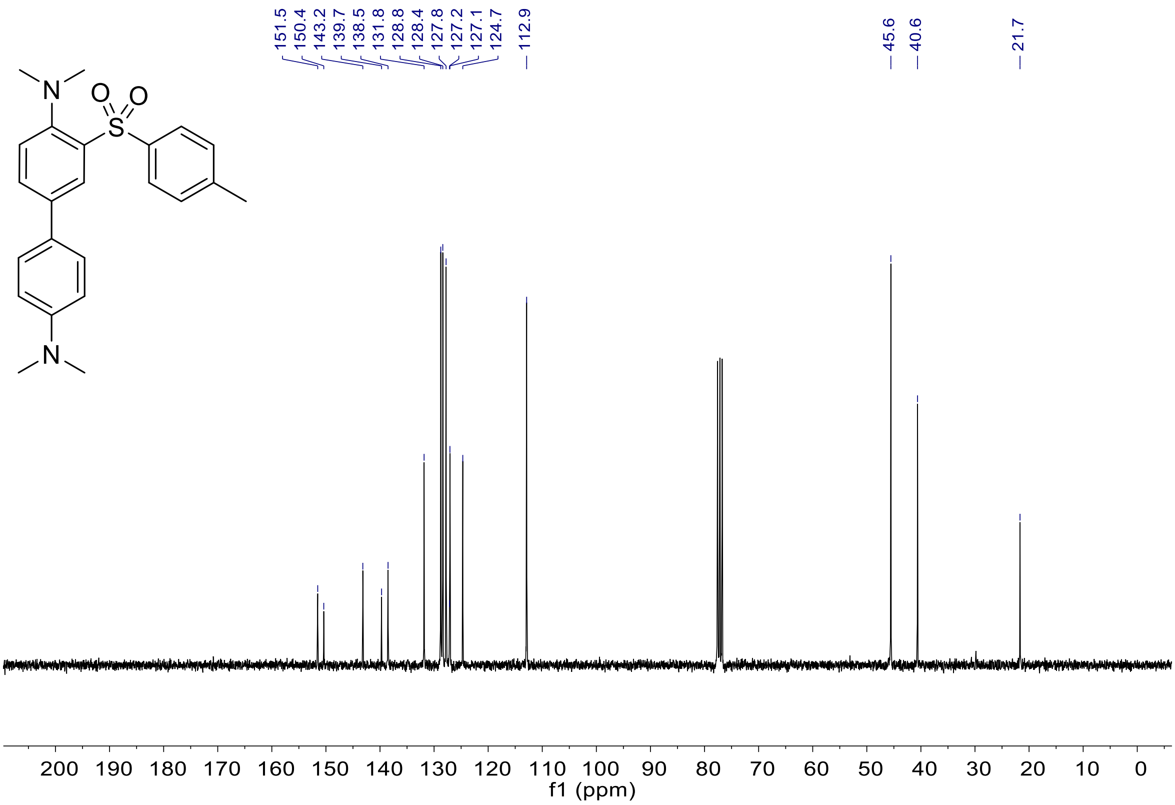

^{1}H NMR (300 MHz, $CDCl_3$) of **3o**

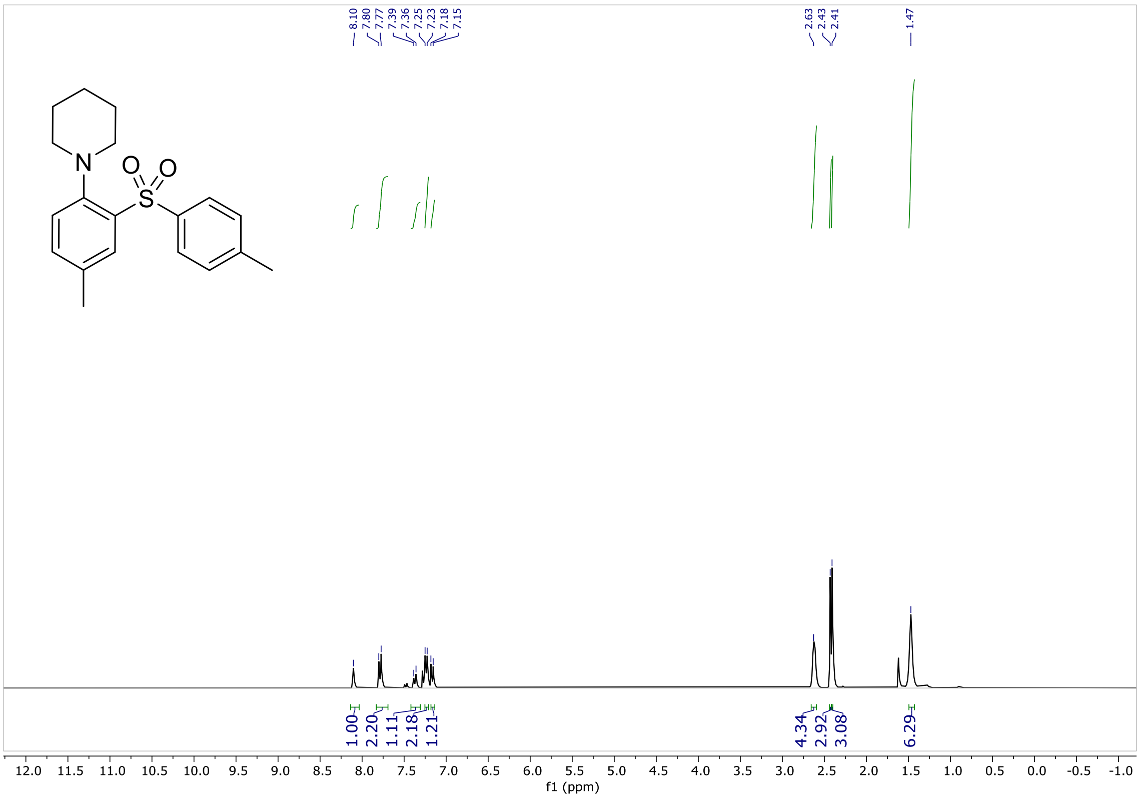

^{13}C NMR (126 MHz, $CDCl_3$) of **3o**

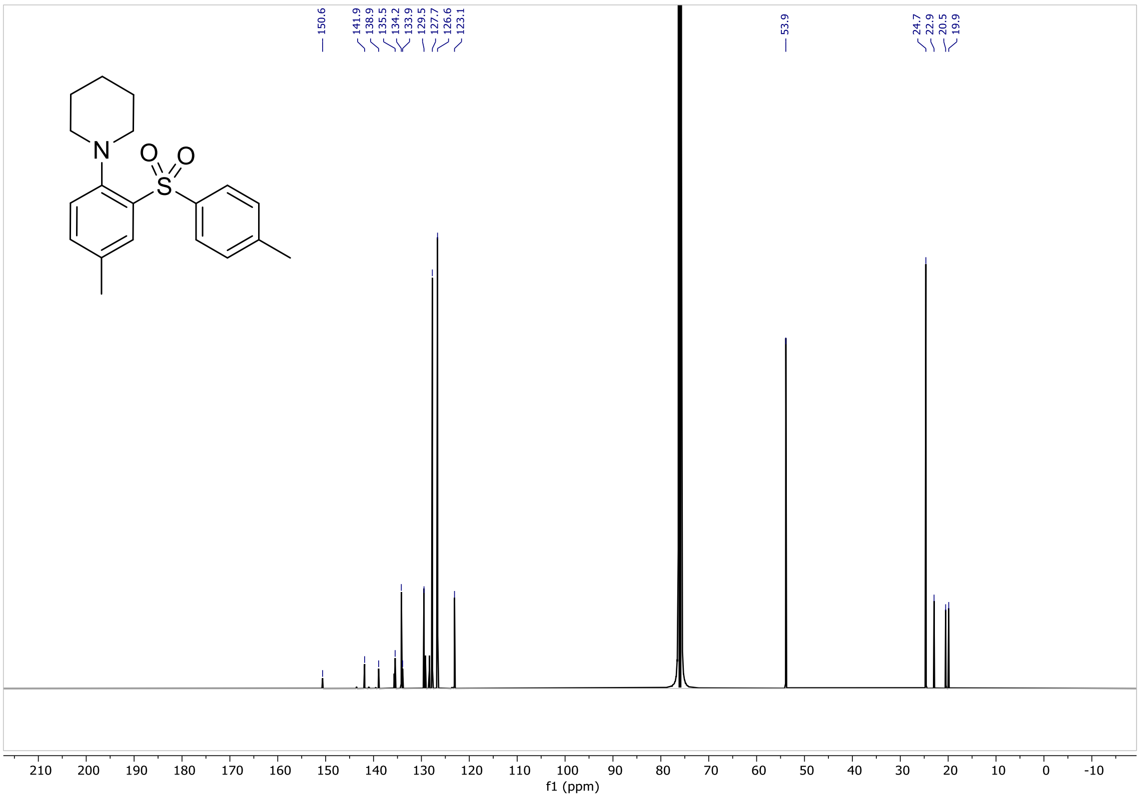

^{1}H NMR (300 MHz, $CDCl_3$) of **3p**

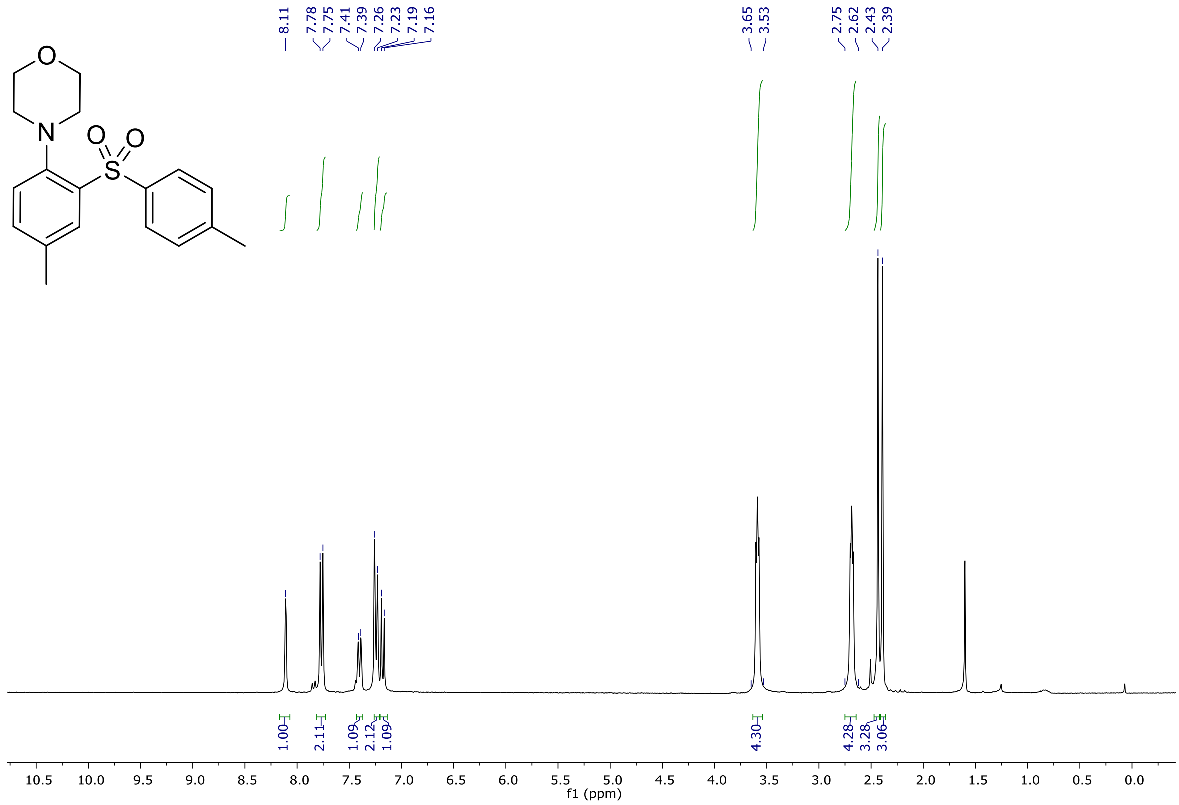

^{13}C NMR (75.5 MHz, $CDCl_3$) of **3p**

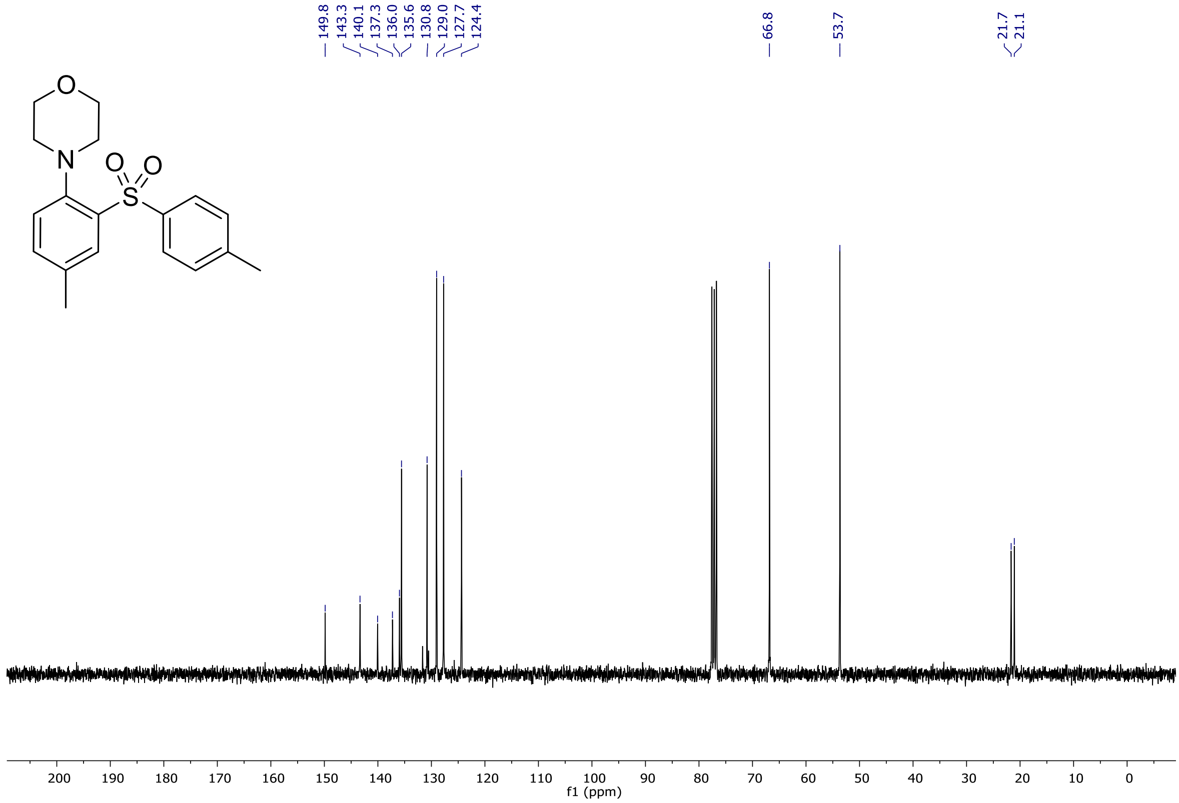

^{1}H NMR (300 MHz, $CDCl_3$) of 3q

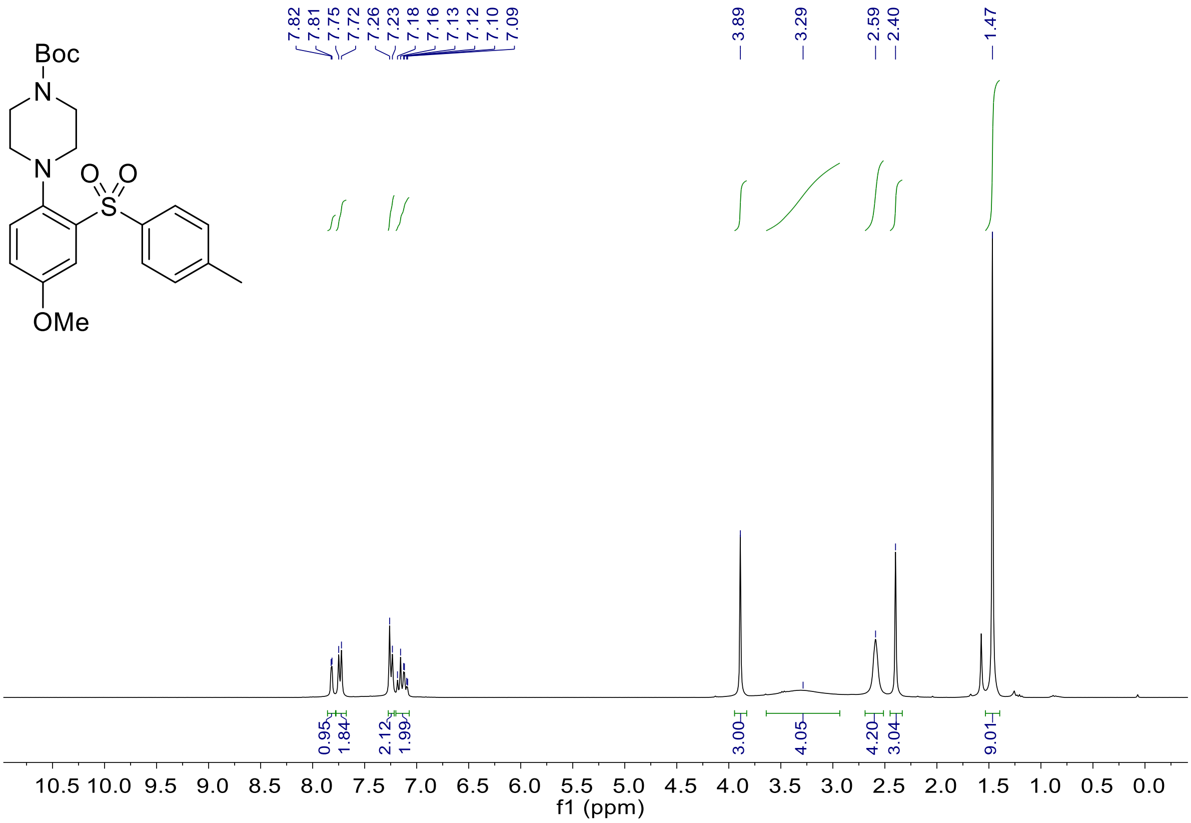

^{13}C NMR (75.5 MHz, $CDCl_3$) of 3q

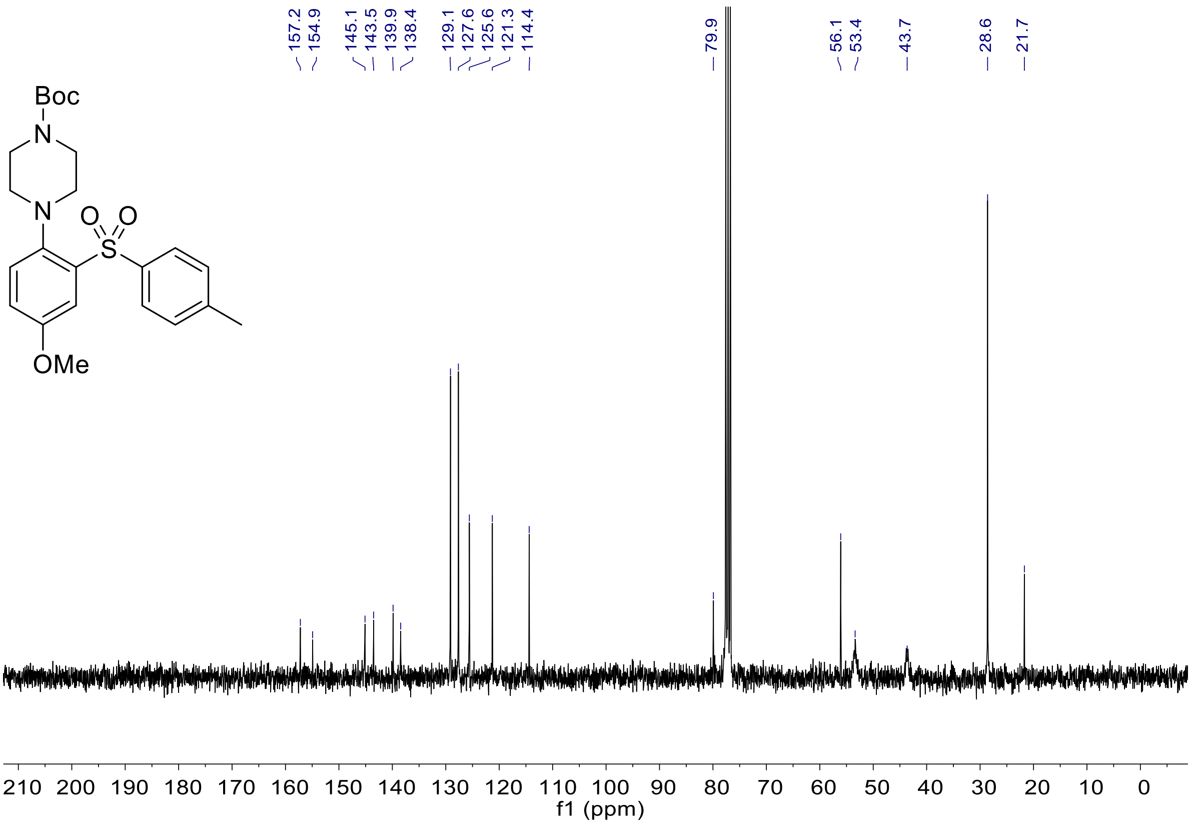

^{1}H NMR (300 MHz, $CDCl_3$) of **3r**

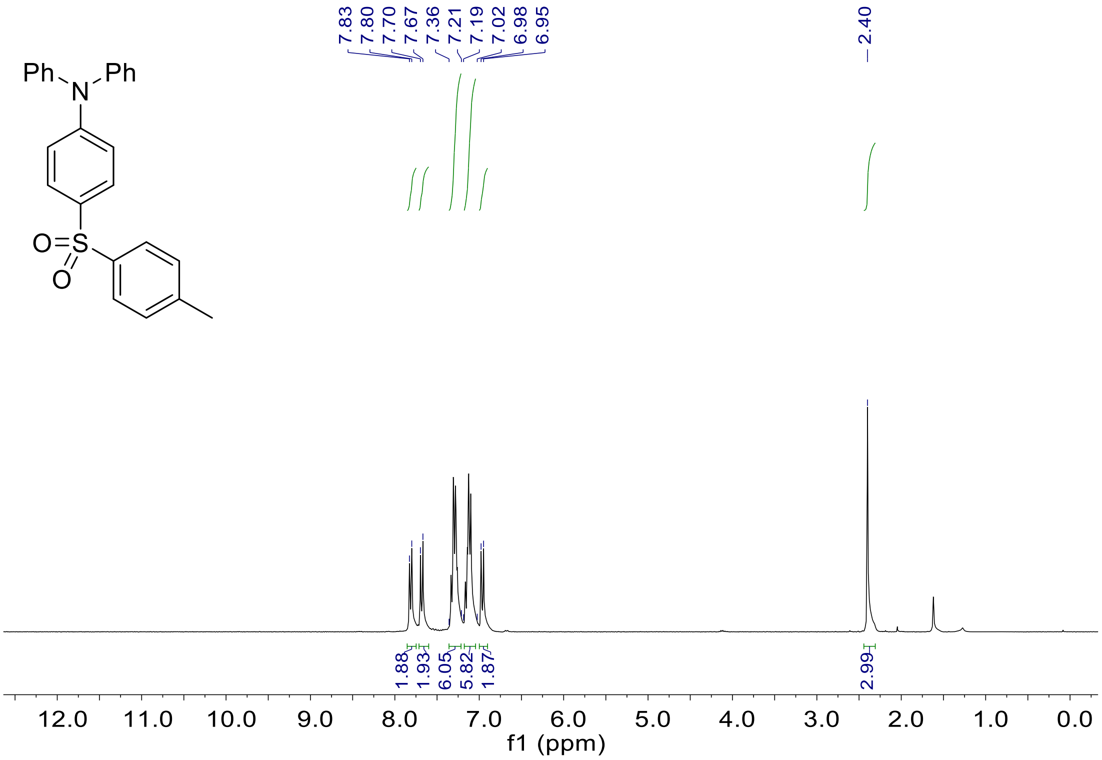

^{13}C NMR (75.5 MHz, $CDCl_3$) of **3r**

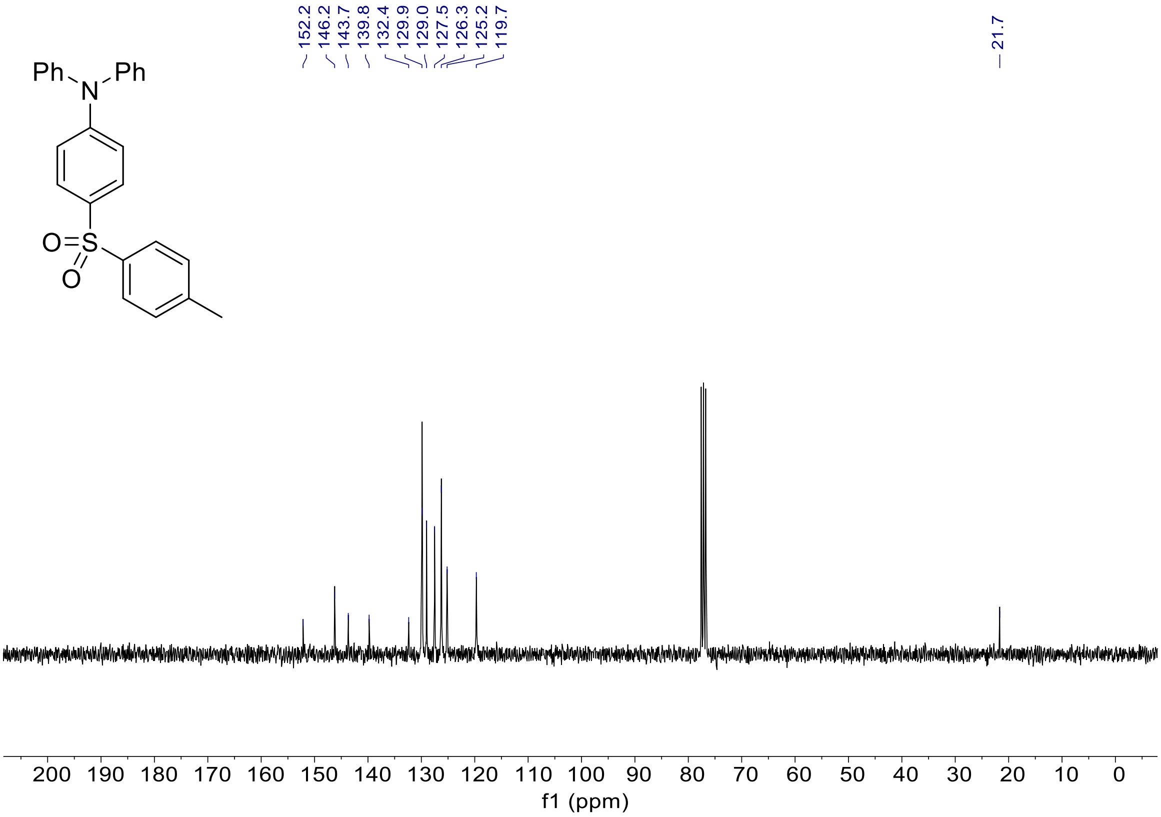

^{1}**H NMR** (300 MHz, $CDCl_3$) of **3s**

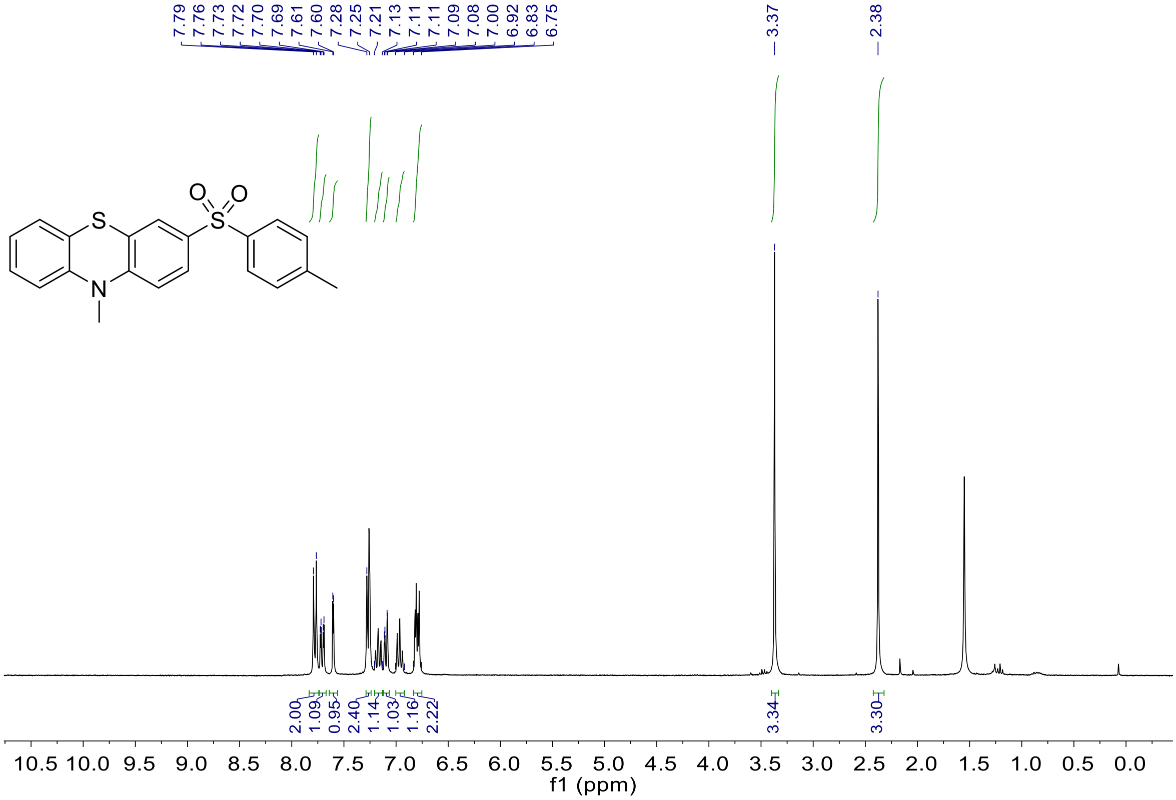

^{13}**C NMR** (75.5 MHz, $CDCl_3$) of **3s**

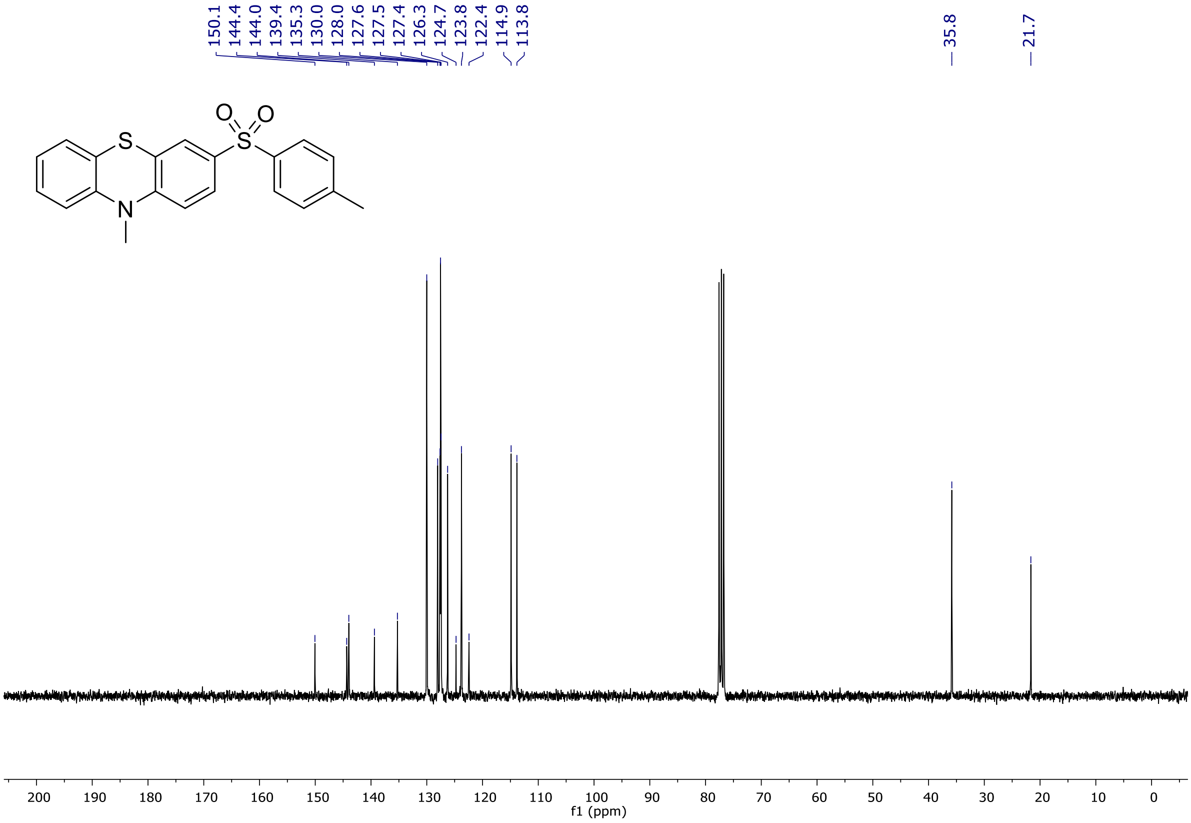

^{1}H NMR (500 MHz, $CDCl_3$) of **3t**

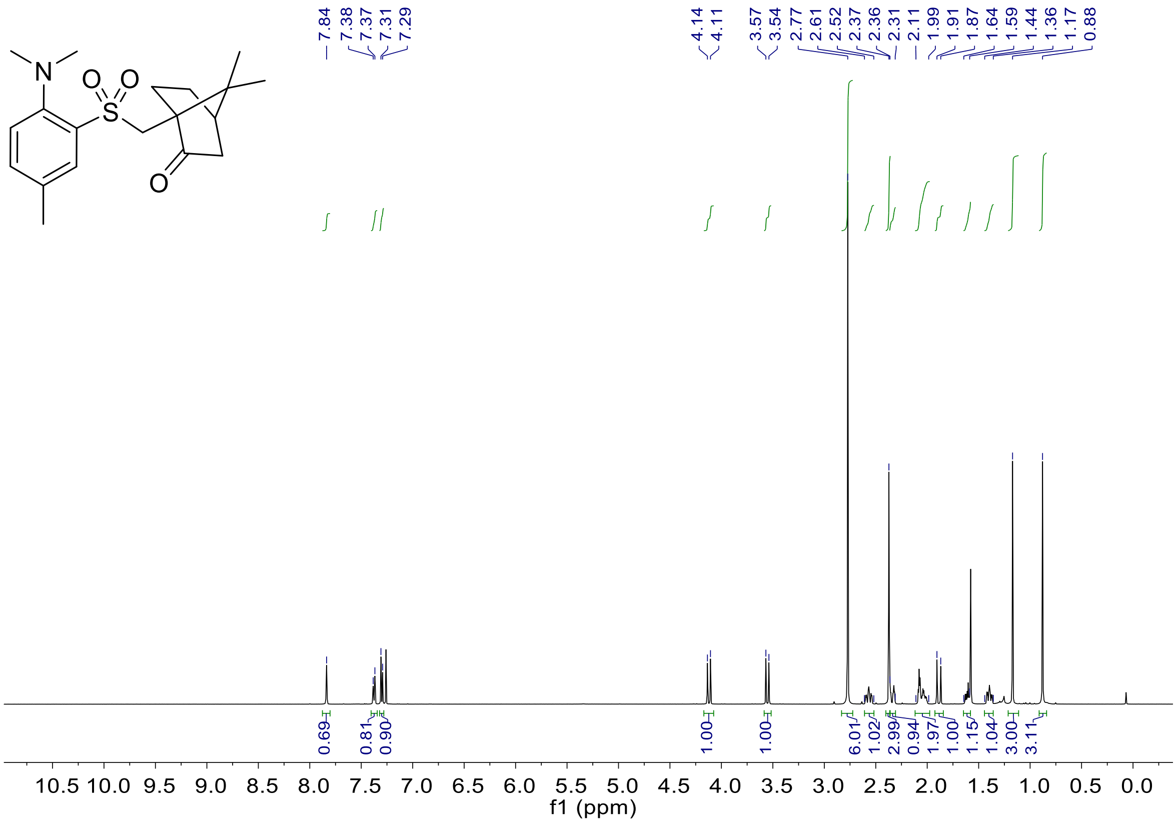

^{13}C NMR (126 MHz, $CDCl_3$) of **3t**

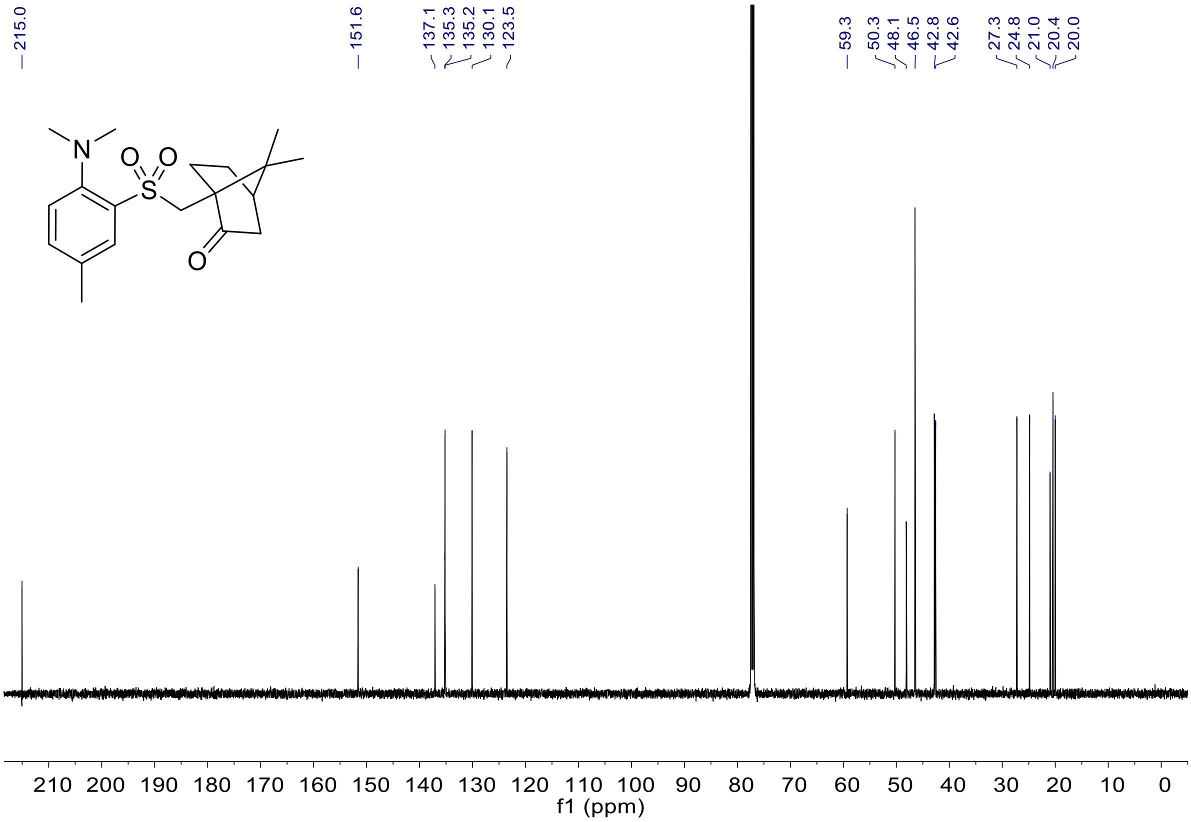

^{1}H NMR (300 MHz, $CDCl_3$) of **3u**

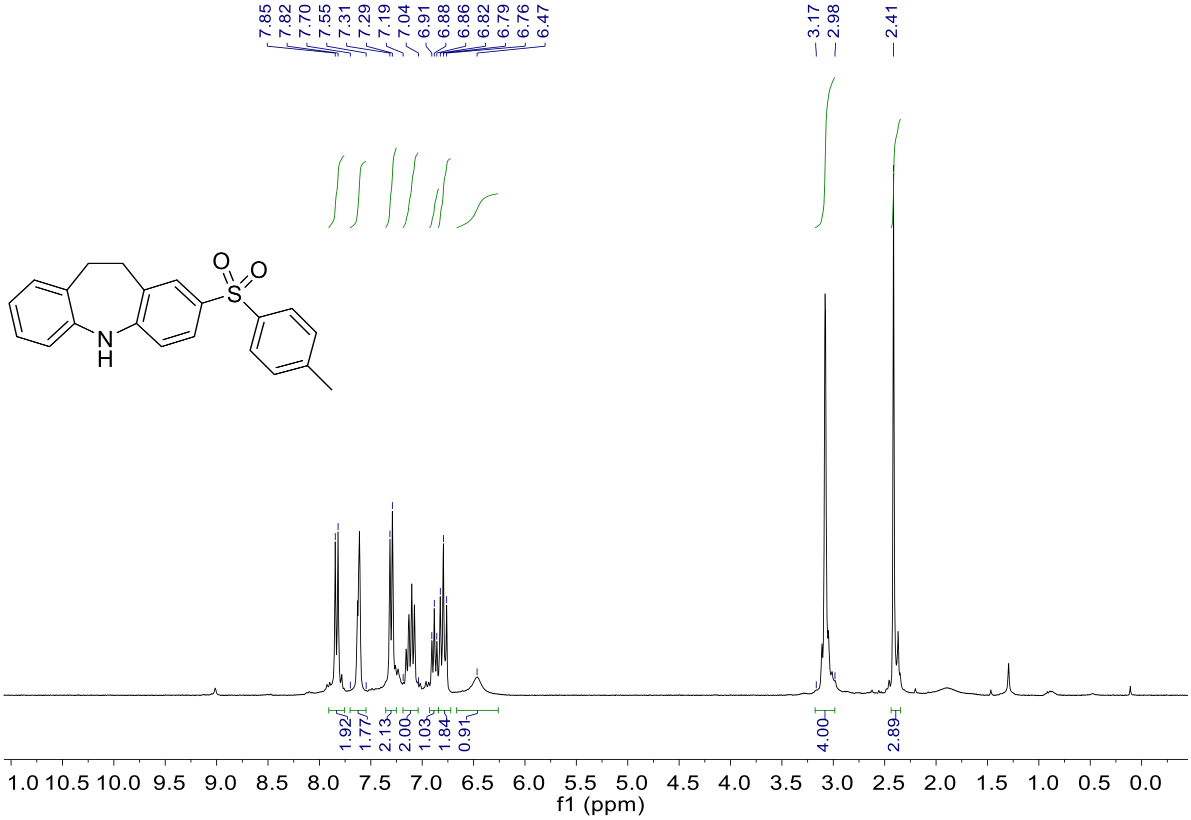

^{13}C NMR (75.5 MHz, $CDCl_3$) of **3u**

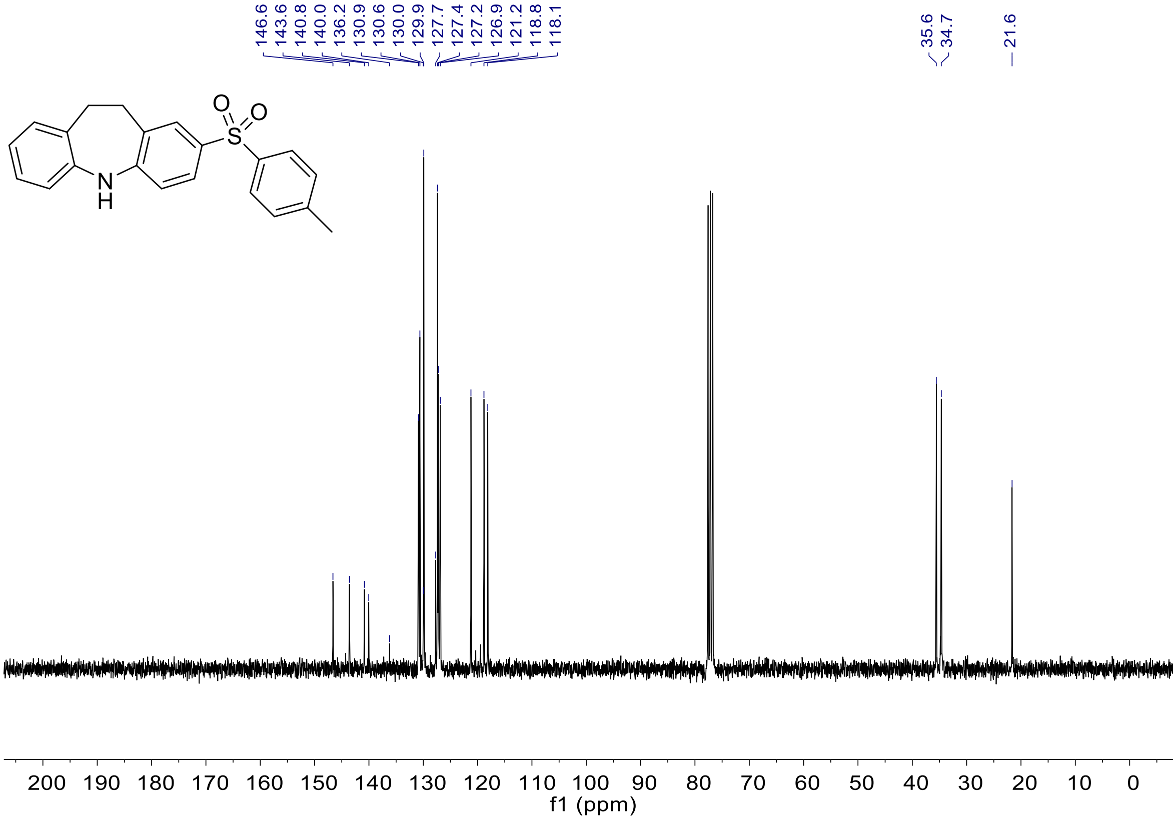

^{1}H NMR (300 MHz, $CDCl_3$) of **3v**

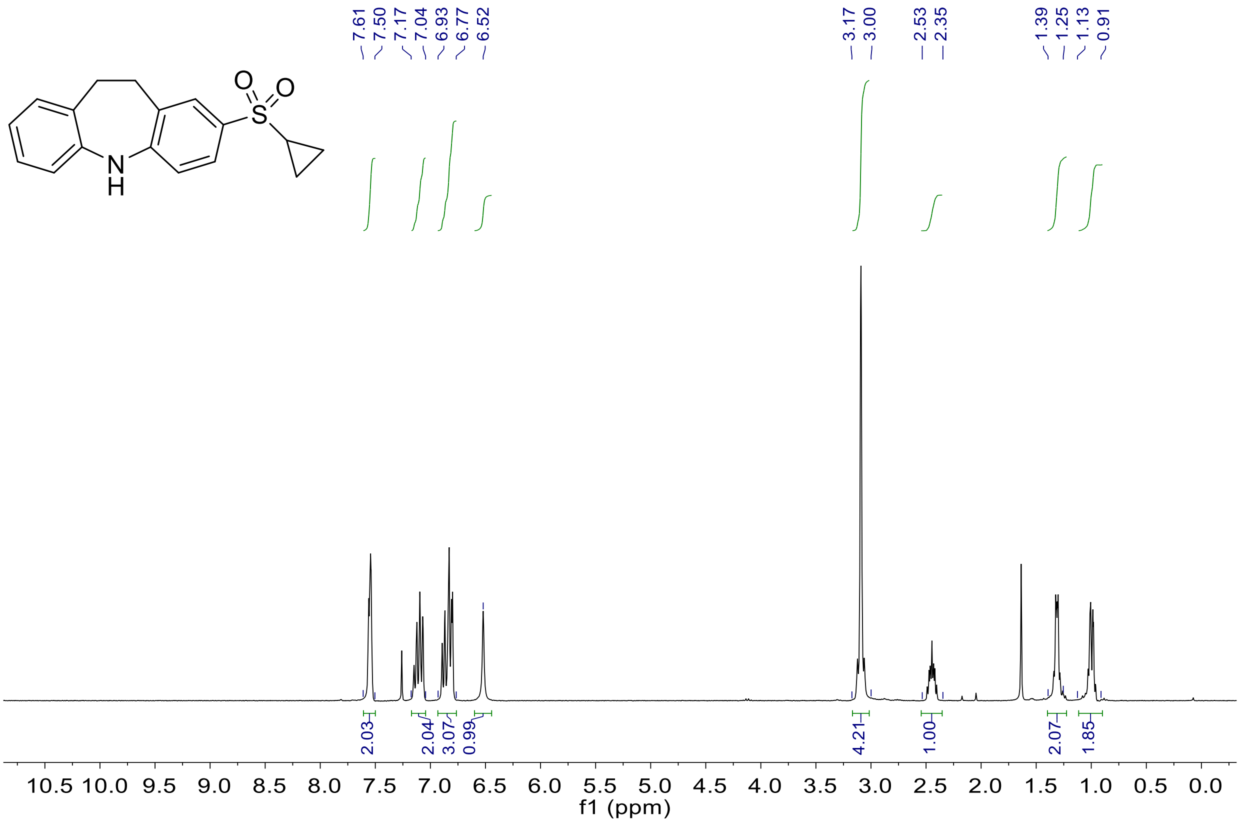

^{13}C NMR (75.5 MHz, $CDCl_3$) of **3v**

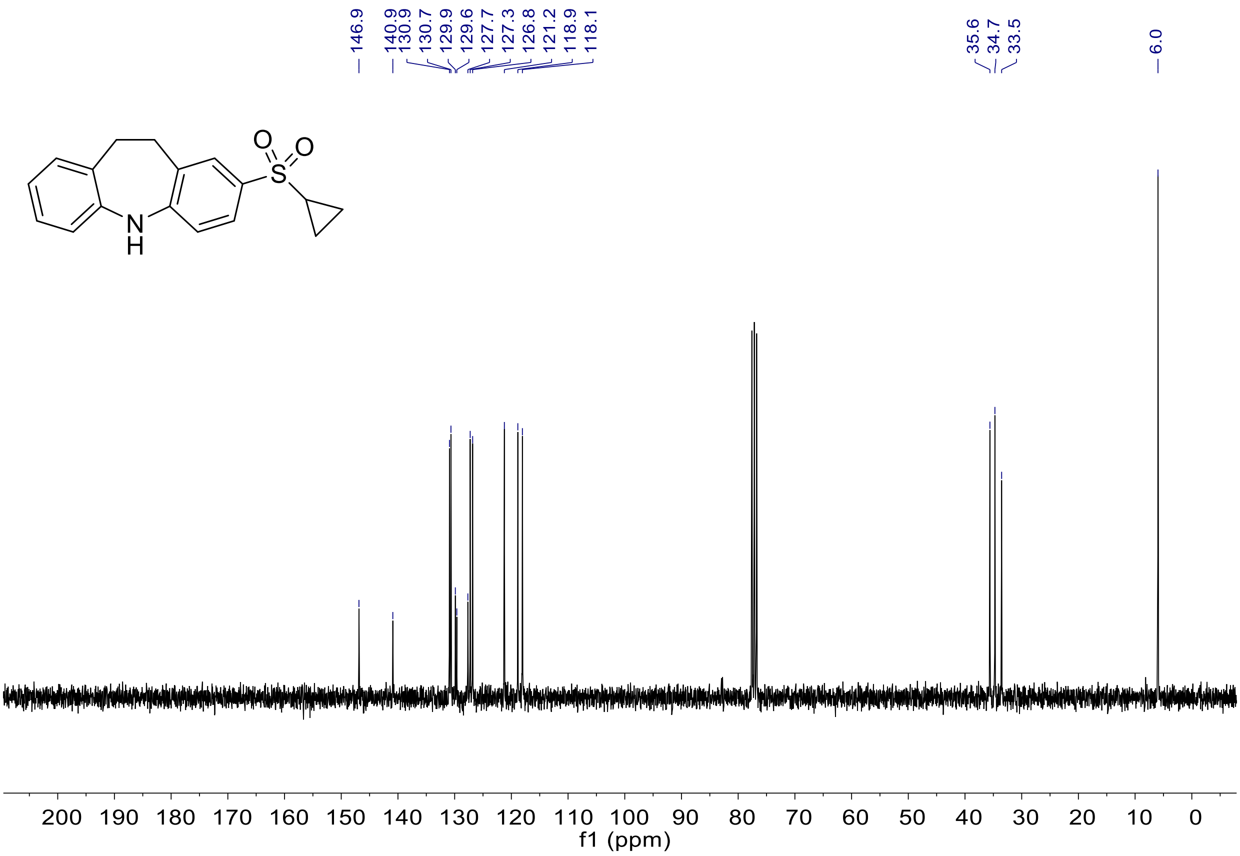

^{1}H NMR (300 MHz, $CDCl_3$) of **3w**

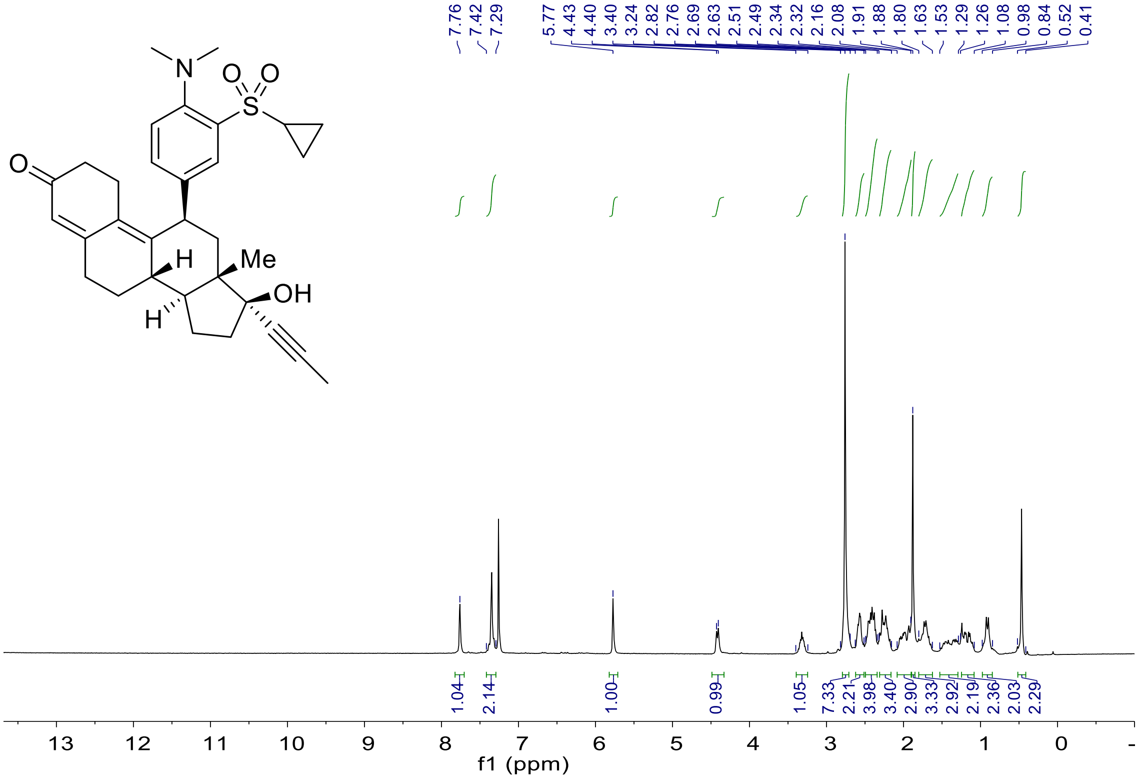

^{13}C NMR (126 MHz, $CDCl_3$) of **3w**

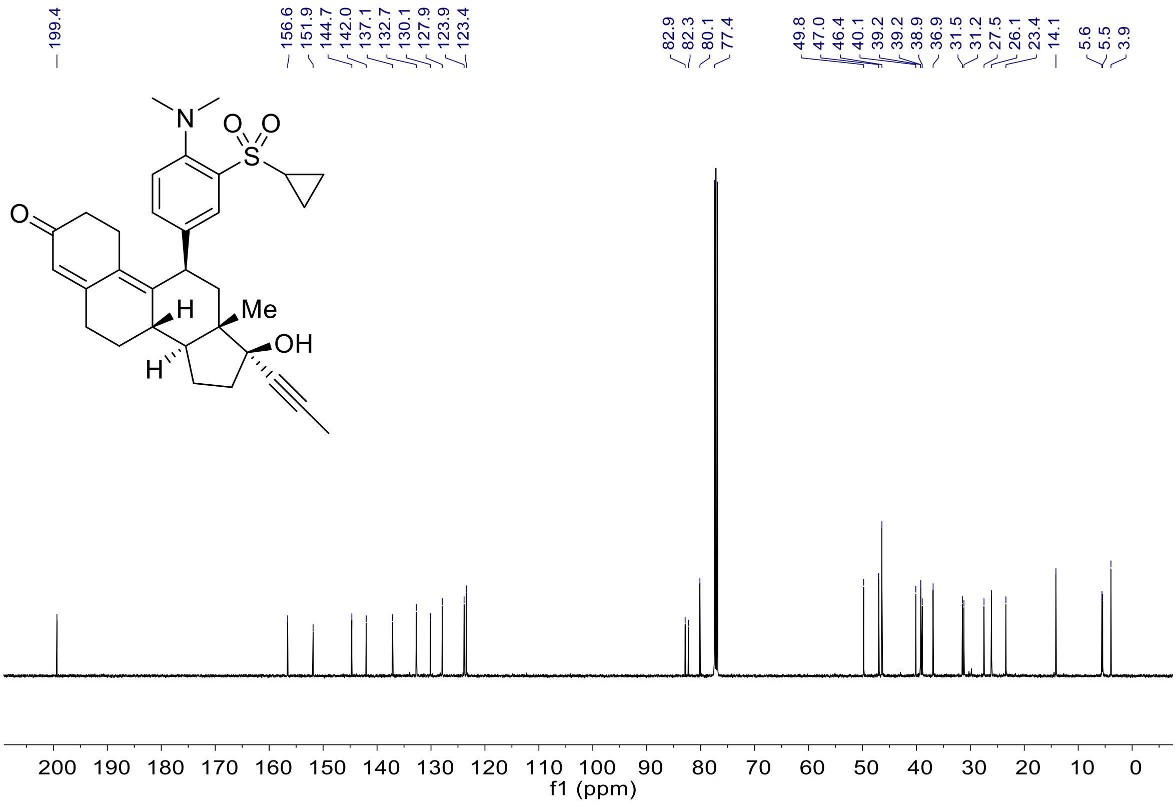

^{1}**H NMR** (300 MHz, $CDCl_3$) of **3x** and **3x´**

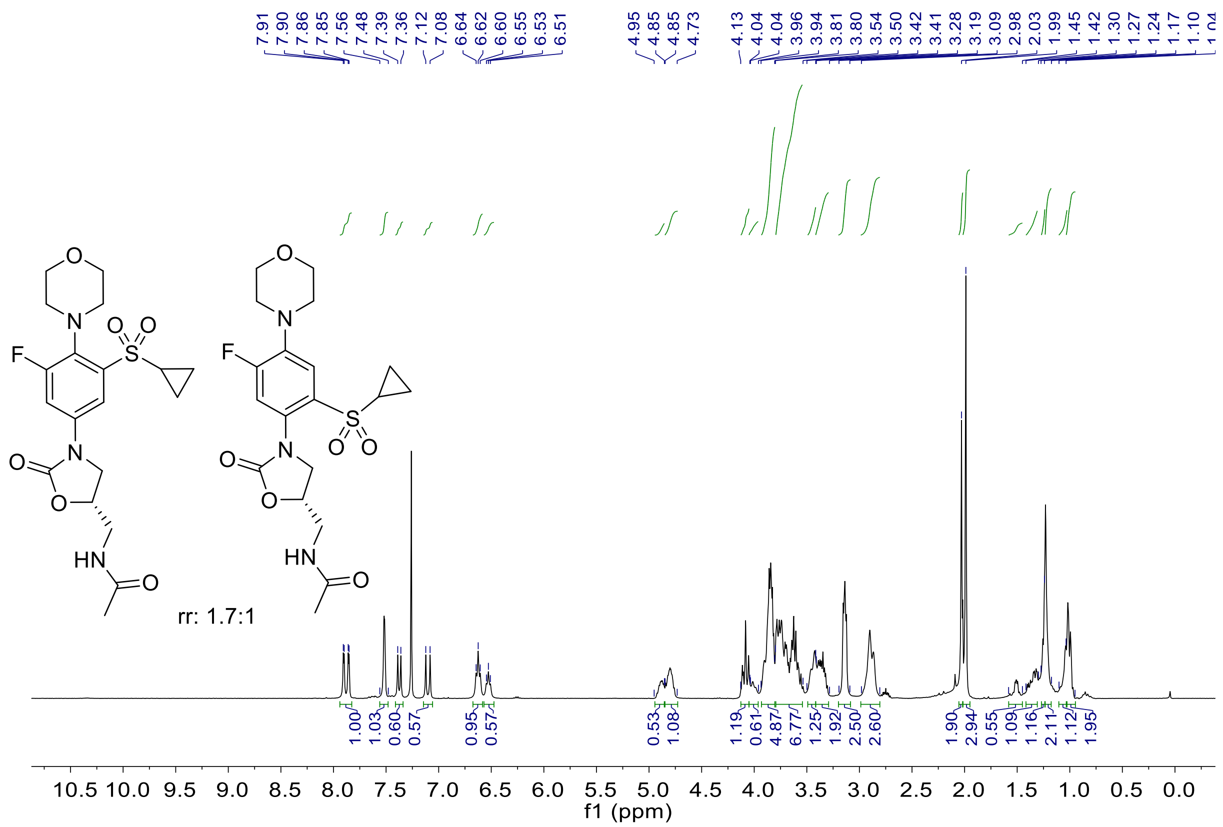

^{13}**C NMR** (126 MHz, $CDCl_3$) of **3x** and **3x´**

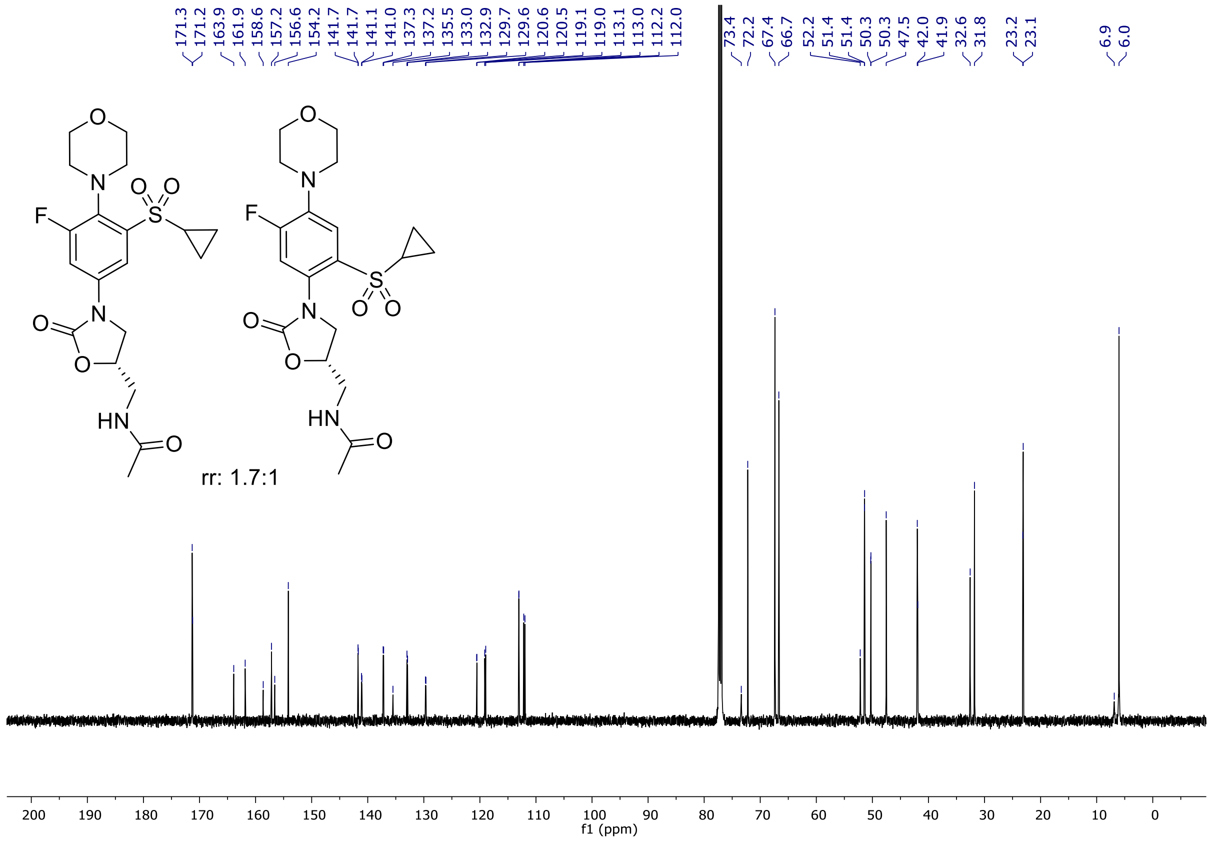

^{19}F NMR (282 MHz, $CDCl_3$) of **3x** and **3x′**

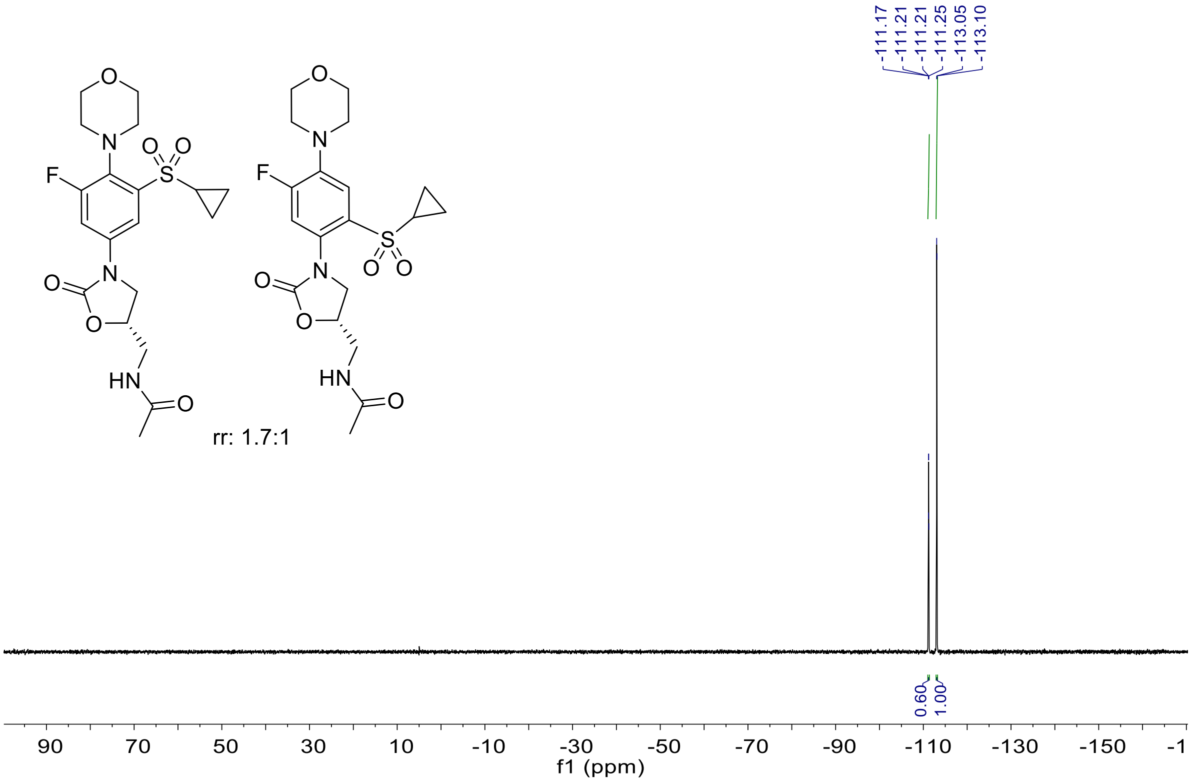

^{1}H-^{13}C HSQC (300 MHz, $CDCl_3$) of **3x** and **3x′**

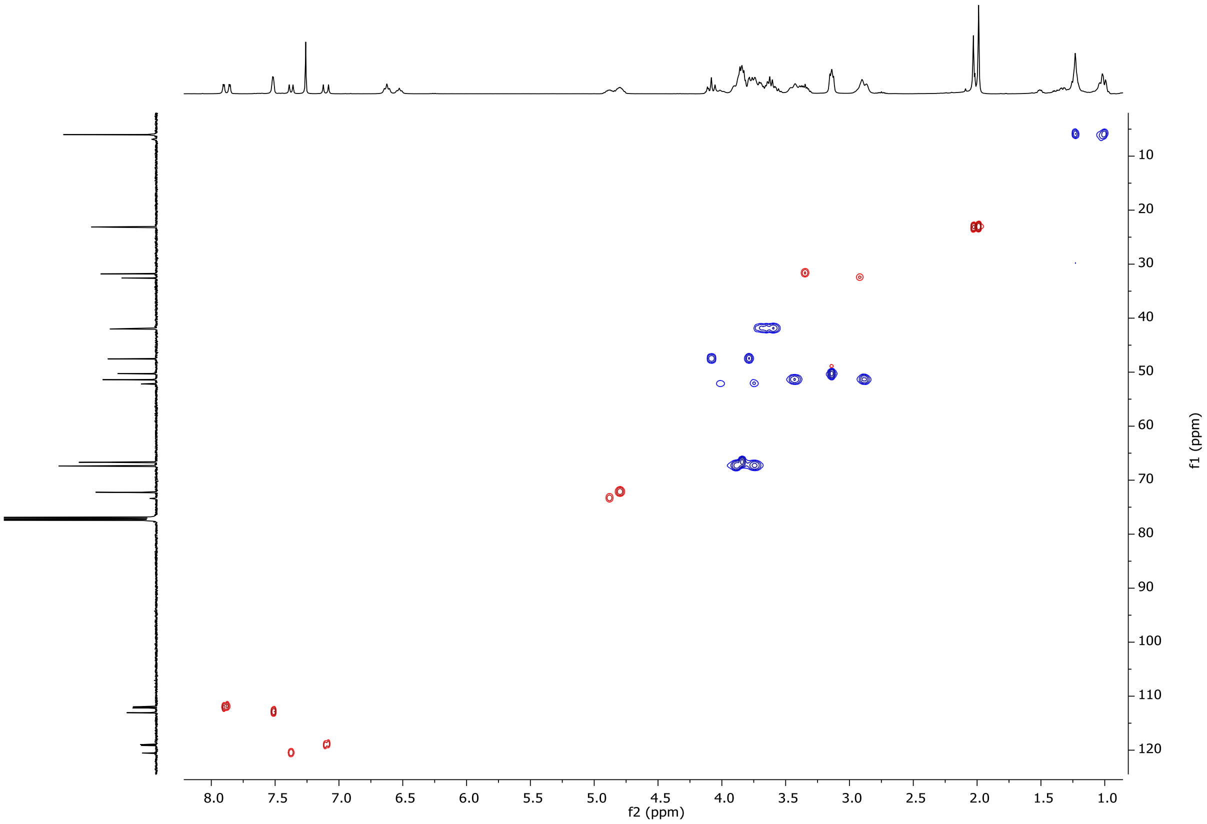

Supplement: Supplementary file 1 [file ja5c12697_si_001.pdf]
